# Flexible mapping of homology onto structure with Homolmapper

**DOI:** 10.1186/1471-2105-8-123

**Published:** 2007-04-11

**Authors:** Nathan C Rockwell, J Clark Lagarias

**Affiliations:** 1 Section of Molecular and Cellular Biology, University of California, Davis, California 95616, USA

## Abstract

**Background:**

Over the past decade, a number of tools have emerged for the examination of homology relationships among protein sequences in a structural context. Most recent software implementations for such analysis are tied to specific molecular viewing programs, which can be problematic for collaborations involving multiple viewing environments. Incorporation into larger packages also adds complications for users interested in adding their own scoring schemes or in analyzing proteins incorporating unusual amino acid residues such as selenocysteine.

**Results:**

We describe homolmapper, a command-line application for mapping information from a multiple protein sequence alignment onto a protein structure for analysis in the viewing software of the user's choice. Homolmapper is small (under 250 K for the application itself) and is written in Python to ensure portability. It is released for non-commercial use under a modified University of California BSD license. Homolmapper permits facile import of additional scoring schemes and can incorporate arbitrary additional amino acids to allow handling of residues such as selenocysteine or pyrrolysine. Homolmapper also provides tools for defining and analyzing subfamilies relative to a larger alignment, for mutual information analysis, and for rapidly visualizing the locations of mutations and multi-residue motifs.

**Conclusion:**

Homolmapper is a useful tool for analysis of homology relationships among proteins in a structural context. There is also extensive, example-driven documentation available. More information about homolmapper is available at .

## Background

The proliferation of known or suspected protein sequences in the post-genomic era and the slower, but steady, progress in protein structure determination implies that there are a large number of proteins with no experimentally determined structure but with varying homology to a protein of known structure. When such proteins are of experimental or practical interest, several approaches can be used to generate some form of approximate structural information to aid in the design or interpretation of biochemical experiments. For example, homology modeling can be used to build a model structure for the protein of interest based on the known structure. First introduced in 1969 [1], homology modeling has become a more commonly applied tool in recent years. However, homology modeling remains sensitive not only to the multiple sequence alignment (MSA) of homologous proteins, but also to the particular algorithms used in the structural modeling [2]. Moreover, the confidence assigned to such models is dependent on the evolutionary relatedness of the modeling target to the known template.

An alternative approach is to map information from the MSA onto the known structure, typically via color-coding or similar visual procedures. Such combinations of structural and sequence information do not contain as much information as carefully made homology models, but they permit many of the same applications as homology modeling, such as predicting the location and/or function of potentially interesting residues, and are also useful in evaluating and modifying homology models themselves: a homology model that places a site of frequent gaps and insertions in the middle of a central beta strand rather than on a surface loop is unlikely to be reliable. Scoring the MSA onto the known structure is driven only by the MSA, without potential bias from the modeling or threading programs. This approach was formalized in 1995 with PDBALIGN [3], a command-line application taking as inputs a protein structure in PDB format and an MSA in GCG format. PDBALIGN generates a new PDB file retaining the original sequence and spatial information, but with homology information written to the B factor (or temperature factor) field of the output PDB file. Scoring schemes cannot be added to PDBALIGN without changing the source code and recompiling, and the program is similarly limited in its ability to handle residues other than the canonical 20 amino acids without manual editing of the PDB file.

In the years since the appearance of PDBALIGN, a number of other programs have been developed that permit such evaluation of sequence information in various contexts, including SwissModel [4], Chimera [5], Protein Explorer [6], ConSurf [7-10], STING [11-14], and several commercial packages. Most recently, MultiSeq has been reported [15] as an extension to the popular VMD structural analysis software [16] with an emphasis on evolutionary relationships. The underlying trend in more recent software is therefore to tie presentation of information from the MSA to a single viewing environment, such as Chimera or VMD. Such software is thus not well suited to collaborations among workers who prefer different environments and may not be well-suited to automated operation because of the emphasis on integration with a viewing and analysis package or web server largely driven by a graphical user interface (GUI). Therefore, we believe there is a niche for a flexible, up-to-date, open-source command-line tool for mapping the homology information from protein sequence alignments onto protein structures.

We have developed homolmapper as a command-line Python application for such work. Homolmapper places special emphasis on visualizing additional information such as the location of mutations, on defining and characterizing a subfamily within a given alignment, and on allowing runtime extensibility for user-defined scoring schemes and for handling of noncanonical residues in the structure and/or alignment. Homolmapper currently uses PDB files and several different MSA formats as inputs and outputs a new PDB file with homology information written to fields that do not change the spatial information. This permits the user to view the results in the viewing environment of their own choice. It is also possible to use a position-specific scoring matrix (PSSM) generated by PSI-BLAST [17] instead of an MSA. Homolmapper is small (< 250 K), portable, and requires only a working Python installation of version 2.3 or later [18].

## Implementation and results

### Rationale for program operation

Within a given PDB file, individual atoms are assigned a single ATOM or HETATM record identifying the atom and locating it within a 3-dimensional Cartesian coordinate system (see [19] for a full description of the PDB format). Within such records, individual fields such as residue number, atom name, and *z *coordinate are defined by column offset. The PDB format also provides several additional fields that provide either additional experimental detail about the structure determination (occupancy and B factor, the latter variously known as B field, B factor, and temperature factor) or provide additional descriptive information (SegID, element, and charge).

B factor has been previously used as a convenient field for storing information about homology [3]. Like B field, occupancy is a 6-column floating-point column well suited to storing such information; the other fields dispensable for unique specification of the atom name and location are smaller (SegID is 4 columns, while element and charge are each only 2 columns) and are less frequently supported by viewing programs. Homolmapper writes information to occupancy and/or B factor; additional outputs are written to the SegID field (introduced in 1996 and still in use by applications such as MODELLER, [20]), albeit with less precision. The element field can also be used. The primary output is a new PDB file to permit the user to evaluate the results in the viewing program of choice. Of course, this imposes fundamental limitations on the nature of the outputs, because they must fit the PDB standard. Homolmapper results also must be archived as complete structure files rather than simple parameter lists, although the extra storage requirements are modest and such files are by their nature stand-alone and not linked to a single "master" structure file. Homolmapper can also report scores as a tab-delimited text file for analysis with other software tools.

### Overall architecture

Homolmapper is written in Python using a functional approach and has an overall layout resembling a C program [see Additional files 1 &2]. Extensive commenting is provided within the main script for users wishing to alter the program itself, but such editing is not needed for normal operations nor for many types of user customization. Homolmapper is a strictly command-line application; user control is provided via a large number of flags that can be added to the command line to control various aspects of program behavior [see Additional file 3].

Homolmapper can also use specialized text files to import information on a number of options, such as subfamilies, mutations, non-standard residues, and additional scoring schemes. It is possible to supply some or all of the desired settings, including input files, in a text file for repetitive tasks or to supply preferred settings. This feature also aids in automation of homolmapper for batch jobs. Homolmapper can also generate such settings files, as well as generating files containing the subfamily definition, protein sequences from the input PDB file, or runtime alignment (in CLUSTAL format, [21,22]). Homolmapper output files have headers indicating their source; most provide basic information about the run parameters used in preparing the file.

### Matching the structure to the alignment

To produce meaningful output, software such as homolmapper must synchronize the protein structure and the MSA by matching the sequence contained in the protein structure to a sequence in the MSA. In homolmapper, this matching process is by default automatic. Homolmapper will first check for exact matches to the entire structure sequence (step one) and then, assuming no matches were found, for exact matches to each chain in the PDB file (step two). If no significant matches are found at this point, the sequence in each chain will be broken into short blocks, and exact matches to those blocks will be used to match the sequence (step three). Should this fail, a slower recursive algorithm will be employed (step four). If no significant matches can be found, an error will be triggered and execution will cease.

The chosen matched sequence is used to determine which portions of the structure are exact matches to the MSA; only these residues will be scored, so that the presence of exogenous sequence such as a His tag will not result in spurious results. By default, homolmapper will pass all unscored atoms to the output file without changes, but it is possible to set the output fields of unscored atoms to zero (occupancy and B factor) or to empty fields (SegID and element) if so requested.

The user has control over several aspects of this process. It is possible to choose the first matching method used, so that methods can be skipped if so chosen. It is also possible to redefine the threshold of significance for the entire matched sequence, to specify the size of the peptide blocks used at the third step, and to specify the smallest significant fragments used by the recursive algorithm. Additionally, the user can supply the name of one of the sequences in the MSA. If such a name is supplied and matches a name in the MSA, homolmapper will only test that name as a significant match. If an invalid name is supplied, homolmapper will warn the user and use the default method.

### Scoring schemes and reference sequences

Homolmapper can score sequences by gaps, insertions, identity, Shannon's entropy [23], information content, and several similarity schemes. Shannon's entropy and information content can also use user-defined amino acid sets, which readily permits correction for chemical similarity among residues in entropy scoring [24]. These schemes are by default reported in bits (i.e. taken as log base 2), but the user can choose an alternative base for the logarithm.

**Figure 1:**
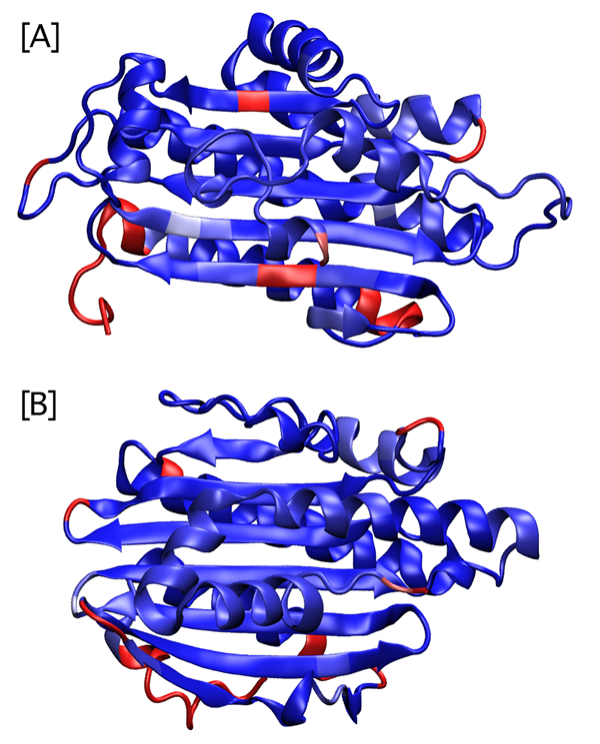
**Evaluating the fold of a homology model with homolmapper**. [A] Insertion frequency projected onto a homology model. Coproporphyrinogen oxidase (CPO) shares the same overall fold with the ferredoxin-dependent bilin reductase (FDBR) family, despite little sequence similarity [46]. A sequence alignment of PcyA, a cyanobacterial FDBR [46, 49-51], to several members of the CPO family was prepared with CLUSTAL [21, 22] and was used with the 1.58Å crystal structure of human CPO [52] to build a homology model of PcyA in MODELLER [20]. Insertion frequency in a sequence alignment of some 60 members of the FDBR family was projected onto this model structure with homolmapper and is shown colored from dark blue (no insertions) to red (insertions in 20% of sequences). Strikingly, the model predicts that two insertional hot spots fall within central β-strands (top and bottom strands). [B] Insertion frequency with the same FDBR alignment was projected onto the known crystal structure of PcyA [51] and is displayed with the same coloring conventions. The central beta sheet does not include such hot spots, demonstrating that such analysis can easily identify problematic regions in homology models for further refinement. Figure prepared with VMD [16], Stride [53], and homolmapper.

**Figure 2:**
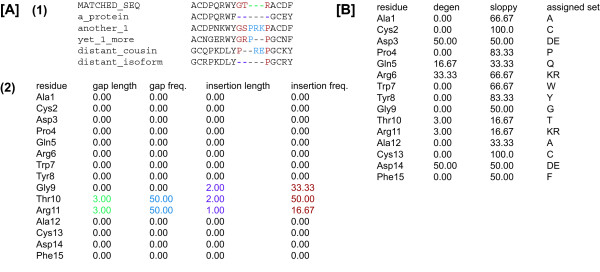
**Homolmapper scoring algorithms**. [A] Scoring of gaps and insertions. A sample alignment is shown (1), with the calculated homolmapper scores for each position shown for the available gap and insertion scoring algorithms (2), with non-zero scores highlighted by color. Gaps and insertions are always scored relative to the sequence matched to the structure (MATCHED_SEQ for this example) The positions in the alignment that give rise to those scores are shown in the same color scheme. Gap length is the length of the gap in the matched sequence relative to others (green), while gap frequency is determined by the number of sequences having any residues in the gap (blue). Insertion length is not calculated across gaps (purple). Insertion frequency is determined by sequences having residues at each position (red). [B] Scoring with user-defined amino acid sets, using the alignment from [A]. For the "degen" case, Asp and Glu are considered equivalent, and the percentage of sequences with Asp or Glu is reported for each position. For the "sloppy" example, Asp and Glu are considered equivalent members of a single set, Arg and Lys are considered equivalent members of a second set, and all other amino acids are considered to be unique members of their own sets. In this example, the alignment is scored relative to the matched sequence. The sets assigned to each amino acid in the structure are also shown.

Rather than trying to use a single objective function for scoring the MSA including gaps [25,26], homolmapper uses separate analysis of gaps and insertion. This reflects its intended uses in analyzing homology models and in structure/function studies, because gaps and insertions are useful in evaluating the overall correctness of fold in homology modeling with distant templates (Figure 1). The elongation of surface loops has also been implicated in cold adaptation of enzymes from psychrophilic organisms [27-29]. Gaps are here defined as positions where the matched sequence lacks amino acids but other proteins in the alignment have them, and insertions are defined as positions where the matched sequence has amino acids but at least one other protein lacks them. Gaps and insertions are therefore always scored relative to the matched sequence. Gaps and insertions can be scored by length or by frequency (Figure 2A).

Homolmapper offers several approaches for calculating similarity of amino acids. Identity can be calculated by a simple arithmetic procedure. This procedure compares each residue in the alignment to the aligned residue in a reference sequence, and the number of identical residues will be divided by the total number of sequences in the alignment. Alternately, the number of identical residues can be divided by the number of sequences that have a residue at the position in question to avoid penalizing for gaps, which can be useful for locating rare but conserved insertions.

**Figure 3:**
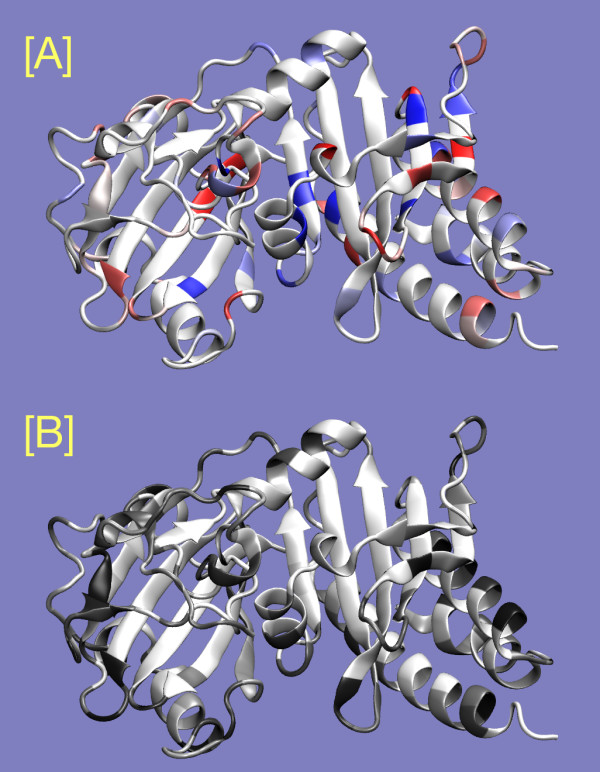
**Scoring conservation and variability**. The crystal structure of two domains of the photosensory core of DrBphP, the bacteriophytochrome from *Deinococcus radiodurans *[54], is shown colored by conservation and variability relative to a published alignment of 122 phytochromes and phytochrome-related proteins [48] using a charge scoring scheme. Conservation of net charge [A] is shown colored from red (conserved negative charge) to blue (conserved positive charge). The standard deviation of charges at each position [B] is shown colored from white (0.0) to black (0.7). The combination of the two outputs permits the user to distinguish between conserved uncharged positions (regions that are white in both panels) and variable positions with no net charge enrichment (regions that are white in [A] but black in [B]). Both panels were prepared from a single output file by coloring using values reported in occupancy [A] or B-factor [B]. Figure prepared with VMD [16], Stride [53], and homolmapper.

Several types of similarity can be calculated via a matrix lookup procedure which makes it easy to add scoring schemes by importing them as text files at runtime. BLOSUM62 [30], PAM250 [31], and charge matrices are incorporated into the homolmapper script itself. A total of 35 other scoring schemes are supplied as formatted text files as part of the homolmapper distribution, including series of BLOSUM [30], PAM [31], and Gonnet [32] substitution matrices and additional scoring matrices based on physical properties such as hydrophobicity, sidechain entropy of folding, and nonpolar accessible surface area (Table 1). Any or all of these schemes can be imported at runtime as needed, although homolmapper will use at most two such schemes because of the limited number of output fields available (occupancy and B factor). Homolmapper can report both the score (to occupancy) and the variation (to B factor) at each position for any matrix scheme, permitting assessment of both conservation and variability (Figure 3). Variation can be reported as standard deviation or standard error of the scores at each position in the MSA, or as the range of scores spanned at each position. Homolmapper also reports mean, standard deviation, and score diversity for the scores themselves in the header of the output PDB file. Score diversity is here calculated as the percentage of the maximum Shannon entropy for the score in question, and is therefore akin to the DOPS measure used in assessing alignment scoring in ScoreCONS [26]. Scoring schemes can also be reported as Z-scores relative to the calculated mean and standard deviation.

**Table 1:** Similarity scoring schemes distributed with homolmapper.

**Scheme**	**Location**	**Description**	**Reference**
BLOSUM62	built-in	BLOSUM substitution matrix	[30]
PAM250	"	PAM substitution matrix	[31]
charge	"	sidechain charge at pH 7	--
nonpolar ASA	external file	nonpolar sidechain surface area	[56]
hydrophobicity	"	ΔΔG_transfer _relative to Gly	[57, 58]
sidechain ΔS	"	sidechain entropy of folding	[59]
simple charge	"	± 1 charges (Asp, Glu, Lys, Arg)	--
identity matrix	"	for use in development	--
BLOSUM30	archive	BLOSUM substitution matrix	[30]
BLOSUM35	"	"	[30]
BLOSUM40	"	"	[30]
BLOSUM45	"	"	[30]
BLOSUM50	"	"	[30]
BLOSUM55	"	"	[30]
BLOSUM60	"	"	[30]
BLOSUM65	"	"	[30]
BLOSUM70	"	"	[30]
BLOSUM75	"	"	[30]
BLOSUM80	"	"	[30]
BLOSUM85	"	"	[30]
BLOSUM90	"	"	[30]
PAM20	"	PAM substitution matrix	[31]
PAM60	"	"	[31]
PAM120	"	"	[31]
PAM160	"	"	[31]
PAM350	"	"	[31]
Gonnet40	"	"	[32]
Gonnet80	"	Gonnet substitution matrix	[32]
Gonnet120	"	"	[32]
Gonnet160	"	"	[32]
Gonnet250	"	"	[32]
Gonnet300	"	"	[32]
Gonnet350	"	"	[32]
total ASA	"	total sidechain surface area	[56]
partial volume	"	residue partial volume in solution	[60]
volume	"	amino acid Van der Waals volume	[60]
amino acid freq.	"	amino acid frequency	[61]
exp. BLOSUM62*	"	adds standard 'B,' 'X,', 'Z'	[30]
exp. PAM250*	"	"	[31]

Three substitution matrices are built-in to the homolmapper application ("built-in"), while the others are supplied as external matrices that can be loaded at runtime if so desired. Some of the external matrices are supplied in a compressed archive ("compressed"), while others are not ("external file"). The expanded BLOSUM62 and PAM250 schemes (*) incorporate standard values for 'B,' 'X,' and 'Z' amino acids.

In addition to identity and the matrix-lookup schemes, homolmapper can score using user-defined amino acid sets. Such sets treat a group of amino acids as equivalent for scoring. Homolmapper can report the percentage of sequences having a member of such a set at each position in the structure ("degen" scoring, Figure 2B), or it can define all amino acids as belonging to sets and then report the percentage of sequences having members of the same set as a reference sequence for each position ("sloppy" scoring, Figure 2B). This feature gives the user the ability to quickly look for features such as hydrogen bond donors at an unusual pH without taking the time to create a suitable scoring matrix. It is possible to disable gap penalties for scoring by matrix-lookup and by user-defined amino acid sets.

Several of these scoring schemes are relative. For example, calculating percent identity implies the existence of a *reference sequence *to which the others will be compared. By default, homolmapper uses the *matched sequence *(effectively, the structure sequence) as the reference sequence; as discussed above, this is always the case for scoring gaps or insertions. However, schemes such as identity or similarity matrices can instead use a consensus sequence or a different sequence found in the alignment as the reference sequence. Consensus sequences can be supplied as part of the input MSA, permitting import of consensus sequences from sources such as HSSP or STING [9,12,33,34], or they can be generated by homolmapper at runtime as needed. A summary of the available reference sequence choices for different scoring schemes is presented in Table 2.

**Table 2:** Homolmapper reference sequences and scoring schemes

**Scoring Algorithm**	**Available Reference Sequences**
gaps	matched
insertions	matched
identity	matched, user-chosen, consensus
matrix-based similarity	matched, user-chosen, consensus
degen	N/A
sloppy	matched, user-chosen, consensus
entropy	N/A
information content	N/A
mutual information	N/A

The available reference sequence methods for various homolmapper scoring schemes are shown. Gaps and insertions can be scored by either length or frequency (Figure 1A), but the reference sequence is always the matched sequence. "Degen" and "sloppy" refer to different scoring algorithms using user-defined amino acid sets, as defined in the text. Reference methods are *matched *(i.e., the sequence matched to the structure), *user-chosen *(a different sequence in the MSA chosen by the user), and *consensus*. Some methods are independent of a reference sequence (N/A, not applicable).

Homolmapper can also use the SegID field of the output PDB file to report a number of additional features. The SegID field can be used to display a consensus sequence, to show which residue in the structure is assigned to which user-defined amino acid set (Figure 2B), or to show the amino acid numbers in the matched sequence corresponding to each residue in the structure. The SegID field is also used to report information about mutations, highlights, and multi-residue motifs.

### Implementation of mutations, residue highlights, and multi-residue motifs

Homolmapper can process information about mutations, reporting the results to the SegID field of the output PDB file. Mutations can be described via the command line or via an auxiliary text file supplied as an extra input. Mutations can simply be labeled on the structure, so that their probable location can be visualized to aid in interpretation, or they can be scored with a variety of algorithms. For example, mutations can be scored by conservation of the wild-type residue found in the protein in which the mutation was described, by conservation of the mutant amino acid for substitutions, or by the percentage of sequences lacking amino acids after a given position for comparison to a C-terminal truncation. Homolmapper can also score different types of mutations by different algorithms (Table 3) to provide a crude estimate of how well various mutations might be tolerated.

**Table 3:** Scoring algorithms for assessing mutations by type

**Mutation Type**	**Reported Score**
substitution	percent sequences having the mutant amino acid at the mutated position
deletion	percent sequences lacking an amino acid at the mutated position
insertion	percent sequences having an amino acid at the mutated position
N-terminal truncation	percent sequences with no amino acids at or N-terminal to the mutated position
C-terminal truncation	percent sequences with no amino acids at or C-terminal to the mutated position
frameshift	percent sequences with no amino acids at or C-terminal to the mutated position
unknown	percent sequences having the wild-type residue of the mutated protein at the mutated position

To provide a crude assessment of whether a given mutation might be tolerated, homolmapper can evaluate the alignment depending on the type of the mutation, as shown here. Scores are reported as percentages in the SegID field of the output file. The type of mutation is defined by the user as part of defining the mutation.

Homolmapper can also use the mechanisms for mutation entry to input highlights and multi-residue motifs. In highlighting, homolmapper will label the SegID field of each residue in the structure aligned with an amino acid of a given type in a different sequence in the alignment. This permits rapid visualization of where such residues would fall on the known structure, allowing assessment of potential surface accessibility, disulfide formation, or similar properties. For multi-residue motifs, the user can list allowed amino acids at each position in the motif, and homolmapper will report the percentage of sequences in the alignment that meet all criteria to the SegID field of each atom in each residue aligned with a position in the motif. If more than one motif is specified, a 1–2 character motif identifier must be supplied to allow homolmapper to track which position belongs to which motif. This motif identifier will be written to the element field of the output PDB file.

### Handling of subfamilies

Homolmapper can also compare a single subfamily to the entire alignment, reporting the results of a single scoring scheme for the subfamily (occupancy) and the entire MSA (B factor). Subfamilies can be defined by the user either explicitly (listing all members or all non-members), implicitly (by definition of amino acids that must be present or absent at certain positions for membership), by pattern-matching to sequence names (regular expressions), or by a combination of these methods. Subfamily definitions can be supplied in a text file or via the command line. It is also possible to use similar definition syntax to select a subset of the MSA for discard, permitting the user to progress from a general analysis to a more specific examination of certain sequences without having to generate a new MSA file.

In addition to permitting comparison of the subfamily to the entire MSA, homolmapper can be used to search for residues or user-defined sets that are specific to the subfamily. This is implemented by first locating positions where an allowed residue is present in a reference sequence within the subfamily, then summing the sequences within the subfamily that *lack *such an allowed residue. If the resulting sum is below a user-controlled tolerance, sequences outside of the subfamily that *possess *an allowed residue at that position are summed and compared to a second tolerance. Only positions that are below the tolerance for both tests are reported. If so requested, homolmapper can automatically search for all possible such residues and report any detected residues to SegID while leaving other outputs free for other functions. Alternately, the user can designate the occupancy and/or B-factor to search for particular residues or residue sets of interest.

This method for detecting subfamily-specific residues has the advantage that it can be applied to small subfamilies or small alignments while retaining vestigial stringency by setting the tolerances to zero. Thus, for cases in which the total sample size is too small to be statistically valid, this approach nevertheless permits rapid detection of candidate residues for further characterization, for example by site-directed mutagenesis.

### Mutual information analysis

**Figure 4:**
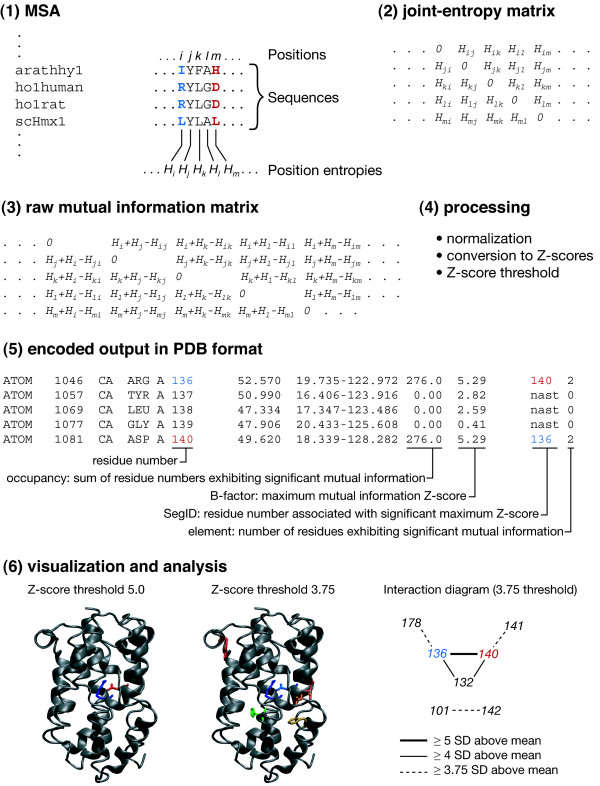
**Mutual information analysis in homolmapper**. The process used to evaluate and report mutual information in homolmapper is shown from the MSA (1) to final analysis (6) using an alignment of 75 heme oxygenases and the crystal structure of rat heme oxygenase (1DVE, [55]) for illustrative purposes. A portion of the MSA is shown in (1), with residues 136 (blue) and 140 (red) highlighted. The matched sequence is ho1rat, and the total alignment is 684 positions long. Calculation of mutual information begins with calculation of the Shannon entropies *H*_*i*_, *H*_*j*_, *H*_*k *_for all single positions *i, j, k *in the alignment [23]. Next, following the method of Gloor and co-workers [36], joint entropies *H*_*ij*_, * H*_*ik *_for all positions are calculated from the distribution of paired outcomes (2). Diagonal elements in this joint-entropy matrix are set to zero. The raw mutual information values are then calculated (3) by subtracting the joint entropy at each pair of positions from the sum of the single position entropies (*H*_*i *_+ *H*_*j *_- *H*_*ij*_), with the diagonal elements being kept at zero. Next, the raw mutual information scores can be normalized (4) by dividing by the joint entropy [36], the sum of the position entropies (redundancy), or neither. The resulting scores are converted to Z-scores (distance from the mean in standard deviations) for analysis. Maximum Z-score is reported to the B-factor field of the output PDB file for all residues (5). If this maximum Z-score is below a threshold value (by default 5, but user-controllable), a SegID of 'nast' (nothing above significance threshold) is assigned, as is seen in residues 137-139 in the example. Residues that exhibit a maximum Z-score above the cutoff value have the residue number associated with that score reported in SegID. Such residues are considered to belong to mutually informative groups, and the remaining homolmapper output fields (element and occupancy) are used to provide information about the group. The number of residues in the group is reported to element, and the sum of their residue numbers is reported to occupancy. Thus, in this example, residues 136 and 140 are mutually informative and are the only members of the group. The Z-score is reported to B-factor (5.29), and each residue has the other residue number reported to SegID. The element field for these two residues is 2, because there are two residues in the mutually informative group, and the occupancy field is 276 (= 136 + 140). This reporting scheme permits information about mutually informative positions in the alignment that fall outside of the structure to be reported nevertheless. It is also possible to punch out the final matrix of Z-scores and the normalized matrix of mutual information values for the full alignment for further analysis. The joint-entropy matrix is punched out by default to permit rapid reruns with different threshold values or different normalizations. In (6), the output PDB file is shown at a cutoff of 5 (left). Residues 136 (blue) and 140 (red) are colored by SegID and are immediately adjacent. If the threshold is lowered to 3.75 (center), additional residues are detected. The mutually informative residues in this case are colored by occupancy. By examining the significant interactions in the structure or in the text file that details all significant hits, one can construct a diagram of the interactions and their Z-scores (right). Residues 136 and 140 are part of a larger network at the lower threshold. VMD [16], Stride [53], and homolmapper were used to prepare the structural panels.

A challenge in analysis of any protein MSA is the detection of coevolving or covarying residues: while highly conserved residues are often important for protein structure or function, variable residues that interact with each other are much harder to detect yet can nonetheless be functionally or structurally important. The mutual information of pairs of positions in the MSA has been applied as a general means of analyzing such positions [35,36]. Homolmapper can calculate mutual information for an MSA by evaluating the joint entropies of all pairs of positions in the MSA and then subtracting the joint entropy from the individual position entropies (Figure 4). The resulting raw mutual-information scores can be normalized by the joint entropy [36] or by the sum of the position entropies (to yield the redundancy). Finally, scores are converted to Z-scores for further analysis.

Reporting the results of such analysis within the confines of the PDB format is difficult. We have therefore adopted a compromise scheme whereby the maximum mutual information Z-score is reported to B-factor for every residue, regardless of what that score is (Figure 4). For residues having maximum Z-scores above a threshold, the residue number associated with that score is reported to SegID. Such residues are considered to be part of a mutually informative group, and the remaining outputs (occupancy and element) are dedicated to describing that group. The number of residues in that group is reported to element, and the sum of their residue numbers (in the numbering of the MSA) is reported to occupancy. Thus, for a mutually informative pair with residue numbers 136 and 140, element would be set to 2 and occupancy would be set to 276 (Figure 4). This scheme allows residues that are part of overlapping groups to have similar occupancy values for visualization, and a full report of significant hits is also written out to a text file. The time-consuming aspect of this analysis is the calculation of the joint-entropy matrix, but homolmapper will by default punch this matrix to a text file for reuse. It is not possible to combine mutual information analysis with other scoring choices, because this scheme uses all of the current output fields.

### Using a PSSM in lieu of an MSA

Homolmapper can also take a PSSM as an input for users wishing to avoid the time involved in constructing an MSA. The PSSM parsed by homolmapper is the ASCII output PSSM generated by PSI-BLAST [17] with the "-Q" option. Scoring a PSSM will always result in the consensus sequence being reported to the element field. The SegID can be used to report the information per position or the relative weight versus pseudocounts. Occupancy and B-factor can be used for charge, degen, or sloppy scoring as in scoring an MSA, or they can be used for PSSM-specific parameters such as the PSSM value for the query sequence (Table 4).

**Table 4:** Scoring options for occupancy and B-factor fields with PSSM inputs

**Reported Score**	**Source Matrix**
log-odds of query residue	PSSM
observed percentage of query residue	observed-percentages
vector length*	PSSM
vector distance*	PSSM
vector angle*	PSSM
charge	observed-percentages
degen	observed-percentages
sloppy	observed-percentages

PSSM inputs will always result in reporting the consensus residue to the element field. The SegID field can be used to report the information per position or the relative weight versus pseudocounts. Scores marked with an asterisk (*) are calculated by considering each row in the PSSM as a 20-dimensional vector from the origin to the indicated point. The query residue is then considered as a 20-dimensional unit vector normalized to the length of the PSSM vector, and the distance and angle between the two vectors are calculated assuming the space is Euclidean.

### Handling of non-canonical residues

Many structures contain residues in addition to the canonical 20 amino acids familiar to all students of biochemistry. Such residues include amino acids such as phosphoserine [37], arising from post-translational modification, and residues such as selenomethionine [38] or diiodotyrosine [39], arising from experimental manipulations. Moreover, proteins containing selenocysteine (Cse, [40,41]) or pyrrolysine [42,43] effectively contain "extra" genetically encoded amino acids, as do proteins incorporating unnatural amino acids via modified tRNA techniques [44]. Non-standard residues may also appear in the alignment, due to sequencing ambiguity or due to the presence of noncanonical residues such as Cse.

Homolmapper is able to handle all of these cases. By default, homolmapper will recognize five easily translated residues in PDB files and translate them to their genetically encoded equivalents: phosphoserine, phosphothreonine, phosphotyrosine, hydroxyproline, and selenomethionine are translated to Ser, Thr, Tyr, Pro, and Met, respectively. Translation of other residues in PDB files is accomplished via an extra text file or command-line argument converting the three-letter residue code(s) in the PDB file to one-letter codes found in the MSA. The use of a text file for this purpose permits the user to develop a library of non-standard residues from a number of structure files for repeated reuse.

Homolmapper can also translate non-standard residues in the MSA via a command-line flag. Such residues are again translated to one of the standard one-letter codes for matrix-based scoring schemes, although no such translation is necessary for scoring gaps, insertions, or identity. This translation is handled via the command line because there is less standardization of such codes than is the case for noncanonical residues in PDB files.

Handling of residues such as Cse essentially involves expanding the set of amino acids parsed by homolmapper. This is accomplished with a separate command-line flag expanding the parsed amino acid set in combination with a text file or command-line flag equating the PDB code for the residue in question with the new one-letter code for the "extra" amino acid. There is no limit on the number of amino acids that can be added.

### User extensibility

Homolmapper offers a number of features designed to permit facile customization. Many of the more advanced features of homolmapper involve loading additional text files, so the user can readily maintain a library of frequently used accessory files, including run-settings files. As discussed, it is also possible to import similarity scoring schemes at runtime. Such schemes are normally stored as pre-formatted Python dictionaries that are loaded and compiled at runtime. The homolmapper distribution includes a small utility that generates matrices in this format from user-supplied text files describing per-residue properties, making user generation of new schemes much faster and more reliable. Homolmapper can also import these schemes as plain text files.

Homolmapper is controlled by flags specified on the command line. Such an interface normally requires the user to memorize the names of the flags that are relevant to their own work. However, homolmapper permits the user to supply synonyms that can then be redefined by the application itself, so that the user can rename flags should they find their names difficult to remember. Such redefinitions can be done on the command line or via an accessory text file. This file can then be referenced in a run-settings file for repeated reuse, permitting the user to use their own mnemonics.

### Documentation

Homolmapper is distributed with examples of all the accessory input files [see Additional files 1 &2]. Each of these examples is heavily commented to aid in understanding the uses and formatting for the various files. Homolmapper also can generate considerable help information at runtime, via flags such as – help or – files. The homolmapper script itself is heavily commented, although these comments are intended to aid in programming rather than use. Several small utilities are also included in this distribution.

In addition to the documentation distributed with homolmapper itself, a User Guide is available as a separate download [see Additional file 3]. This PDF document provides demonstrations of many aspects of homolmapper operation with included structures and alignments, including both basic operations and more advanced applications such as working with expanded amino acid sets or mutual information analysis. All required files are included with this distribution.

## Discussion

### Planned future development

Development of homolmapper is an ongoing process. Improvements are planned in several aspects of program operation, including additional scoring schemes, acceptance of additional file formats for the MSA, handling of multiple structural inputs, and greater flexibility in handling non-standard PDB files and in scoring PSSM inputs. The open architecture of the Python implementation also facilitates distribution of user-suggested improvements to a wider user community. Techniques for comparison of multiple structures and subfamilies will eventually be incorporated as long-term improvements, and a dedicated GUI is ultimately planned.

### Intended uses

Homolmapper is well suited to general visualization of homology relationships, particularly in collaborative environments where different workers are using different molecular viewing programs. It is also intended to aid in evaluation of candidate homology models, in visualizing the locations of mutations or motifs, and in comparison of a subfamily to an entire MSA, including location of subfamily-specific residues. Homolmapper is thus a useful addition to the range of software permitting visualization of homology relationships in terms of protein structure.

## Conclusion

Earlier, partially functional versions of homolmapper have already proven useful for evaluating homology models [45,46] and for examining homology and mutational information in a structural context [47,48]. Homolmapper is designed to permit some types of user customization with minimal effort and no programming. Homolmapper is distributed under a modified BSD to permit interested users to work with the program itself as desired. It is particularly well suited to collaborations involving different molecular viewing environments. Extensive documentation is available, including many examples, and the homolmapper script itself is small and portable. We anticipate that this application will prove a useful tool for workers investigating structure and function, structural modeling, and other fields suited to evaluation of protein sequence homology in a structural context.

## Availability and requirements

• **Project name: **homolmapper

• **Project home page: **homolmapper home page at the Lagarias lab site, URL:  (point-and-click license agreement with no registration; documentation with examples available at same URL). Also supplied with this manuscript as additional files.

• **Operating system(s): **Platform independent

• **Programming language: **Python

• **Other requirements: **Python 2.3 or higher; standard Python modules (sys, time, and os including the os.path submodule); Windows versions require the win32 extensions to Python (sourceforge.net/projects/pywin32) for automated loading of scoring schemes.

• **License: **based on University of California BSD

• **Any restrictions to use by non-academics: **yes

## List of abbreviations

Cse, selenocysteine

GUI, Graphical User Interface

MSA, multiple (protein) sequence alignment

PDB file, Protein Data Bank file format for protein structures [19]

PSSM, position-specific scoring matrix

## Authors' contributions

NCR developed the software and wrote the documentation. JCL aided in development and in drafting the manuscript. Both authors read and approved the final manuscript.

## Supplementary Material

Additional file 1 The standard homolmapper distribution (current as of March 2007), containing homolmapper itself and associated utilities and accessory files. Click here for file

Additional file 2 Alternative homolmapper distribution for operation as a batch file under Windows, current as of March 2007. Click here for file

Additional file 3 Additional documentation for homolmapper, including the PDF User Guide and example structure, alignment, and accessory files, current as of March 2007. Click here for file
