# Supplementary material for: Flexible mapping of homology onto structure with Homolmapper
Source: BMC Bioinformatics. 2007 Apr 11;8:123. doi: 10.1186/1471-2105-8-123 (PMC1955750; doi:10.1186/1471-2105-8-123)
Supplement: Additional file 3 — Additional documentation for homolmapper, including the PDF User Guide and example structure, alignment, and accessory files, current as of March 2007. [file 1471-2105-8-123-S3.gz › user_guide/UserGuide.16.7.pdf]

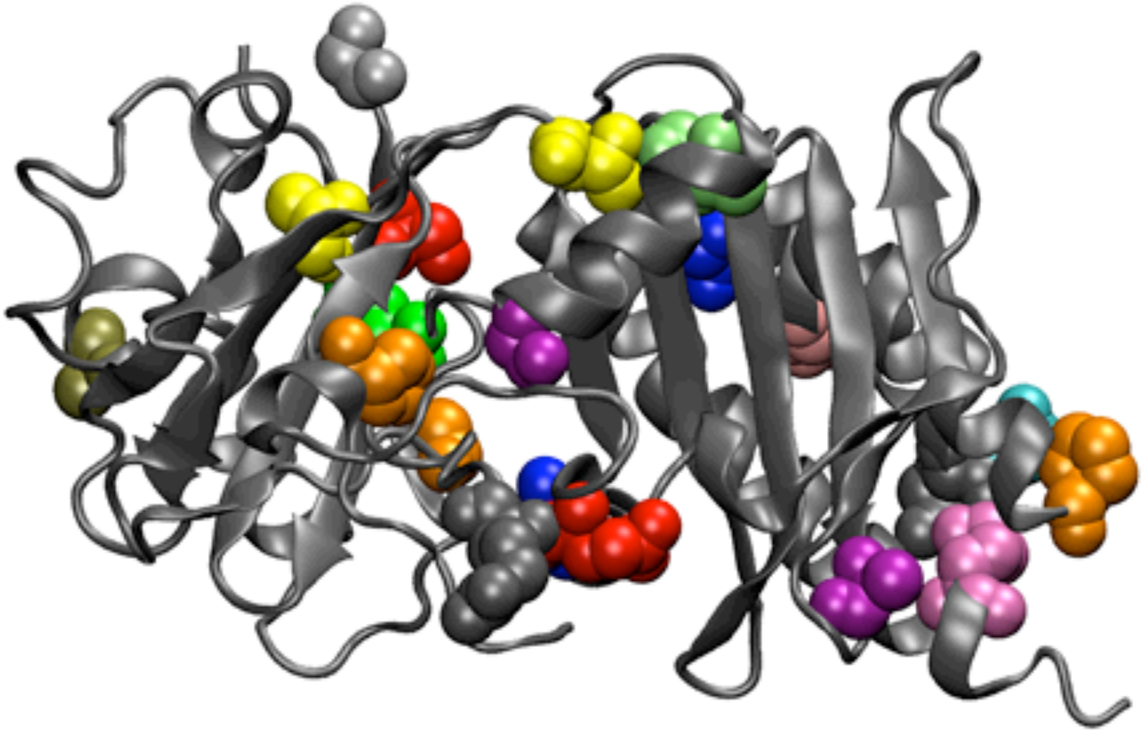

HOMOLMAPPER v16.7

User Guide

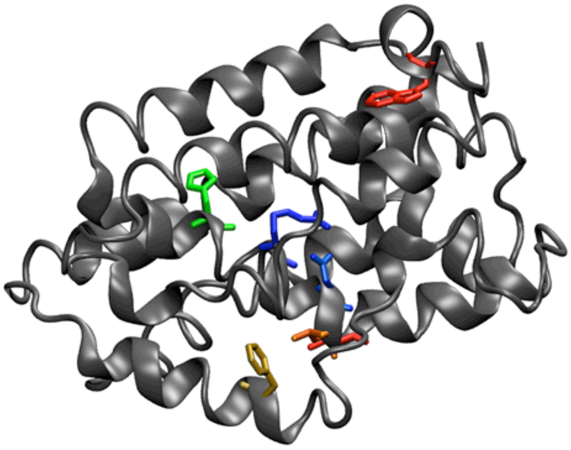

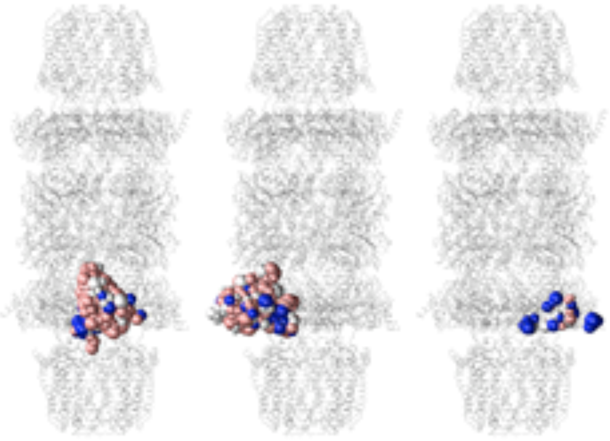

Copyright © 2006-2007 The Regents of the University of California
All Rights Reserved

Chapter 1. Installation

1.1 Definitions

Chapter 2. Core operations

2.1 The basics

2.2 Choosing scoring options

2.3 Reference sequences for scoring

2.4 Matching structure to sequence

2.5 Alignment formats

Chapter 3. Beyond B factor

3.1 Basic uses for the SegID field

3.2 Scoring with user-defined residue sets

3.3 Mutations

3.4 Highlights

3.5 Multi-residue motifs

3.6 Using a PSSM instead of an alignment

3.7 Mutual-information analysis

Chapter 4. Analyzing a subfamily

4.1 Defining a subfamily by name (explicit definition)

4.2 Defining a subfamily by sequence (implicit definition)

4.3 Defining a subfamily by patterns in the names (regex definition)

4.4 Reference sequences and subfamilies

4.5 Analyzing subfamily-specific residues

Chapter 5. Scoring the unscored

5.1 Handling unscored residues

5.2 Handling nonstandard residues in the structure

5.3 Handling nonstandard residues in the alignment

5.4 Handling a 21st amino acid

Chapter 6. Pushing the boundaries

6.1 Creating a new scoring scheme

6.2 Customizing flags and scoring options

6.3 Pruning the alignment

6.4 Automation and scripting

Chapter 7. Under the hood

7.1 Homolmapper headers

7.2 Identity identified

7.3 Normalization and correction

7.4 Troubleshooting

Chapter 1: Installation and introduction.

About homolmapper and the user guide: This document illustrates the usage of homolmapper, a command-line Python application that can map the information from protein sequence alignments onto protein structure files. This guide is written in a tutorial form and assumes the availability of the tutorial files, which are distributed with the User Guide. The tutorial files include both structures and alignments. The alignments provided with the user guide are purely for illustrative purposes; they are not intended to be significant demonstrations of protein homology. The starting structures all have names beginning with 1 or 2, while none of the outputs from this demo will have such names. These structures have also been chosen solely to illustrate aspects of program function; these demo settings will not suffice for people with serious interest in these structures.

Each section refers to a specific group of these files in discussing various aspects of homolmapper operation. Each section also refers to help flags that can be used to get a terse summary of the various aspects of operation relevant to that section ("homolmapper --help" lists these help flags). A working knowledge of unix (or the local command-line interface) is highly recommended. People who have successfully installed homolmapper and wish to jump straight to examples will find them beginning with Chapter 2.

Requirements: Homolmapper requires a functional Python executable of Version 2.3 or later. On most platforms, typing "python -V" will report the version number (e.g., "Python 2.3" appears on the screen; lower case 'v' will work but will launch the interactive mode,

which can be exited with "CTRL-D" or the local equivalent, such as "CTRL-Z" followed by "RETURN"). If this instead gives an error message or an earlier version, installing Python (www.python.org) is necessary. To look at the results, a structure viewer that can display the information in occupancy, B factor is required. The ability to display the information in SegID and element is also useful. Pictures for this guide were prepared with VMD (Humphrey *et al., J. Mol. Graphics,* **14:** 33; available at www.ks.uiuc.edu/Research/vmd), which can handle all four of these fields and more. VMD itself uses STRIDE (Frishman & Argos, *Proteins* **23:** 566) to calculate secondary structure.

Installation for Mac, unix, etc: HomolMapper can be installed such that it will run as an executable command like "ls" or other unix commands. To do this, there are two main approaches. Both are described here:

1. Ensure that there is a functioning Python present ("python -V" or "which python" should confirm its presence; the former will also indicate whether it is the required version 2.3 or later). If Python is absent or outdated, it can be found at www.python.org.

2. Edit the first line of homolmapper (the shebang line) to point to the Python executable. The default setting is correct for the built-in framework Python under Mac OS 10.3 and 10.4 (/usr/bin/python). This should be done in a text editor such as emacs or vi, *not* with a word processor.

3. Make homolmapper executable (chmod 755 homolmapper).

A. Now, *if you have permission to copy files to directories in the path*, you can follow these steps to place homolmapper in the path:

4. Copy homolmapper to a directory located within the path. The path is defined by the current value of the PATH environment variable (examined with "echo $PATH"). The actual copy command may be:

`cp homolmapper /usr/local/bin/` (assuming /usr/local/bin/ exists & is in the path)

or, on Macs, will probably need to be:

`sudo cp homolmapper /usr/local/bin` (same assumptions)

The latter syntax will require a password. If the first syntax gives permissions problems, the second may work. Mac users will note that /usr/local/bin may not be there; if it isn't, it will need to be created (sudo mkdir /usr/local/bin) and added to the path, as described below. Alternately, one can follow the directions below (at **B**).

5. Now, set the original homolmapper back to a non-executable file (chmod 660 homolmapper). This is more in the nature of good practice than required.

B. Alternately, *if you lack those permissions or prefer not to tinker with stuff in the general system folders,* you can follow these steps to add the homolmapper directory to the path:

4. Put homolmapper where you want it to be (probably not the Desktop, for Mac people).

5. Edit your shell preferences file to add that directory to your path. For example, under tcsh, one would add a line like this to the hidden .tcshrc file *with a text editor* to make /usr/local/bin/ part of the path:

```
set path = ( $path /usr/local/bin )
```

The $path part is essential. This change will not take place until a new window is opened or the initialization file is otherwise run.

Installation for Windows: The absence of a shebang syntax (#! on the first line) makes this trickier. As the matter currently stands, there is a separate release for Win32 systems. This release (w32_hm.tar.gz) includes Windows versions of the various scripts present in the homolmapper distribution as batch files (.bat extension). Windows also does not normally ship with a functioning Python, making for a longer installation process. Some versions of Windows also have problems with pipes, which are used by the --lib= and --lib flags; a Windows-specific version of Python can solve these problems.

1. If it is not present or is outdated, install Python (www.python.org). This installation is quite straightforward. To get a working version of the --lib and --lib= flags (which permit the user to load all scoring schemes from a given directory), an additional installation is required; see sourceforge.net/projects/pywin32 for details. These flags are little used; in fact, they are not used at all in any of the examples in the User Guide. We therefore do

not recommend the additional work unless you have considerable expertise in working "under the hood" with the operating system.

2. Once python is installed, you will need to add the python folder to the path. Right-clicking on "My Computer" and then selecting "Properties" will give a window with an "Advanced" button. This button leads to the ability to examine and change the "Environment variables" (which is what is needed). The PATH environment variable will need to have the Python folder added to the end of the current list. If you have privileges to change the value in the bottom pane, it needs to go on the end as:

`(stuff);C:\Python25\`   (or the appropriate location)

Alternately, you may need to create a "New" local environment variable (top pane) which would then contain something like this:

`C:\Python25\`   (again, make it the right spot)

3. Once this is successful, you will be able to tell by the following steps:

a. run cmd.exe, *NOT* command.com! cmd.exe is chosen by Start>Run, followed by entering "cmd" at the prompt and hitting return.

b. at the resulting prompt, type "python -V" and see what happens. Success is indicated by a short message like "Python 2.5" or similar. Any type of error message is bad, and using "-v" instead of "-V" will launch an interactive mode which can be exited by typing "exit" and following the directions.

4. unpack the w32_hm.tar.gz archive using WINZIP or similar and move the w32_hm folder to a good spot (e.g., in "C:\Documents and Settings\YOUR_FOLDER").

5. Add this location to the PATH environment variable, as for Python above. For example,

```
(optional stuff);C:\Python25\;C:Documents and Settings\YOUR_FOLDER\w32_hm\
```

This keeps Python in the path and adds the homolmapper folder. Note the ';' between the two settings, which is required.

6. If you want any of the accessory scripts in utils/scripts to run from the command line as simple executable commands, it will be easiest to copy them into the homolmapper main directory. From within the homolmapper directory, one would type:

`copy utils/scripts/*.bat .` (the '.' at the end is required)

This is not a required step, but the linecomp utility is useful in working through the demo.

7. For *each* of the .bat files in the homolmapper directory, you will need to edit the explicit path right at the beginning of the script to point to homolmapper. In theory, this is not necessary, but in practice it seems to be much more reliable. It is preferable to use a text editor for this. Lines 4 and 7 of the homolmapper.bat file look like this:

```
python -x "C:\Documents and Settings\ncrockwell\w32_hm\%0".bat %1 %2 %3 %4 %5

python -x "C:\Documents and Settings\ncrockwell\w32_hm\%0".bat %*
```

They need to be changed to point to your local w32_hm folder. For example, one could edit the second line to look like this:

```
python -x "C:\Documents and Settings\YOUR_FOLDER\w32_hm\%0".bat %*
```

The reason there are two lines is that pre-Windows NT does not accept “%*” as a shorthand for all of the command-line parameters, which is why they are listed out in the first line. This older syntax limits the number of parameters that can be passed, so homolmapper will be much more flexible on NT. It is only necessary to edit the appropriate one for the local version of Windows. If you are unsure which is appropriate, editing both will not cause problems.

8. Now test for success: move to a different directory, like the desktop, and run homolmapper by typing “homolmapper” by itself. The result should look like this:

```
        no structure supplied.
        no alignment supplied.

        homolmapper version 16.7.4

['blosum62', 'pam250', 'charge']
```

If it does, the hard part is past and homolmapper is ready to run. If a warning about pipes appears, the --lib and --lib= flags will not work, as discussed above. This is not a problem

for a beginning user and is unlikely to be a problem for anything other than automated, high-throughput applications.

Updating homolmapper: If you have an old version and wish to replace it with a new version, simply make all necessary edits to the new version (including permissions changes, path changes, etc.) and copy it onto the old one. It is not necessary to remove the old one. The same applies to accessory scripts associated with homolmapper.

Alternative installation: One can also simply unpack homolmapper and use it as is. In this case, it is easiest to work in the directory containing homolmapper. One would place the structure and alignment files in the homolmapper directory and then run homolmapper as "python homolmapper" followed by the various arguments to pass to homolmapper. One could also simply keep moving homolmapper around to the directories containing the structure and alignment files.

What homolmapper does (the short version): Homolmapper is a command-line application. Given a protein structure in PDB format (www.rcsb.org) and a protein sequence alignment in CLUSTAL format (Thompson *et al., Nucleic Acids Research* **22:** 4673) or several others, homolmapper will match the structure to a sequence in the alignment (assuming there is a good match) and will report various information from the alignment in a new PDB file. That new file can then be examined to visualize the homology relationships. Homolmapper is currently a strict command-line application whose behavior is controlled by typing in settings and filenames.

1.1 Definitions

The following definitions may aid in navigating this document and in using homolmapper. They should definitely be consulted prior to attempting any reprogramming.

gap A gap is a position in the sequence alignment where the sequence matched to the structure lacks an amino acid but at least one other sequence in the alignment has one.

insertion An insertion is a position in the sequence alignment where the sequence matched to the structure has an amino acid but at least one other sequence in the alignment lacks one.

matrix Similarity or substitution matrices assign values to measure conservation among the different amino acids; substitution matrices such as the widely used BLOSUM or PAM series base those values on calculated substitution frequencies, while other matrices may derive values from the physical properties of the amino acids.

subfamily The alignment may contain one or more subsets of proteins that are more closely related to each other than to the rest of the alignment. These subsets are subfamilies. Homolmapper can analyze one user-defined subfamily relative to the rest of the alignment, including the detection of residues specific to the subfamily.

occupancy A field in the PDB format, normally used to report how populated that atom is in the crystal structure determination. One of the two fields homolmapper uses to report most results.

B factor Also called B field or temperature factor, this is the other PDB field homolmapper uses for most results. Normally used as a measure of how ordered (i.e. immobile) a given atom is in the crystal.

SegID A newer and somewhat controversial field in the PDB, used by homolmapper to report additional information. This field is smaller than occupancy and B factor and is the destination for scoring mutations and most other "extra" scoring information.

element Another field introduced in the mid-90's, element is a two-column field intended for storing the element of each atom. Homolmapper uses this field when handling multiple motifs, when using a PSSM instead of an MSA (Chapter 3), and in mutual-information analysis.

stdout The "standard output" stream in unix and related operating systems. Used by homolmapper to report progress and warnings.

stderr The “standard error” stream (cf. stdout above). Used by homolmapper to report fatal errors. In most cases, both stdout and stderr will print to the screen. Redirecting output with the ‘> NAME’ character will send stdout to file NAME without redirecting stderr.

accessory Homolmapper uses a PDB file (whose name ends in .pdb) and a multiple sequence alignment (MSA, with a name ending in a supported extension) as minimal inputs. Some features may use additional text files, considered accessory files.

flag Homolmapper can read in settings (flags) from the command line. Filenames are all recognized by extension (.pdb, .aln, and so forth), while flags are indicated by a double dash at the start (e.g., --help).

column The PDB format defines proteins as a series of records, or lines of text. Each line contains certain information in certain columns. For example, the first 6 columns indicate what type of record each line is (“REMARK” versus “HETATM” and so forth). Were one to forget the two spaces needed to pad the right to reach six characters in an ATOM line, the column offsets would be shifted. Homolmapper uses those column offsets to read in (parse) the .pdb file, so such a shift will cause the file to be misunderstood.

whitespace Space characters, tabs, etc.: the “white space” between letters printed on a page of white paper. CLUSTAL alignment files separate the sequence name from each block of sequence with whitespace. Alignment and accessory files are parsed by whitespace.

comment Any text intended to make life easier for the user or developer but not processed by the computer is a comment. HomoImapper considers a line of text starting with a ‘#’ character to be a comment.

match HomoImapper must decide what part of the alignment corresponds to what part of the structure and therefore must decide which sequence in the alignment is the best match for the structure. The chosen sequence is the *matched sequence*.

reference Many scoring schemes are relative; for example, for there to be a gap in an alignment there must be a sequence that doesn’t have that gap. Similarly, percent identity implies the existence of some sequence to which others may be identical. HomoImapper considers this “anchor” to be the *reference sequence.*

MSA A multiple sequence alignment of protein sequences. The supported formats are discussed in section 2.5.

Chapter 2: Core operations

Overview: This chapter illustrates basic use of homolmapper: running the application, specifying input files, and choosing scoring options. It also covers two other topics: choosing a reference sequence and controlling the choice of a matched sequence.

2.1 The basics

Example directory: ubiquitin
Help flags: --help, --files, --flags

Background: To illustrate working with homolmapper, the tutorial contains several directories. Each directory contains at least one PDB file and at least one sequence alignment, along with accessory files. In particular, each directory contains several files with the .hmset extension. These are homolmapper settings files. They contain various flags and filenames that will drive a homolmapper run for one of the various examples. The advantage of this is that the user is spared having to type all of the various flags in. As text files, they can be read and edited; this is a good way to expand on the tutorial.

Examples:

1. In the ubiquitin directory of the tutorial, there is a file called default.hmset. Running this settings file illustrates a simple run using the default settings. Using this file is simple enough: homolmapper default.hmset (or python homolmapper default.hmset) is all the

command line that is required. Execution results in a small amount of information appearing on the screen:

```
Chosen sequence (amino acid overlap)
1UBQ ( 76 )

Calculating gap scores by length.
Calculating percent identity.

Complete.
```

A new pdb file is created in the current directory. Here are equivalent views of the original PDB file (1UBQ.pdb) and the output file (default.pdb; this is not a stereo diagram!). On the left, the experimental B factors were used to color the structure, with blue as a low value (ordered) and red as a high value (disordered). Most of the structure is quite close in B factor, with only the flexible C terminus exhibiting substantially higher values. On the right, the homolmapper output is colored by the new B factor values, which now are percent identity across the alignment file ubiqs.aln. Values range from 0 (variable, red) to 100 (conserved, blue).

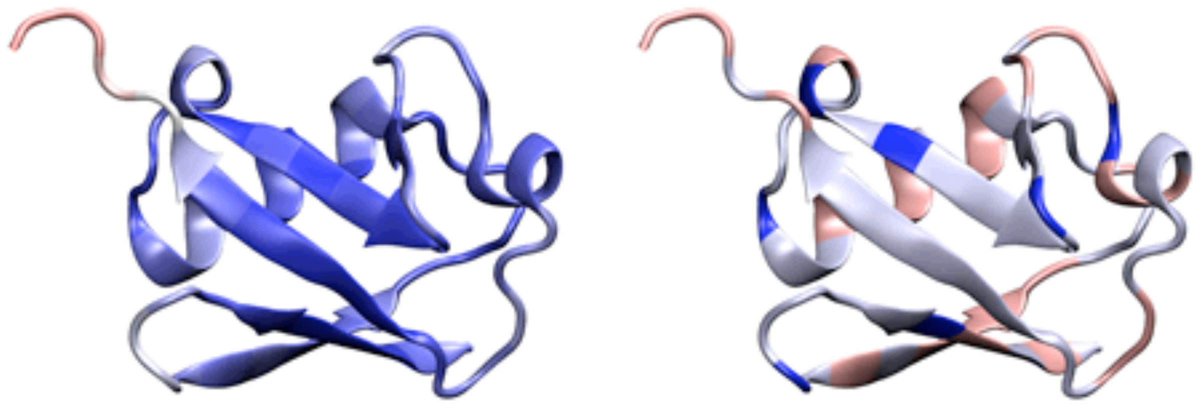

As a further illustration of homolmapper operation, here are the first five alpha carbons of the input PDB file (1UBQ.pdb):

```
                                                  occupancy _____
ATOM      2  CA  MET     1      26.266  25.413   2.842  1.00 10.38      1UBQ  72
ATOM     10  CA  GLN     2      26.850  29.021   3.898  1.00  9.07      1UBQ  80
ATOM     19  CA  ILE     3      26.235  30.058   7.497  1.00  5.07      1UBQ  89
ATOM     27  CA  PHE     4      26.772  33.436   9.197  1.00  4.68      1UBQ  97
ATOM     38  CA  VAL     5      28.605  33.965  12.503  1.00  3.87      1UBQ 108
                                                         B factor _____
```

For comparison, here are the same atoms for the output file. Such comparisons are used extensively throughout this tutorial, because it is easier to see exactly what the program is doing by looking at the actual values, even though most of the scientific content is best understood by visualization.

```
                                                  occupancy _____
ATOM      2  CA  MET     1      26.266  25.413   2.842  1.00 66.67      1UBQ  72
ATOM     10  CA  GLN     2      26.850  29.021   3.898  0.00 33.33      1UBQ  80
ATOM     19  CA  ILE     3      26.235  30.058   7.497  0.00 66.67      1UBQ  89
ATOM     27  CA  PHE     4      26.772  33.436   9.197  0.00 33.33      1UBQ  97
ATOM     38  CA  VAL     5      28.605  33.965  12.503  0.00 100.0      1UBQ 108
                                                         B factor _____
```

The original B factor values have been replaced by values reflecting percent identity in the alignment. The occupancy values are also now different. They now are scored by the maximum length of gaps at each position. Gaps and insertions can be scored by how long they are (by length, as is the case here) or by how often they occur (by frequency, which is the default for insertions).

Here is the alignment used in scoring. Out of the first five residues of 1UBQ, only the fifth is absolutely conserved (Val5). The score for Phe4 is only 33% even though two out of three residues are identical, because percent identity is scored relative to the structure

sequence in this example. N-terminal to the first residue, there is a one-residue gap in the structure sequence. The sequence at the C-terminus is longer in the structure sequence than in the other sequences, which constitutes an insertion and not a gap under the homolmapper definitions (discussed in the introduction). Were one to use one of the other structure files instead of 1UQB (all three in the alignment are present), the output would have nonzero gap scores at the C-terminus but not the N-terminus.

```
1UBQ            -MQIFVKTLTGKTITLEVEPSDTIENVKAKIQDKEGIPPDQQRLIFAGKQLEDGRTLSDY
1BT0            -MLIKVKTLTGKEIEIDIEPTDTIDRIKERVEEKEGIPPVQQRLIYAGKQLADDKTAKDY
1WM3            HINLKVAGQDGSVVQFKIKRHTPLSKLMKAYCERQGLSMRQIRFRFDGQPINETDTPAQL
                 : : *    *. : :.::   .:..:     :::*:.  * *: : *: : :  *  :

1UBQ            NIQKESTLHLVLRLRGG
1BT0            NIEGGSVLHLVLAL---
1WM3            EMEDEDTIDVFQ-----
                :::  ..:.:.
```

This examination of the input and output files begs the question of what happened in the middle bit. For this example, the settings are all contained in default.hmset. Default.hmset starts with several comment lines (each starting with a ‘#’ character). The relevant contents of default.hmset are three lines as follows:

```
1UBQ.pdb
ubiqs.aln
--out=default
```

It is not necessary to have each setting on its own line (with one exception described in chapter 7), though it can make for easier reading and editing. These settings are equivalent to the following command line:

```
homolmapper 1UBQ.pdb ubiqs.aln --out=default
```

This simply gives the filenames and sets a single flag: --out=default. The --out= flag is used to specify the name of the output files (both the output PDB file and any accessory files that might be written). Adding --out=fred to the default.hmset command line (or editing the .hmset file to set --out=fred) would give an output file named fred.pdb instead. Settings from .hmset files will be overridden by any setting supplied on the command line itself. Therefore, one can use “homolmapper default.hmset 1WM3.pdb” to use these settings with a different PDB file.

2. Homolmapper offers a number of ways to score the alignment. As a quick example, pam250.hmset performs an identical run to default.hmset except that the PAM250 substitution matrix introduced by Dayhoff and co-workers is used for B factor rather than percent identity. This example also runs with more information written to stdout (higher verbosity) because of the --verbose=2 flag. Verbosity is controlled by --verbose=X, where X is an integer between 0 and 4 and the default value is 1. At --verbose=3, additional information including timing is printed out, while --verbose=4 generates still more information and --verbose=5 generates additional accessory files as well. Timing information can be requested less noisily by using the --timing flag. Here are PAM250 scores for the first five alpha carbons:

```
ATOM      2  CA  MET     1      26.266  25.413   2.842  1.00 77.78      1UBQ  72
ATOM     10  CA  GLN     2      26.850  29.021   3.898  0.00 25.00      1UBQ  80
ATOM     19  CA  ILE     3      26.235  30.058   7.497  0.00 80.00      1UBQ  89
ATOM     27  CA  PHE     4      26.772  33.436   9.197  0.00 -3.70      1UBQ  97
ATOM     38  CA  VAL     5      28.605  33.965  12.503  0.00 100.0      1UBQ 108
```

The occupancy is the same as the default run, as expected given that the scoring scheme is identical. The B factor scores are now different. The PAM250 scoring scheme

in homolmapper is normalized, such that the maximum score is 100 (given to the absolutely conserved Val5). Here are views of identity (left, from default.hmset) and PAM250 (right, from pam250.hmset) colored from red (variable) to blue (conserved):

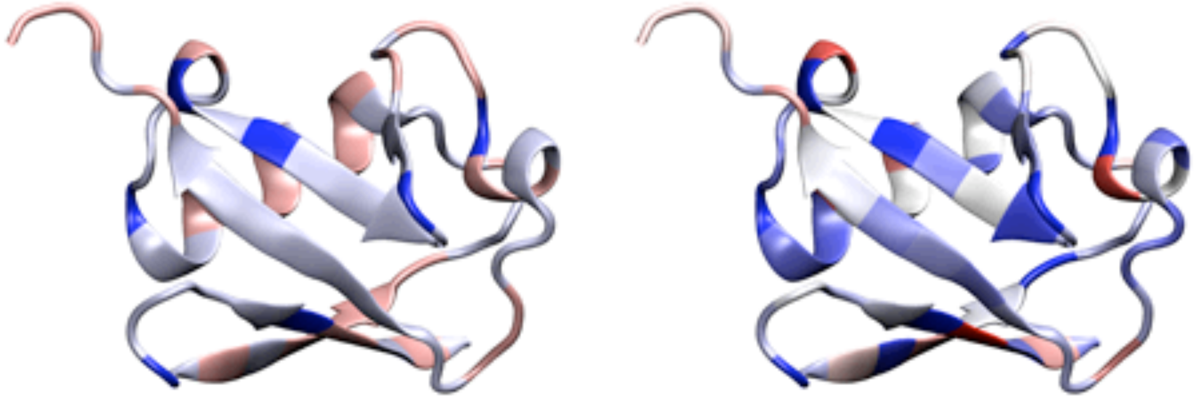

Comparing default.pdb and pam250.pdb visually is informative, but detailed analysis can sometimes necessitate looking at the scores quantitatively. Doing that by hand is tedious in the extreme. Text utilities can aid in this process; for example, unix users could compare files more rapidly with diff. The homolmapper distribution contains several small utilities which can aid in working with homolmapper or its input files (these utilities are all written in Python; installation and operation is therefore similar to that for homolmapper itself). One of these, linecomp, can be used to compare text files. For linecomp, files must be the same length (unlike diff), and more than two files can be compared at a time (again unlike diff). Default output is the number of lines that differ between each pair of files, but the differing lines are displayed by running linecomp with the --verbose flag. Homolmapper runs will almost always vary by at least 1 line because of a timestamp in the header. Using linecomp on default.pdb and pam250.pdb indicates that 419 lines differ between the 2 files. Here are the two lines in the pam250.pdb header that describe scoring choices:

```
REMARK  occupancy: gap  len           structure
REMARK  B-factor: sim   pam250        structure
```

Comparison with the equivalent lines in the default.pdb header shows that “ID” has been replaced with “pam250” to indicate the scoring scheme.

3. The above examples have made no mention of the quality of the alignment. Homolmapper does not evaluate the input MSA extensively; it is assumed that the alignment is at least good enough to be worth examining in a structural context. As an example of this point, botch.hmset uses the default settings with a manually garbled alignment, shown here:

```
1UBQ      -MQIFVKTLTGKTITLEVEPSDTIENVKAKIQDKEGIPPDQQRLIFAGKQLEDGRTLSDY------
1BT0      ----MLIKVKTLTG---KEIEIDIEPTDTIDRIKERVEEKEGIPPVQQRLIYAGKQLADDKTAKDY
1WM3      ---HINLKVAGQDGSVVQFKIKRHTPLSKLMKAYCERQGLSMRQIRFRFDGQPINETDTPAQL---

1UBQ        NIQKESTLHLVLRLRGG----------------------------------
1BT0        -----------------NIEGGSVLHLVLAL--------------------
1WM3        ----------------------------------EMEDEDTIDVFQ-----
```

Here are pictures of the correct alignment (left) and the incorrect one (right):

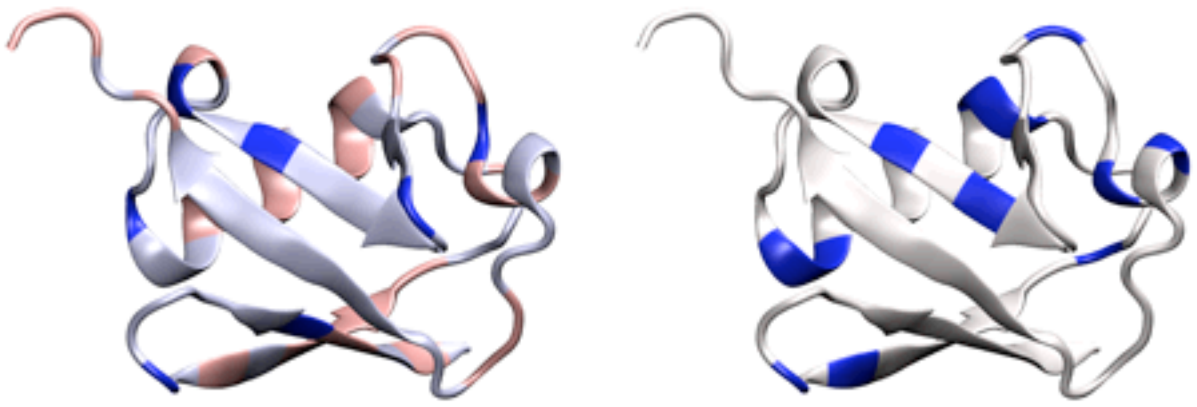

A valid output file is still generated, although the scores are obviously quite different (he resulting .pdb file differs from the correct file in 367 lines). Here are the first five alpha carbons:

```
ATOM      2  CA  MET     1      26.266  25.413   2.842  1.00 33.33      1UBQ  72
ATOM     10  CA  GLN     2      26.850  29.021   3.898  0.00 33.33      1UBQ  80
ATOM     19  CA  ILE     3      26.235  30.058   7.497  0.00 33.33      1UBQ  89
ATOM     27  CA  PHE     4      26.772  33.436   9.197  0.00 33.33      1UBQ  97
ATOM     38  CA  VAL     5      28.605  33.965  12.503  0.00 33.33      1UBQ 108
```

The identity scores have all changed due to the arbitrary changes in the alignment.

Additional information:

1. The default output filename is based on the input filename. By default, 1UBQ.pdb would give rise to 1UBQ.map.pdb. If one wanted to generate 1UBQ.fred.pdb instead, one could use the --out= flag (--out=1UBQ.fred) or another flag, --ext=, which changes the extension (--ext=.fred; note that the initial '.' is necessary if it is desired).

2. Both occupancy and B factor are per-atom properties; values are assigned to each atom in the structure rather than to each residue. Homolmapper scores are per-residue properties: occupancy and B factor are the same for every atom in a given residue.

3. Homolmapper output files will contain all "ATOM " and "HETATM" lines in the input PDB file. Proper "TER   " lines (with 3 spaces after the TER) will be included as well., as will proper "MODEL " and "ENDMDL" lines. Other lines will be lost, so it is not a good idea to overwrite the starting PDB file.

4. By default, homolmapper will not overwrite a file. Thus, repeating a run exactly without either renaming or deleting the output file from the first run will trigger an error. This behavior can be suppressed with the --overwrite or --setup flags.

5. Identity scoring (and similarity schemes such as PAM250) are by default normalized for the total number of sequences in the alignment, so that positions that fall in a highly conserved but rare insertion will get low scores. This behavior can be suppressed by running homolmapper with the --no_gap_penalty flag.

2.2 Choosing scoring options

Example directory: phytochrome
Help flags: --scoring, --files

Background: The examples so far have used only a few of the many possible options. For instance, one could want to examine how often gaps occur in the alignment (score gaps by frequency), or use matrices based on physical properties such as charge. Homolmapper provides the means to do all of this; the limitation is that there are only two fields used to report scores (occupancy and B field), so each run can only report one or two scores at a time. The scoring schemes used for each run are written to the header of the output files.

The --occ= and --B= flags: Scoring is controlled with these two flags; --occ= tells homolmapper what score to write to occupancy, and --B= indicates what score should be written to B factor. The default values are --occ=gap.len (score gaps by length to occupancy) and --B=sim.ID.structure (report similarity as percent identity relative to the structure sequence in B factor). In the ubiquitin example using PAM250, the occupancy setting was identical, but the B factor was changed to --B=sim.pam250.structure. The

basic syntax is to list a *class* of scoring (gap, ins or sim for gap, insertion, or similarity) followed by a *type* of scoring. The types of scoring are dependent on what class is being used; scoring gaps by charge or identity is nonsensical, while scoring similarity by frequency is ambiguous. One or both of these flags can be supplied; it is thus possible to retain the experimental occupancy or B factor values if so desired, although only a single scoring scheme can be applied in such a case.

Scoring by gaps or insertions: If the scoring class is 'gap' or 'ins' (gaps and insertions), the allowed types are 'len' and 'freq' (length and frequency, but shorter to type). Other types will trigger errors if meaningful for the 'sim' class or will be ignored to give default types. The default type for gap scoring is length (gap.len), and the default type for insertion scoring is frequency (ins.freq).

Scoring by similarity or identity: For the 'sim' scoring class, the allowed types are more varied. They are also unique to the 'sim' type, so it is possible to leave 'sim' itself out of the settings for --occ= and --B= (e.g., --occ=ID and --occ=sim.ID are identical). Three similarity matrices are built-in to homolmapper itself (blosum62, pam250, and charge). Four more are available as .dict files in the lib/ directory of the standard distribution: asa and chiS score by change in solvent-accessible nonpolar surface area and change in sidechain entropy of folding, respectively, while hphob scores by hydrophobicity (free energy of transfer). The fourth matrix is an identity matrix. The identity matrix is also present as an unformatted .mat file (id.mat), and an additional .mat file provides a more simplistic charge scoring scheme (scharge.mat) for a total of five

independent scoring schemes in the lib/ directory. 30 more schemes are included as .dict files in the standard distribution as a compressed file (lib/more_scores.tar.gz), including a series of BLOSUM (Henikoff & Henikoff, *PNAS* **89:** 10915) and PAM matrices, a series of the related Gonnet matrices (Benner *et al., Protein Eng.* **7:** 1323) used by default in recent versions of CLUSTAL, and additional schemes based on physical properties of the amino acids.. All of these matrices are used by putting the name of the desired matrix in the --occ= or --B= lists; for example, putting --occ=charge on the command line will report charge scoring to occupancy. Matrices that are not built-in must also be imported by specifying the name of the desired .dict or .mat file on the command line.

Importing scoring schemes: To import a scheme, the filename must end with the extension ".dict" (for a formatted scoring dictionary) or ".mat" (for an unformatted matrix). A .dict file must contain a single scoring scheme in a specialized text format which can be converted into Python-readable code at runtime, while a .mat file must also contain a single scoring scheme and simply contains amino acid pairs separated by whitespace, followed by the score for that substitution pair. Importing either type of scheme simply involves putting the filename (with path as needed) on the command line. Alternately, if one wishes to import a number of files from a single directory, the --lib= flag can be used to point to the directory in question (e.g., --lib=/users/fred/homolmapper/lib/.dict will import all .dict files from the homolmapper/lib/ directory in user Fred's home directory). If an environment variable called HOMOLMAPPER_LIB is set to a directory, the --lib flag can be used without an argument to automatically load from that directory. If multiple files

are imported, at most two can actually be used and the files to be used must be specified in the --occ= and/or --B= settings.

If a single .dict or .mat file is imported, it will be used for any unspecified 'sim' scoring (thus, homolmapper a.pdb b.aln foo.mat implies --occ=gap.len as usual, but --B=sim.foo). Examples of both .mat files and .dict files can be found in the lib/ directory of the standard distribution; the process of creating a .dict file is discussed in Chapter 6.

Scoring conservation and variability: It is possible to supply a single similarity scoring type and add 'range' to the scoring flag (either --occ= or --B=). If nothing is specified for the other scoring output, homolmapper will report the normal scores for that type to occupancy and the observed range of values at each position to B factor. As an example, running with --B=charge.range will write conservation of charge to occupancy and variability (measured as the extreme observed values at each position in the alignment) to B factor. This permits the user to distinguish between conserved uncharged positions (occupancy near 0, B near 0) and variable ones with no net conservation of charge (occupancy near 0, but B near 2 because charge scoring spans a range from -1 to +1). This cannot be combined with any other scoring option, because it uses both outputs.

Similarly, one can specify 'std_dev' or 'std_err' *in lieu* of 'range' (--occ=charge.std_dev) to request conservation in occupancy and the standard deviation or standard error of the scores in B factor. Standard deviation is calculated for all sequences (i.e., not sample standard deviation), and standard error is calculated as standard deviation divided by the

square root of the number of scored sequences. As with any procedure inferring significance from homology relationships, a large alignment will have more statistical validity than a small one because of the larger sample size.

Scoring statistics: Starting with version 15.3, homolmapper reports statistics on the calculated occupancy and B-factor scores in the header. Mean, standard deviation, and scoring diversity are reported. Scoring diversity is calculated as the observed Shannon entropy (or uncertainty) of the scores as a percentage of the theoretical maximum for the input alignment length; thus, a scoring scheme which reports a different score for every position in the MSA would have score diversity of 100, and one that reports a single score to every position would have score diversity of 0.

Entropy scoring: As an alternative to the above scoring schemes, one can score by the Shannon entropy or the information content at each position in the alignment by specifying --occ=entropy or --occ=IC (or --B=entropy, etc.). This provides an assessment of which positions in the alignment are informative. Both measures are by default reported in bits (log base 2). This feature is compatible with user-defined amino acid sets (Chapter 3) and expanded amino acid sets (Chapter 5). If fewer than 100 sequences are present in the alignment, a warning will be triggered.

Examples:

1. The settings in range.hmset illustrates the use of charge.range as a scoring scheme. A position with conserved lack of charge (residue 223 is aligned with a total of 11 amino

acids, but has a score of 0 and a range of only 0.1) can readily be distinguished from variable positions (residue 76 has a charge score of -0.07 but a range of 2.00 and is aligned with a total of 18 different residues). Here are alpha carbons for these residues:

```
ATOM    540  CA  ALA A  76       8.723  54.544  48.245 -0.07  2.00           C
ATOM   1631  CA  LEU A 223      33.046  46.974  29.852  0.00  0.10           C
```

Here are pictures of conservation of charge (left; positive charges are blue, and negative charges are red) and variability (right; low range is blue, and high range is red). The greatest conservation occurs near the chromophore (bronze).

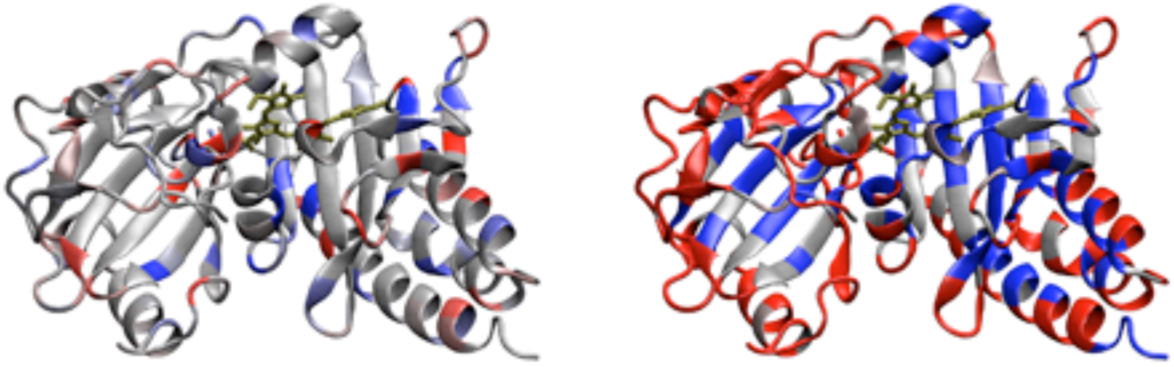

2. SD.hmset uses the same settings as range.hmset, except that the standard deviation of the scores is reported to B factor. Here are alpha carbons for the same two residues:

```
ATOM    540  CA  ALA A  76       8.723  54.544  48.245 -0.07  0.41           C
ATOM   1631  CA  LEU A 223      33.046  46.974  29.852  0.00  0.01           C
```

To further illustrate the use of this option, here are alpha carbons for Arg254 scored by range (top) and std_dev (bottom). Arg254 is quite conserved (>96% identity), but is replaced by an uncharged residue when absent, resulting in a large range. However, the standard deviation is small; Arg254 is one of only four residues with a net charge above 0.5 (occupancy score) and a standard deviation below 0.2 (B factor).

```
ATOM   1845  CA  ARG A 254      25.328  43.715  31.844  0.98  1.00           C
ATOM   1845  CA  ARG A 254      25.328  43.715  31.844  0.98  0.18           C
```

Here are views of this output structure. On the left, the structure is viewed as above and colored by standard deviation. On the right, SD_rad.hmset uses SD.hmset with additional settings to compress the B factor scores into a range between 0 and 2 through use of the autoscale option (discussed in more detail in Chapter 6; SD_rad.hmset includes a recursive call to SD.hmset). In this example, the width of the backbone cartoon is linked to B factor (standard deviation), and the structure is colored by occupancy (conservation). The use of such variable-radius drawing methods can also be useful in presenting such data without the use of color, should that be required.

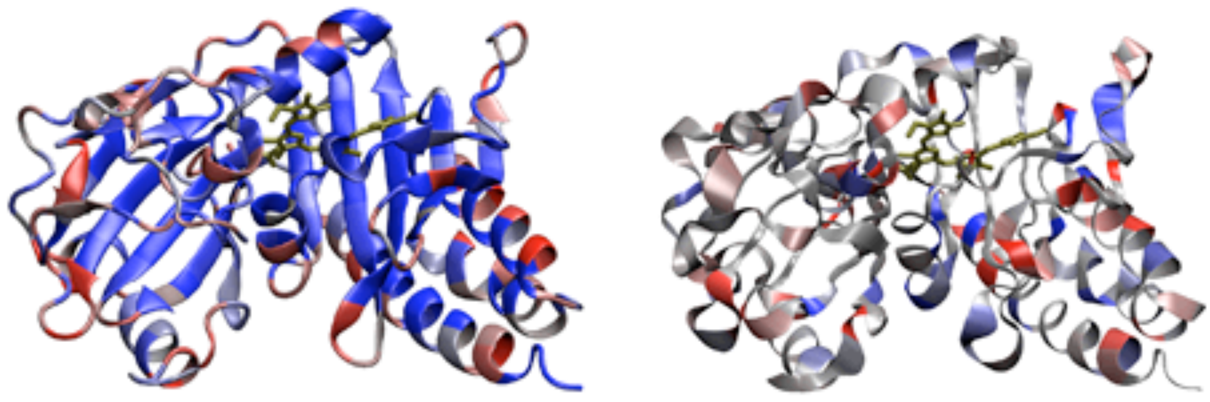

3. asa.hmset uses an imported scheme (the difference in nonpolar surface area) with std_dev scoring. Note that this example uses a .dict file that is present in the working directory; normally this would not be the case, so a complete path would be needed. Residue 76 has a score of 7.43 (meaning that the average residue in the alignment has slightly more nonpolar ASA than the Ala present in the structure) but a range of 64.06, indicating variability. Residue 223 also has considerable variability, with a slightly negative occupancy indicating that the average residue has slightly less nonpolar ASA than Leu. By contrast, residue 208 has a much lower B factor, indicating higher conservation of nonpolar ASA. Here are the alpha carbons for these residues:

```
ATOM    540  CA  ALA A  76       8.723  54.544  48.245  7.43 64.06           C
ATOM   1631  CA  LEU A 223      33.046  46.974  29.852-18.75 44.52           C
ATOM   1506  CA  ILE A 208      33.688  41.185  17.200 -0.38  5.84           C
```

Here is the structure, colored by conservation of asa. Invariant regions are those whose residues stay largely the same size through evolution (grey); this is most true in the chromophore-binding pocket on the right-hand side. Chromophore is shown in bronze.

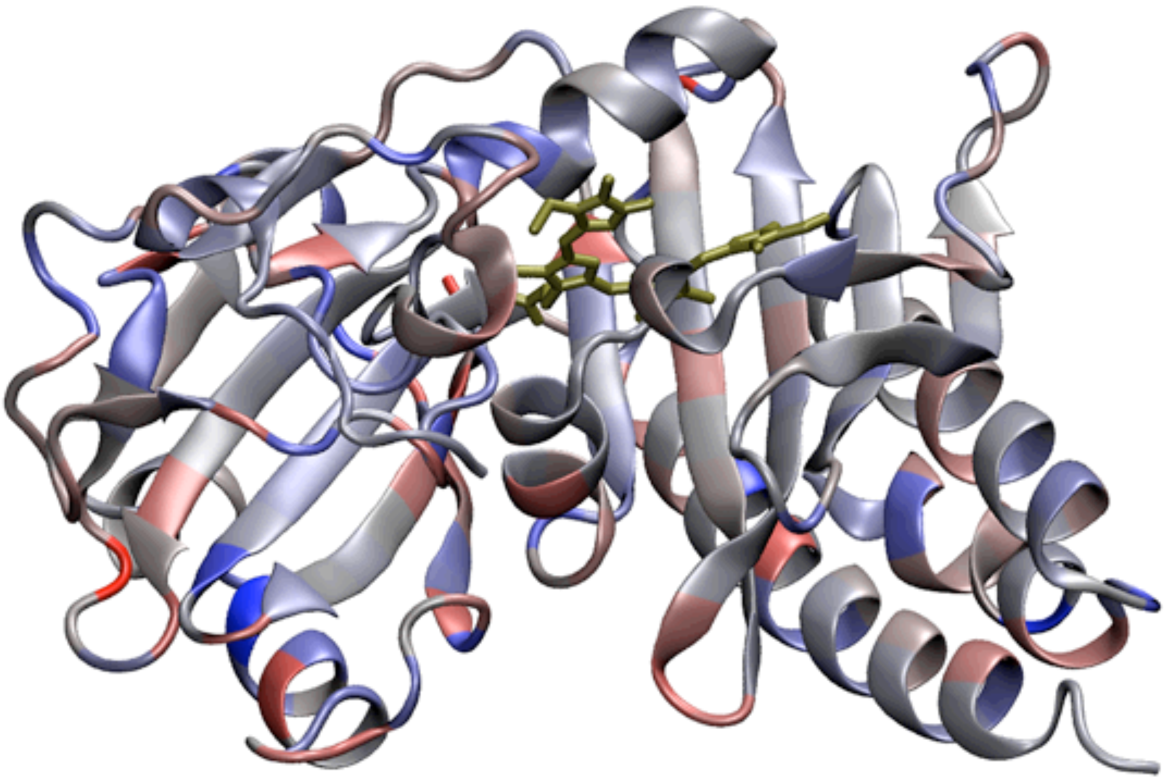

3. The next example compares a homology model made from a distant template to one domain of the authentic structure of a different phytochrome. Gaps and insertions are scored by frequency, because a correct model should place such elements in surface loops. The structures are here shown colored by insertions (absent, dark blue; 3% of sequences, red). The homology model is on the left, and the experimental structure is on the right. In the homology model, the top front helix contains an insertion-rich position in

the alignment. Experimentally, this position is part of a deep trefoil knot which ties together two domains and is not in a region of ordered secondary structure. The top front helix is more conserved and better ordered in the experimental structure.

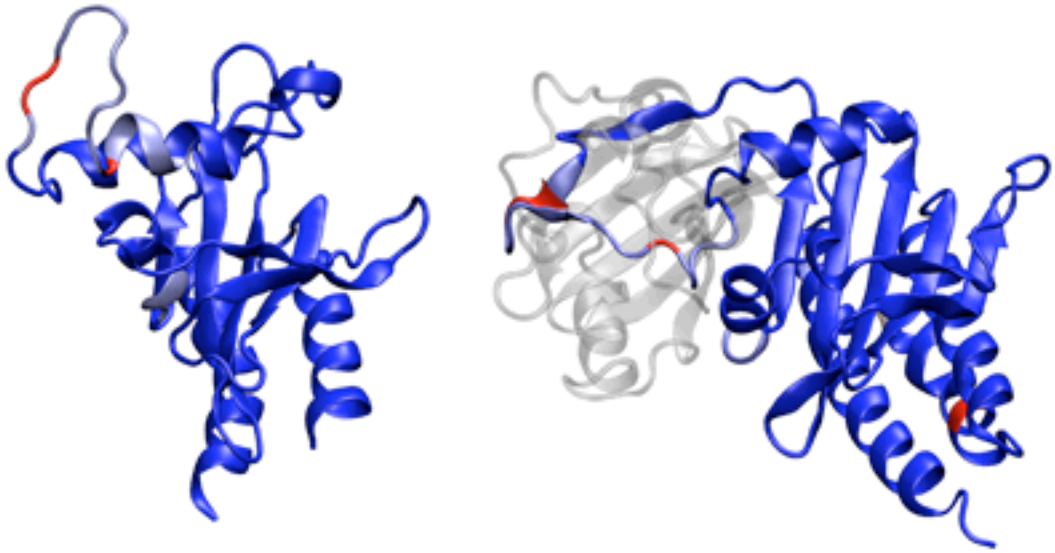

4. In entropy.hmset, entropy scoring (left, colored from blue for low scores up to red for high scores) is compared to identity scoring (right, reversed coloring conventions).

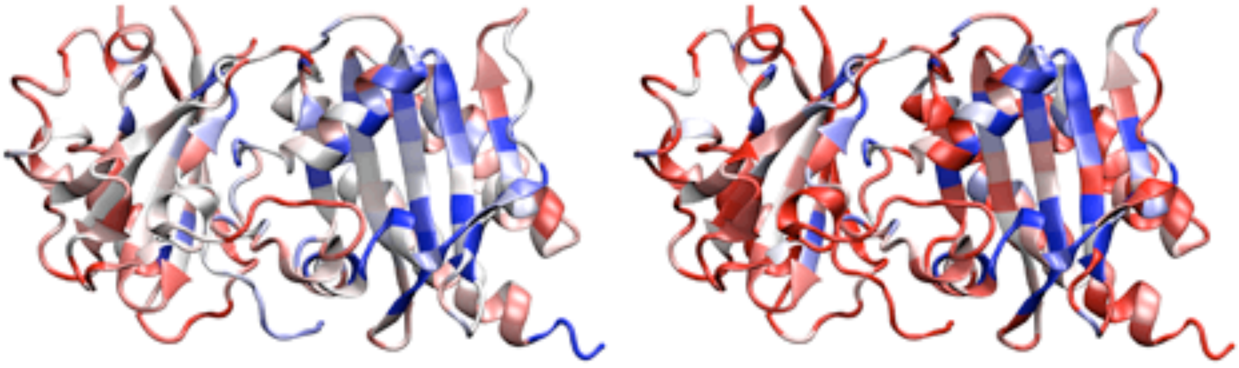

Additional information:

1. Use of “range” by itself implies charge as a scoring scheme. This is also true for std_dev and std_err.

2. Use of “sim” by itself implies ID in the absence of any imports. If a single .dict file is imported, the default will be to score --occ=gap.len --B=sim.XX, where XX is the imported scheme. One can score with the imported scheme and not score occupancy with the default settings (i.e., --occ=gap.len) by either citing it directly (e.g., --B=XX) or, for a single import, by requesting ‘sim’ (e.g., --B=sim). These suppress the default settings for both --occ= and --B=, such that only the requested call is made.

3. Both occupancy and B factor are officially defined as Fortran 6.2 float format (6 columns wide, with 2 digits to the right of the decimal point). Homolmapper actually writes varying precision depending on the size of the number so that a larger range of values can be fit into 6 columns. Rounding is accurate at each level of precision. Strict 6.2 precision can be specified by the --Fortran flag.

4. The charge scoring scheme built into homolmapper is calculated at pH 7 and assigns partial charges to His (+0.1), Cys, and Tyr (both -0.001) to account for partial ionization of these residues in addition to the expected ±1 for Asp, Glu, Arg, and Lys. An alternative scheme that only assigns ±1 to Asp, Glu, Arg, and Lys can be found in the lib/ directory (scharge.mat).

5. Multiple .dict and/or .mat files can be loaded from a directory with the --lib= flag. They should all have different names.

6. The identity matrix is supplied in both .dict and .mat format, and gives identical results to ID scoring. Use of the various identity schemes can thus be used to ensure correct results if the user wishes to tinker with the underlying program.

7. The homology model is provided here as an illustration of another potentially useful application for homolmapper. It should not be considered correct, because it is not.

8. Scoring schemes for occupancy or B-factor can be reported as the Z-score (distance from the mean in standard deviations) by adding "Z_score" to the appropriate flag. The statistics reported in the header are for the scores prior to conversion to Z-scores.

9. The user can choose the base of the logarithm used in entropy calculations and so forth with the --log_base= flag. The default setting of 2 gives units in bits, but some workers prefer a setting of 20 for the 20 canonical amino acids.

10. All matrices loaded at runtime are printed in a list along with the version number if homolmapper is run with the --version flag. This can be used to test the import process.

11. Scoring statistics will not be calculated or reported if the --no_stats flag is used.

2.3 Scoring with reference sequences.

Example directory: ubiquitin
Help flag: --scoring

Background: In homolmapper, gaps and insertions are always scored relative to the matched sequence because they are defined relative to the structure (residues that are not in the structure cannot be reported in the output file). Percent identity and similarity can be scored relative to a consensus sequence or any sequence in the alignment.

Specifying a reference sequence: This is controlled with the --occ= and --B= flags that handle scoring choices in general. The default value ("structure") specifies scoring relative to the structure sequence, i.e. the matched sequence. Alternate values are "use.NAME" (where NAME is the name of a sequence in the MSA file to use as the

reference sequence) and “consensus” (where a consensus sequence will be generated and used as the reference). Reference sequences can differ for occupancy and B factor.

Labeling the reference sequence used: Homolmapper writes the reference sequence method at the end of each line of scoring information in the header.

Example results:

1. The ref_struc.hmset example uses the default method: the reference sequence is the same as the matched sequence. Here are alpha carbons for residues 12-17:

```
ATOM     89  CA  THR    12      29.542  39.020  10.653  1.00 33.33      1UBQ 159
ATOM     96  CA  ILE    13      31.720  36.289   9.176  1.00 66.67      1UBQ 166
ATOM    104  CA  THR    14      30.505  33.884   6.512  1.00 33.33      1UBQ 174
ATOM    111  CA  LEU    15      31.677  30.275   6.639  1.00 33.33      1UBQ 181
ATOM    119  CA  GLU    16      31.220  27.341   4.275  1.00 33.33      1UBQ 189
ATOM    128  CA  VAL    17      30.288  24.245   6.193  1.00 33.33      1UBQ 198
```

The relevant portion of the alignment is shown underlined:

```
1UBQ            -MQIFVKTLTGKTITLEVEPSDTIENVKAKIQDKEGIPPDQQRLIFAGKQLEDGRTLSDY
1BT0            -MLIKVKTLTGKEIEIDIEPTDTIDRIKERVEEKEGIPPVQQRLIYAGKQLADDKTAKDY
1WM3            HINLKVAGQDGSVVQFKIKRHTPLSKLMKAYCERQGLSMRQIRFRFDGQPINETDTPAQL
                 : : *    *. : :.::   .:..:     :::*:.  * *: : *: : :  *  :
```

The structure is matched to 1UBQ in the MSA file (bold). Residue 13 is also Ile in 1BT0, but none of the other residues are present in the other 2 sequences.

2. The ref_use.hmset example chooses a different sequence in the alignment (1WM3) as the reference sequence (although 1UBQ is still the matched sequence). As can be seen above, none of the residues are the same except for the final Ile. Here are the same atoms in the output PDB:

```
ATOM     89  CA  THR    12      29.542  39.020  10.653  1.00 33.33      1UBQ 159
ATOM     96  CA  ILE    13      31.720  36.289   9.176  1.00 33.33      1UBQ 166
ATOM    104  CA  THR    14      30.505  33.884   6.512  1.00 33.33      1UBQ 174
ATOM    111  CA  LEU    15      31.677  30.275   6.639  1.00 33.33      1UBQ 181
ATOM    119  CA  GLU    16      31.220  27.341   4.275  1.00 33.33      1UBQ 189
ATOM    128  CA  VAL    17      30.288  24.245   6.193  1.00 66.67      1UBQ 198
```

3. The ref_cons.hmset example uses a consensus reference sequence. The consensus sequence has both of the matched Ile residues, so each gets a score of 66.67 even though the second Ile is a Val in the matched sequence. Here are the atoms:

```
ATOM     89  CA  THR    12      29.542  39.020  10.653  1.00 33.33      E    159
ATOM     96  CA  ILE    13      31.720  36.289   9.176  1.00 66.67      I    166
ATOM    104  CA  THR    14      30.505  33.884   6.512  1.00 33.33      E    174
ATOM    111  CA  LEU    15      31.677  30.275   6.639  1.00 33.33      F    181
ATOM    119  CA  GLU    16      31.220  27.341   4.275  1.00 33.33      D    189
ATOM    128  CA  VAL    17      30.288  24.245   6.193  1.00 66.67      I    198
```

The SegID field has been overwritten with the consensus sequence (E-I-E-F-D-I, reading from top to bottom). Here are pictures of this region of the structure using the matched sequence (left), 1WM3 (center), and consensus (right) as reference sequences:

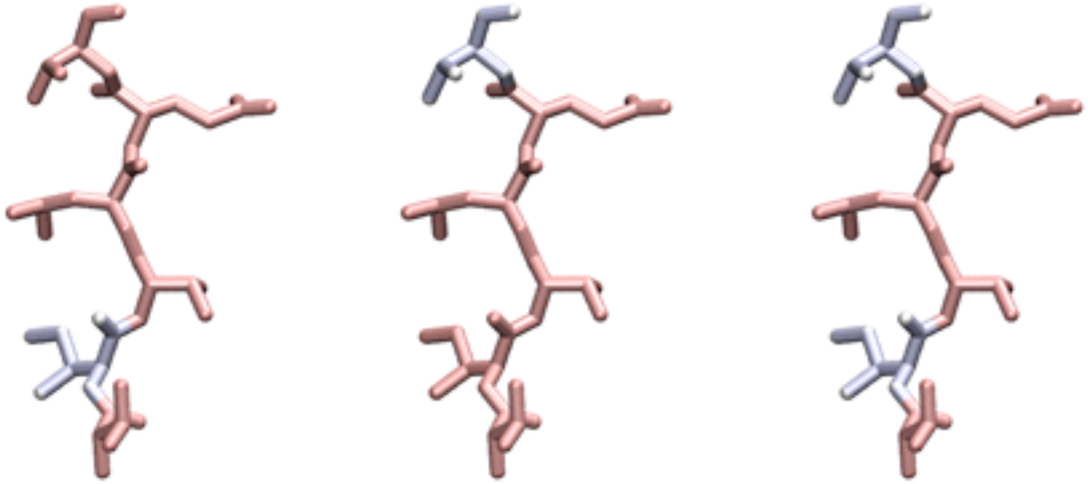

These residues form a beta strand in the structure. They are shown with residue 17 at the top of the figure (orientations are identical).

Additional information:

1. Choosing a reference sequence can be different when working with subfamilies, especially if the matched sequence is not a member of the defined subfamily but is implicitly requested as a reference (Chapter 4).

2. To calculate a consensus, homolmapper simply asks which amino acid is most frequent at each position in the matched sequence. Gaps in the matched sequence are assigned as gaps in the consensus. Instead of single amino acids, it is also possible to generate a consensus with user-specified amino acid sets as a basis.

3. If a consensus reference sequence is used, the consensus sequence will normally be written to SegID for examination.

4. A consensus sequence can be supplied as part of the alignment. The default name for such a sequence is “consensus” (all lower case), but this can be set by the --ext_cons= flag. Such a sequence will be discarded unless a consensus sequence is requested as a reference, in which case it will be used and no consensus sequence will be calculated.

2.4 Matching structure to sequence

Example directory: proteasome
Help flag: --flags

Background: To score information from a sequence alignment onto a protein structure, one must decide which position in the alignment matches which part of the protein

structure. That process is trivial in cases like those in the ubiquitin directory, in which the alignment was made from the structure sequences so that there is an exact match. However, that is frequently not the case. This example uses the crystal structure of the 20S proteasome (1FNT) to illustrate the matching process.

This structure is quite large, with over 9000 residues and more than 40 separate polypeptide chains. The sequence alignment contains individual subunits. By default, homolmapper will run through a series of four algorithms (increasing certainty of finding overlaps but decreasing speed) until it finds something satisfactory. If no significant match is found, an error will trigger. It is possible for the user to control this process by supplying the name of a sequence to match, by skipping some of the algorithms, and by changing parameters used in the more complicated algorithms.

Specifying a sequence: To specify a sequence name, simply put --use=NAME on the command line, where NAME is the desired name. It must match the name in the MSA file exactly (case sensitive). If a name is supplied with the --use= flag, homolmapper will evaluate the sequence corresponding to that name for sufficient overlap to the structure sequence and will not consider the other sequences. The usual algorithms will be applied, and the usual error is triggered if the supplied name is not a significant match. If the name is not found, it is ignored and homolmapper will attempt to find a match by following the default procedure.

Matching algorithms: A maximum of four algorithms are used in searching for matches. The first is a trivial test to see whether the structure sequence is an exact match for one of the sequences in the alignment. The second is a test to see whether one of the polypeptide chains in the structure is an exact match for a sequence in the alignment. The third searches with short blocks of sequence (akin to short peptides or short blocks of sequence) against the chain sequences and in our hands is a successful balance between accuracy and speed. The last is a slower, recursive algorithm which serves as a “backstop” to handle any cases on which the peptide-matching approach fails. The user can skip one or more of these algorithms with the --skip= flag; the default value for this is 0 (meaning 0 are skipped). Setting --skip=1 will skip the first algorithm, while --skip=2 will proceed straight to the peptide-matching algorithm. The recursive algorithm can be specifically requested by setting --skip=3. Both the peptide-matching and recursive algorithms will initially find large regions of overlap and then fill in smaller missing regions when possible, and both have flags to allow control over the matching process. The progress of the various algorithms is reported at high verbosity.

Setting the peptide size: For the peptide-matching algorithm, the parameter accessible to the user from the command line is the size of the peptide blocks used in the matching process. This value must be greater than 2; the default value of 4 seems to perform well. If this parameter needs to be changed, it can be set with the --pep= flag.

Controlling recursive matching: In the recursive algorithm, homolmapper starts with the entire sequence, checks for a match, then cuts a residue off the C-terminus and

checks again, and so forth. If no match is found, it goes back to the entire sequence, cuts a residue off the N-terminus, checks that, then cuts off one-by-one from the C terminus as before, and so on. Then it does the same thing for the next sequence in the alignment. The matched sequence is taken to be the one with the most overlap at this stage. Homolmapper then uses a similar procedure to try to fill in any gaps in the overlap. The user can control the smallest fragment considered significant for the first pass (controlled by the --frag= flag: greater than 2, default 4) and the smallest fragment filled in at the second pass (via the --fill= flag: greater than 0, default 2).

Defining a significant match: Homolmapper requires that the overlap between structure and sequence be above a threshold to be considered significant. By default, there must be 20 residues that match between the .pdb and MSA sequences, or an error is triggered. This parameter can be changed with the --match= flag, but must always be > 0.

Defining a significant chain: In parsing the input PDB file, homolmapper will only attempt to match residues that belong to a polypeptide chain of at least a minimum length (by default, 10 residues). This cutoff can be reset via the --MinChain= flag.

Examples:

1. match_def.hmset allows homolmapper to look for matches itself using default settings. The matched sequence is pre5 (233 residues overlapping chain F of the PDB).

2. use_pre.hmset explicitly uses pre5 as a test sequence to match. The same overlap is found, and the output pdb files are identical except for the timestamp in the header.

3. use_scl.hmset instead uses scl1 as a test match; 238 residues in chain A are matched. This is not found by default, because pre5 appears first in the MSA and is also an exact match. HomolMapper stops searching with any exact match.

4. use_psm.hmset uses the third sequence in the alignment as a candidate. HomolMapper uses the peptide-matching algorithm. 36 residues of overlap are found, spread over chain E. Here are views of the three "use" examples (2-4, left to right) with unmatched portions of the structures shown as transparent grey ribbon:

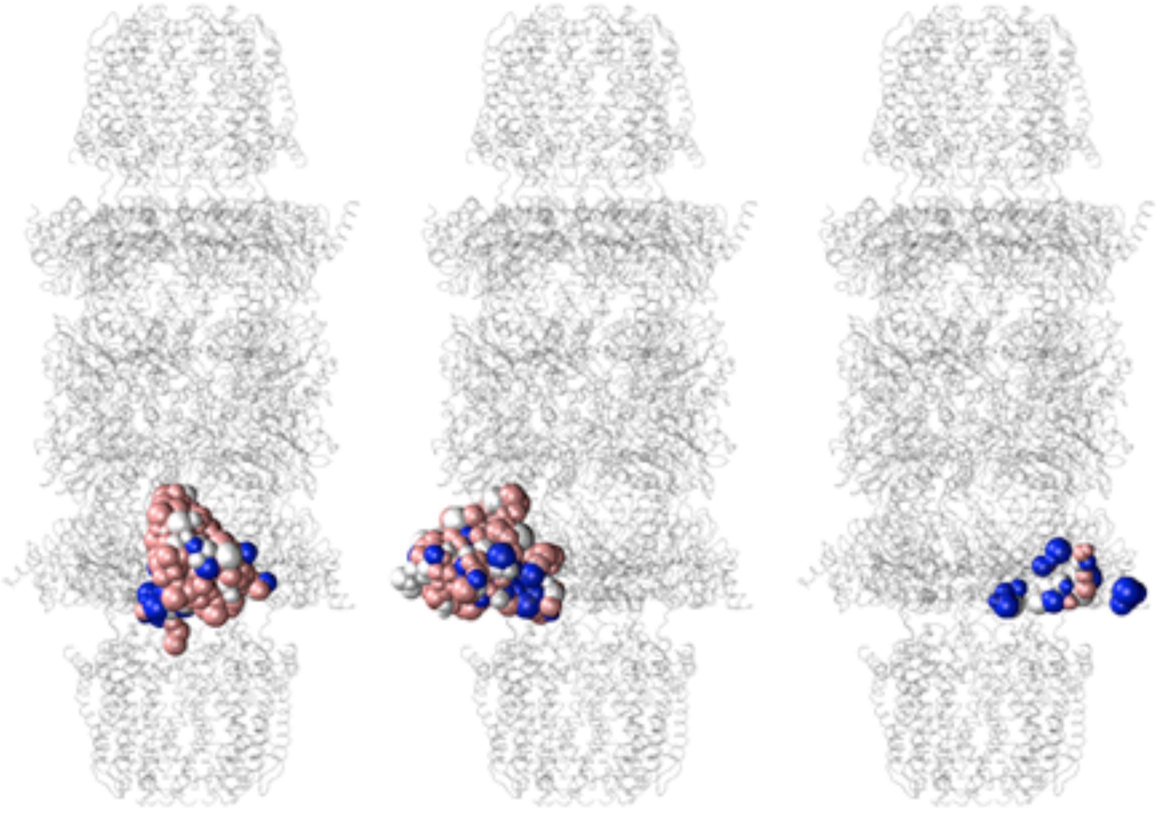

Additional information:

1. This example has deliberately been chosen to emphasize the role the user can play in matching sequences. However, the default settings will handle most cases well.

2. The runs in this example are noticeably slower due to the anomalously large structure file. Over half the time is spent building new atoms for the output PDB file.

2.5 Alignment formats

Example directory: HO
Help flag: --alignments

Background: The CLUSTAL format for protein alignments is only one of many formats that are used to store and present such data. The CLUSTAL program can convert a number of other formats into CLUSTAL-format alignments, as can a number of web-based tools. However, it is also possible to use several other common formats directly within homolmapper: PIR/NRBF, FASTA, MSF, GCG, NEXUS, and PHYLIP. Homolmapper uses the extension of the file to decide which format an alignment is in. The understood extensions are .aln (CLUSTAL format), .pir (PIR/NBRF format), .fasta (FASTA format), .msf (MSF format), .gcg (GCG format) .nex or .nxs (NEXUS formats), and .phy (PHYLIP formats).

PIR format: The PIR format currently parsed by homolmapper is PIR/NBRF format; this is not to be confused with PIR/CODATA format, even though both are unfortunately known as PIR format in different instances. Here are a few lines of a PIR/NBRF file, which is the *supported* PIR format:

```
>P1;neimehemo

-----------------------------------------------MRASADFARPASL
SLTDKGKL------------MSETENQAL------TFAKRLKADTTAVHDSVDNL-----
-----------------------------------------VMSVQPFVSKENYIKFLKL
QSVFHKAVDHIYKDA---------------------------------------------
------------------ELNKAIPELE-YMARYDAVTQDLKDLGE--------------
-----------EPYKFDKELPYEAGNKAIGWLYCAEGSNLGAAFLFKHAQKLDY------
-------------------------------------NGEHGARHLAPHPDGR-------
-------------------------------------------GKHWR-AFVEHLNALNL
T-----PEAEAEAIQGA--REAFAFYKVVLRETFGLAADAEAPEGMMPHRH---------
------------------------------------------------------------
------------------------------------------------------------
------------------------
*
>P1;psepupga2
```

The use of '*' at the end of the sequence and >P1; before the sequence name are hallmarks of this format.

FASTA format: FASTA format is somewhat similar to the PIR/NBRF format. It is the input format (and default output format) for the increasingly popular MUSCLE program (Edgar, R. C., *NAR* **32:** 1792). Here are the equivalent lines in FASTA format:

```
>neimehemo neimehemo        Len:   684  Check: 2897  Weight:   1.00 684 bp
-----------------------------------------------MRASADFARPASL
SLTDKGKL------------MSETENQAL------TFAKRLKADTTAVHDSVDNL-----
-----------------------------------------VMSVQPFVSKENYIKFLKL
QSVFHKAVDHIYKDA---------------------------------------------
------------------ELNKAIPELE-YMARYDAVTQDLKDLGE--------------
-----------EPYKFDKELPYEAGNKAIGWLYCAEGSNLGAAFLFKHAQKLDY------
-------------------------------------NGEHGARHLAPHPDGR-------
-------------------------------------------GKHWR-AFVEHLNALNL
T-----PEAEAEAIQGA--REAFAFYKVVLRETFGLAADAEAPEGMMPHRH---------
------------------------------------------------------------
------------------------------------------------------------
------------------------
>psepupga2 psepupga2        Len:   684  Check: 5628  Weight:   1.00 684 bp
```

As with the PIR format, the FASTA format is *sequential* (each sequence is shown by itself, followed by the next sequence) rather than *interleaved* (sequences are shown together, as in CLUSTAL .aln files).

GCG/MSF formats: Several formats have been called "GCG" or "MSF." HomoImapper considers the MSF format to be an interleaved format with a large header, while the GCG format is a sequential format with the header information distributed over the various sequences. Both are supported with the appropriate choice of extension.

NEXUS format: The NEXUS format is an interleaved format with a header. It is the default format for the popular PAUP phylogeny program. NEXUS files are associated with two extensions: .nex and .nxs. HomoImapper supports both extensions.

PHYLIP formats: There are both sequential and interleaved variants of the PHYLIP formats used by the popular PHYLIP phylogeny programs. HomoImapper can handle both of them.

Other formats: Alignments can be converted into a supported format in several ways. Many alignment packages have the ability to generate an alignment in several formats. Some, such as CLUSTAL, can also convert an alignment between formats without changing the alignment. There are also several web-based servers which use the ReadSeq engine or others to convert alignments.

Examples:

1. The standard.hmset file uses the standard.aln file with the default scoring choices to score the structure. Here are different views of the structure colored by length of gaps (occupancy, left) and percent identity (B factor, right):

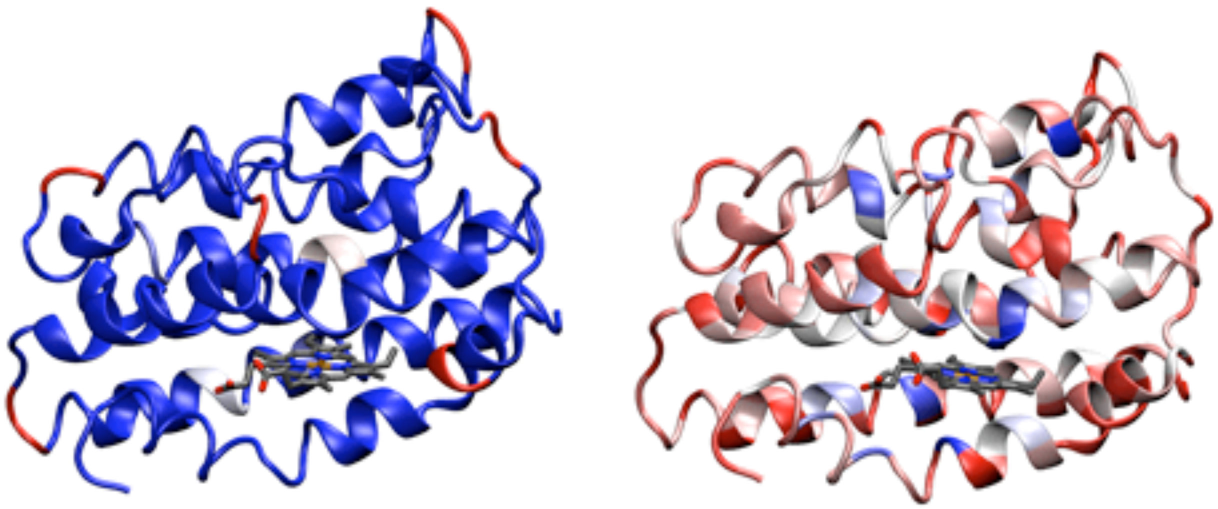

2. The .hmset files with filenames that are MSA extensions (fasta.hmset, nxs.hmset, etc.) use different alignment files containing the same MSA in different formats. The various files will all differ in only three lines: timestamp and the two header lines that contain the name of the MSA file.

Additional information:

1. There is some variability in what is considered canonical in some of these formats. Homolmapper uses certain (hopefully inviolate) features in parsing, permitting more flexibility in handling input files.

2. The --alignments flag provides additional information about how homolmapper parses the various formats. This is useful for troubleshooting alignment-import problems.

3. The character used to indicate a gap varies among formats, with '-' and '.' being most common. Homolmapper currently supports only these two characters.

4. Homolmapper itself can write out the parsed alignment in CLUSTAL format by using the --punch=aln flag. The character used for gaps in the input file will be preserved. If this is not the default '-' character, the header line in the punched .aln file will indicate what character is being used for gaps.

5. MUSCLE-generated .aln files can have either a header indicating the origin of the file as MUSCLE or a header that is strictly CLUSTAL compliant. Both are supported.

Chapter 3: Beyond B factor

Overview: Homolmapper writes most homology information to two fields: occupancy and B factor. Most structure viewers are able to display the information in these two fields, for example by color coding. Most of the other fields available for each atom are associated with identifying the atom (residue name and number, for instance) or with locating it in space. However, three other fields are available for other information in the 1996 PDB standard, and homolmapper currently can use two of them: SegID (segment ID) and element.

3.1 Basic uses of the SegID field

Example directory: ubiquitin
Help flag: --extras

Background: The SegID field is newer than fields such as occupancy or B factor. It is officially deprecated but is still used in some viewers and by other applications. It contains fewer columns than occupancy or B-factor (4 instead of 6); the reduced size means that a narrower range of numbers can fit, and it also means that numbers are reported with less precision. Moreover, SegID is not intended for continuous numerical input. For these reasons, homolmapper does not use SegID for scoring information in the same way that occupancy and B factor are used.. Instead, the SegID field can be used to write match numbering (the amino acid number in the matched sequence in the MSA that corresponds to each amino acid in the structure) or to write a consensus sequence. The

SegID field can also be used to report information about user-defined amino acid sets or mutations.

Reporting matched amino acids: HomolMapper matches the structure sequence to a single sequence in the MSA to decide which residues in the structure correspond to which parts of the alignment. Each scored amino acid in the structure is therefore uniquely matched to a position in the alignment. Internally, this is tracked by the amino acid number in the matched sequence, starting from 1 at the N-terminus. To see this correspondence, simply add --Seg=numbering to the command line. The amino acid number for each residue in the MSA will be written to the SegID field of each atom of the corresponding residue in the output .pdb file. Unscored residues are assigned a value of "UNSC" (for unscored). This value can be assigned to unscored atoms in the context of other SegID outputs by using the --clobber_Seg flag.

Reporting a consensus sequence: HomolMapper can write a consensus sequence to the SegID field with the --Seg= flag by using --Seg=consensus. By default, this is calculated by testing which amino acid is the most common at each position in the MSA; if multiple amino acids are equally frequent, a single winner is chosen alphabetically. However, this process varies with user-defined amino acid sets. The consensus sequence is reported to SegID as the consensus amino acid corresponding to each amino acid in the structure. Using a consensus reference sequence (Chapter 2) will trigger --Seg=consensus automatically if no mutations, highlights, or motifs are supplied. Such information is written to SegID as well and takes precedence.

Examples:

1. The seg_num.hmset file gives an example of --Seg=numbering with the 1WM3.pdb structure. This structure starts at residue 17 in the structure, but the same residue is amino acid 1 in the alignment. The first 2 alpha carbons look like this:

```
ATOM      2  CA  HIS A  17     -11.570   8.953   6.350  0.00 100.0      1    C
ATOM     12  CA  ILE A  18      -7.910   9.377   5.605  0.00 33.33      2    C
```

2. seg_cons.hmset uses --Seg=consensus with the same settings on the same file. Here are the first two alpha carbon atom:

```
ATOM      2  CA  HIS A  17     -11.570   8.953   6.350  0.00 100.0      H    C
ATOM     12  CA  ILE A  18      -7.910   9.377   5.605  0.00 33.33      M    C
```

Additional information:

1. More information about using the SegID field with homolmapper is available by using the --extras flag.

2. The SegID field was originally conceived as an accessory to chain ID. For instance, in an antibody structure, one can use the SegID field to equate the heavy chains. Because the heavy chains are separate chains, they have distinct chain ID values, but can have the same SegID. This means that viewers that recognize SegID typically are not prepared for this field to contain a continuous range of floating-point numbers, as is the case for occupancy and B factor. The more recent deprecation of the SegID field in the official PDB format makes it unlikely that the viewers will change.

3.2 Scoring with user-defined residue sets

Example directory: PcyA
Help flag: --scoring (also sample.slop)

Background: Scoring by identity considers all amino acids to be equally different, while similarity matrices assign values to different pairs of amino acids based on different methods. There are instances that are not well-suited to either approach. For example, if one wanted to examine potential hydrogen bonding donors at neutral pH, one would want to define a set of amino acids that are the likely donors and consider them all as equivalent. One could build a substitution matrix reflecting this scheme and then import it as a .dict file, but this would be fairly tedious. As an alternative, homolmapper permits user-defined amino acid sets. These sets may be formally considered degenerate, because unequal amino acids are considered to be equal.

Scoring with a degenerate set: If only a single degenerate set is desired, the --occ= and/or --B= flags should contain 'degen.' followed by a list of amino acids in 1-letter code. Thus, --B=degen.DE would equate Asp with Glu. Homolmapper will then report the percent at each position that are Asp or Glu in B factor. This scoring method is not defined relative to any reference sequence.

Syntax for degen definitions: Several shorthands can be used after degen. For example, --B=degen.DE can also be written as --B=degen.-, while --occ=degen.KR may be written as --occ=degen.+ ('-' is not a gap in degen definitions). Use of '^' or '!'

characters will invert the definition, so that --occ=degen.^GP will report the percentage of residues at each position that are not Gly or Pro. The '@' or '*' characters can be used as wildcards meaning any residue. The actual set of amino acids generated by each definition is added to the header. A definition containing a single amino acid will give rise to a set containing one amino acid.

Working with multiple user-defined residue sets (sloppy scoring): The user can also define multiple amino acid sets encompassing some or all of the amino acids. Such sets are scored relative to a reference sequence; at each position, the percent of sequences having an amino acid belonging to the same set as that of the reference at each position is reported. This scheme is called sloppy scoring, and is requested by placing 'sloppy' in the --occ= or --B= request lists. Consensus sequences are determined using these set definitions if they are provided. A special option, --Seg=assignment, will cause the set definition used for each position in the matched sequence (*not* the reference sequence) to be written to SegID.

The user-defined amino acid set definitions are loaded into homolmapper at runtime from an accessory file called a .slop file. A .slop file is just a text file containing one set definition per line. The '#' character indicates comments, and 1-letter or 3-letter code can be used. Amino acids that are not defined in the .slop file are each placed into their own group, and each amino acid can only be in 1 group. The shortcuts used in degen definitions can also be used in .slop files, but inverting definitions is likely to place the same amino acid in more than 1 group. Two lines from a .slop file are shown here:

```
+               # equivalent to K       R (degen.+ is the same as degen.KR).
N       Q
```

User-defined residue sets and entropy scoring: A common criticism of scoring schemes such as identity, entropy, or information content is that these schemes do not reflect the fact that some residues are more chemically similar than others. One approach that has been combined with entropy scoring to correct for such problems is to divide the amino acids into small, chemically similar sets (for an example, see Mirny & Shakhnovich, *JMB* **291:** 177). Entropy and information scoring can use .slop files to permit such an approach.

Examples:

1. ex_degen.hmset uses two degen definitions to examine the structure of cyanobacterial PcyA with an alignment of 70 ferredoxin-dependent bilin reductases (FDBRs). Asp and Asn are considered equivalent in occupancy, and Asp and Glu are considered equivalent in B factor. A consensus sequence is written to SegID as well with --Seg=numbering; this consensus considers all amino acids to belong to one-residue sets, because no .slop file is provided. Three alpha carbons are shown here:

```
ATOM   1663  CA  CYS A 104       8.283 -23.326  41.890  0.00  0.00      A    C
ATOM   1679  CA  ASP A 105       5.198 -21.726  43.417 85.71 87.14      D    C
ATOM   1697  CA  ILE A 106       4.138 -22.123  47.060  0.00  0.00      L    C
```

No Asp, Glu, or Asn are found at positions 104 and 106, while residue 105 is frequently one of these residues. The consensus sequence is ADL.

2. ex_cons.hmset uses the definition in Hbond.slop to score relative to the structure (occupancy), to score relative to the consensus sequence (B factor), and to write a consensus sequence (SegID). The same three atoms are shown. Cys104 is present in the structure, so occupancy is scored relative to the set Cys belongs to (which is rare). B factor is scored relative to the consensus (AFGI, etc.) and therefore gets a higher score. Asp105 and Ile106 in the structure both belong to the most common set, and hence the occupancy and B factor scores are identical. The consensus sequence is different due to the different amino acid set definitions.

```
ATOM   1663  CA  CYS A 104       8.283 -23.326  41.890 14.29 85.71      AFGI C
ATOM   1679  CA  ASP A 105       5.198 -21.726  43.417 87.14 87.14      ED   C
ATOM   1697  CA  ILE A 106       4.138 -22.123  47.060 100.0 100.0      AFGI C
```

4. assign.hmset uses the definition in Hbond.slop without any --occ= or --B= information. In this case, sloppy scoring is inferred from the .slop file and written to B field, while occupancy is the unspecified default (equivalent to --occ=gap.len). In addition, this example sets --Seg=assignment, so the SegID field lists which sloppy definition is associated with each residue in the matched sequence. Alpha carbons for residues 104 to 106 are shown here:

```
ATOM   1663  CA  CYS A 104       8.283 -23.326  41.890  0.00 14.29      KRST C
ATOM   1679  CA  ASP A 105       5.198 -21.726  43.417  0.00 87.14      ED   C
ATOM   1697  CA  ILE A 106       4.138 -22.123  47.060  0.00 100.0      AFGI C
```

Here, residues 105 and 106 are assigned to the same group as the consensus (example 3 above). However, residue 104 is an atypical Cys in the structure sequence and hence is assigned to a different set than is the consensus residue. Note that only the first 4

amino acids in each set are shown, because of the size of the SegID field. Nevertheless, all residues are used in scoring.

5. shorthand.hmset gives an example of the degen shorthands. Occupancy is scored with --occ=degen.^+- (not Asp, Glu, Arg, or Lys), and B factor is scored as --B=sloppy without a .slop file, so each amino acid is assigned to its own group.

```
ATOM   1663  CA  CYS A 104       8.283 -23.326  41.890 100.0 10.00           C
ATOM   1679  CA  ASP A 105       5.198 -21.726  43.417 12.86 80.00           C
ATOM   1697  CA  ILE A 106       4.138 -22.123  47.060 100.0 18.57           C
```

In this case, occupancy of residue 105 is the inverse of B factor for the other schemes. Residues 104 and 106 are 100% for occupancy because there are no Asp or Glu at these positions, in keeping with the zero B factor scores in ex_degen.pdb. Here are views of these residues from ex_degen.pdb (left) and shorthand.pdb (right), with the residues shown as stick figures colored by atom type and homology shown as a color-coded transparent surface:

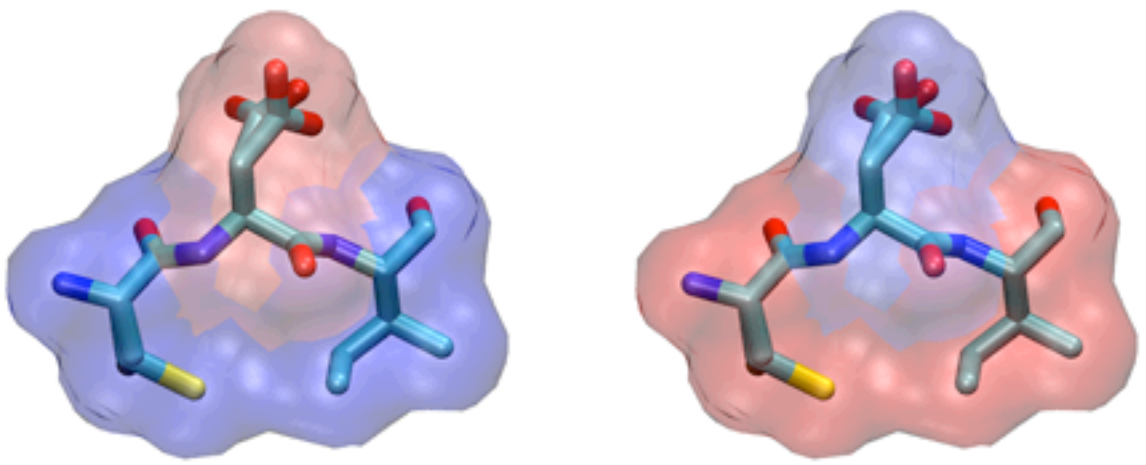

Additional information:

1. The inversion characters and wildcards in degen scoring can trigger shell errors if used incautiously (for example, using '!' confuses tcsh). Any flag can be placed within single or double quotes to help avoid shell problems.

2. The actual amino acids used in a degen scoring run are written to the header line.

3. If sloppy scoring is requested and no .slop file is provided, each amino acid will be in its own set. This gives the same result as identity scoring.

4. More information about .slop files can be found in the sample file contained in the standard distribution (lib/sample.slop).

5. The set definitions generated from a .slop file are printed to stdout at high verbosity.

6. The --supersloppy flag permits amino acids to be members of more than one set. Assignment is based on the order of the sets in the .slop file. Results must be interpreted with caution.

3.3 Mutations

Example directory: phytochrome
Help flag: --extras (also example.mut)

Background: In addition to the applications discussed above, the SegID field is also used by homolmapper for scoring mutations, highlights, and multi-residue motifs. Such information takes precedence over other uses of SegID. Homolmapper can be used to

display the locations of mutations in homologs on a known structure and can also be used to score the mutations relative to the MSA.

Defining mutations in homolmapper: Mutation definitions can be supplied from the command line by the --mut= flag, by supplying a .mut file on the command line, or both. The .mut file is a text file with '#' used to indicate comments; one mutation is defined per line. Each line follows this format:

```
asphya3 323     H       L       LOF
```

The first word is the sequence name, the second is the residue number, and then 'from' (wildtype) and 'to' (mutant) amino acid codes follow. The last word is an optional category, such as a phenotype or some other text reference. For non-substitution mutations, the codes 'DEL', 'INS', 'NTR', 'CTR', 'FRM', and 'UNK' can be used for deletions, insertions, N-terminal truncations, C-terminal truncations, frameshifts, and unknown mutations, respectively.

The --mut= flag uses a similar syntax, but the '.' character is used as a separator so that the definition is in a single flag. The above mutation would thus be described on the command line as --mut=asphya3.323.H.L.LOF. Multiple --mut= flags can be on a single command line, but only one .mut file is currently handled. Homolmapper will test each definition for a correct name, correct wildtype residue, and defined mutant residue (or code). Problematic definitions and mutations that fall within gaps in the structure sequence will be ignored.

When to use what: If a single mutation is being provided, the command-line option will probably be faster. If many mutations are scored, the .mut file will be preferable. The .mut file can also be saved for reuse or for updating as more data become available.

Scoring mutations: By default, homolmapper writes the category information to the SegID field if mutations are supplied. For unsupplied categories, a SegID of 'UNK ' (unknown) will be assigned, while a SegID of 'mMUT' (multiple mutants) indicates that multiple mutations with different categories are mapped to the same position on the structure. The SegID field is 4 columns wide and left-justified. Therefore, categories that are too long will be truncated, and categories that are too short will have spaces added to the right-hand side. If 'UNK ' is a sufficient category and only categories are requested, the 'to' amino acid and category are optional. If 'to' (mutant) amino acids are provided, homolmapper can also score the mutations in a variety of other ways, as follows:

--Seg=natural	report % of sequences that have the mutant amino acid naturally present.
--Seg=conservation	report % of sequences that have the wildtype amino acid.
--Seg=gap	report % of sequences that have no amino acid at the position of the mutation.
--Seg=insert	report % of sequences that have any amino acid at the position of the mutation.
--Seg=Ntrunc	report % of sequences with nothing at or N-terminal to the position of the mutation.
--Seg=Ctrunc	report % of sequences with nothing at or C-terminal to the position of the mutation.
--Seg=tolerated	assess mutations by type, using above scoring options.

None of these schemes require the use of a reference sequence.

Using --Seg=tolerated: This option provides a fast, crude assessment of the extent to which the mutations might be tolerated. Substitutions are scored using --Seg=natural to measure how frequently the mutant residue occurs naturally in the aligned homologs. Deletions are similarly scored by --Seg=gap, and insertions by --Seg=insert. N-terminal truncations are scored by --Seg=Ntrunc, and C-terminal truncations and frameshifts are scored by --Seg=Ctrunc. Unknown mutations are assessed by --Seg=conservation to determine whether the mutated position is conserved.

Examples:

1. mut_cat.hmset will run the phytochrome example with a number of reported mutations (contained in init.mut), including a sequence mismatch and several within gaps in the structure. These will trigger warnings, but the run will proceed normally.

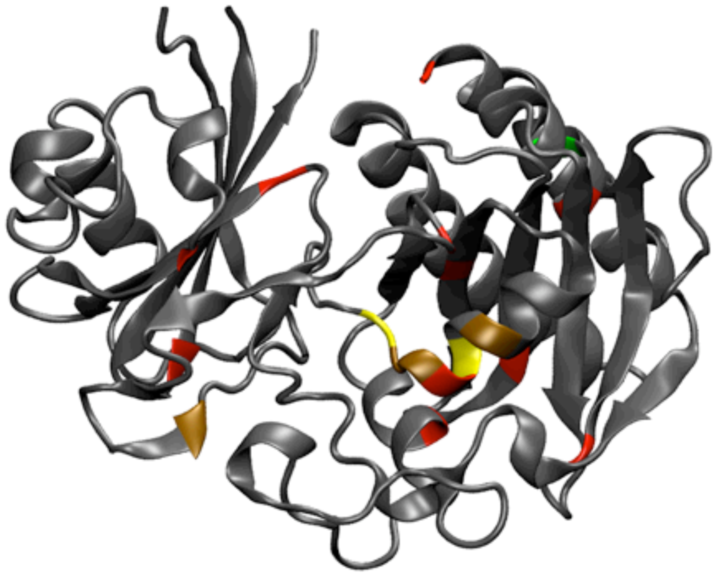

The resulting figure demonstrates that a number of reported loss-of-function mutations (red) cluster on the right-hand side of the structure.

Here is the alpha carbon of one mutant residue. The SegID field now contains the category label “LOF “ (loss-of-function). Note that occupancy and B factor can be scored as desired; mutation handling is independent.

```
ATOM   1540  CA  ARG A 213      28.851  45.269  16.256  0.00 98.36      LOF  C
```

2. mut_tol.hmset will use the same files to score the mutations (--Seg=tolerated) rather than report categories. 129 lines differ between the 2 resulting PDB files. The same atom is shown for comparison. The SegID score of 0.00 indicates that the loss-of-function mutant residue does not occur in any of the aligned homologs and is therefore unlikely to be tolerated.

```
ATOM   1540  CA  ARG A 213      28.851  45.269  16.256  0.00 98.36      0.00 C
```

Note that the scoring in occupancy and B field proceed normally and no not make any use of the mutations, highlights, or motifs. Only SegID is used for this information.

Additional information:

1. More information about SegID can be found at the PDB.

2. Additional background on .mut files can be found in the example file provided in the standard distribution (utils/examples/example.mut).

3.4 Highlights

Example directory: phytochrome
Help flag: --extras (also example.mut)

Background: Given a structure and an alignment, a recurring question is where all of a given amino acid in one of the homologs will fall on the structure; for instance, in planning a cross-linking experiment, a reasonable approach would be to predict the accessibility of Cys or Lys residues by examining their locations in the protein of interest projected onto a known structure if the protein of interest has not itself been examined structurally. Homolmapper can map such residues quickly using highlights.

Using highlights: Highlight definitions can be provided via a .mut file, the --mut= flag, or both. The highlight syntax is a modified mutation syntax with 'ALL' in place of the residue number and with no mutant residue:

```
cph1  ALL   TYR   C1Y
```

Highlights only use category scoring. If no category is supplied, 'HLIT' will be used. If multiple highlights with different categories fall on the same position, a category of 'mHLT' (multiple highlights) will be assigned. Highlights can be mixed with mutations in a single run. If highlights are mixed with mutants, highlights will be given precedence.

Example:

1. highlight.hmset illustrates use of the --mut= flag rather than .mut files and demonstrates highlighting by mapping the Cys residues of a homolog, Cph1, onto the

structure of the crystallized protein. The residues in question are given a SegID of “C1C “ (with one space to the right because this is only 3 characters long). Cys24 in the structure is not present in Cph1, while Cys289 is a Cys in both proteins. Leu305 in the structure is a Cys in Cph1 (named cph1 in the MSA). The highlighted structure can be colored by SegID to give results like this:

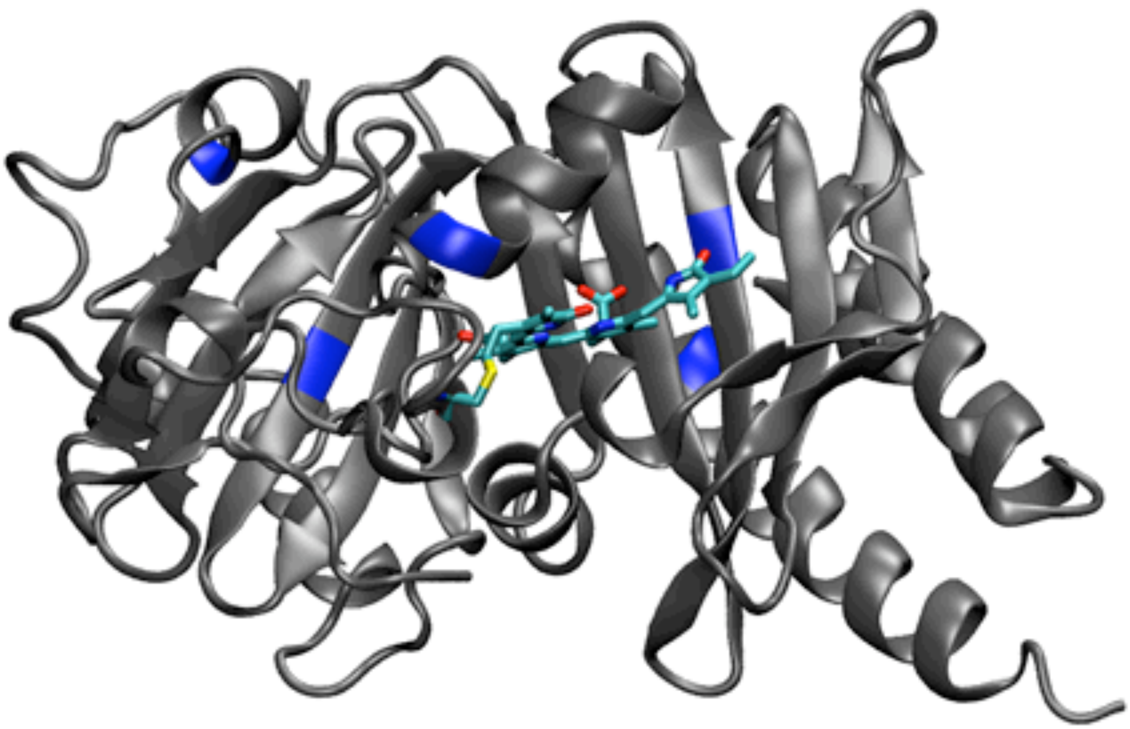

Here are alpha carbons for all 3 residues:

```
ATOM    158  CA  CYS A  24      29.193  32.903  17.364  0.00 37.61           C
ATOM   2102  CA  CYS A 289      41.141  49.505  26.447  0.00 86.89      C1C  C
ATOM   2233  CA  LEU A 305      34.993  58.723  27.682  0.00  4.10      C1C  C
```

Additional information:

1. As shown in the example, entry of a single highlight via the --mut= flag is much more convenient than creating a .mut file.

2. As with mutations, highlights do not preclude any scoring options for --occ= or --B= scoring requests.

3.5 Multi-residue motifs

Example directory: phytochrome
Help flag: --extras (also example.mut)

Background: It is also possible for the user to use --mut= or a .mut file to define a sequence motif consisting of more than one residue. The residues need not be adjacent in primary sequence (or in the tertiary structure). One or more motif can be handled in a single run. Motifs are specified with --Seg=motif.

Using --Seg=motif: For this option, every line of the .mut file (and every supplied --mut= definition) is assumed to be part of a motif. Each line must therefore be a valid definition, although they can fall within gaps in the structure sequence. Uniquely, the wildtype amino acid value can be a degenerate definition rather than a single amino acid. This permits motifs to be defined with multiple allowed residues at a given position. For example, specifying ST as allowed residues would permit the motif to contain either Ser or Thr, for instance in defining the recognition motif for a Ser/Thr protein kinase. Homolmapper will report the percentage of sequences that have all positions matched (including those in gaps in the structure) to SegID.

Motifs are defined via a modification of the mutation syntax. Categories are ignored, and the ‘to’ amino acid field is a motif identifier (motif ID). If a single motif is being defined, the

motif ID is optional. However, if multiple motifs are defined in a single run, it is necessary to provide motif ID values for every residue in every motif. The space character is not a valid motif ID. No amino acid can currently be in more than one motif.

If multiple motifs are present, the score for each motif is written to SegID as usual. Additionally, the element field is overwritten with the motif ID at each position, so that the user can keep track of which motif is which. The element field is two characters wide. Motif ID is thus reported as the first two characters of the supplied value. If the first two characters are the same for different motifs, no distinction will be made between those two motifs. If a motif ID is more than two characters long, it will be truncated to fit the element field. HomolMapper replaces the element field of residues that are not in any motif with a blank field.

Examples:

1. one_mot.hmset uses a motif definition at residues 198, 203, 263, and 267 of the structure. The motif is present in 91.8% of the sequences in the MSA. Here are the alpha carbons of residue 213 (unscored) and residue 203 (in the motif, with SegID 91.8):

```
ATOM   1540  CA  ARG A 213      28.851  45.269  16.256  0.00 98.36           C
ATOM   1469  CA  PHE A 203      41.954  40.522  13.003  0.00 28.69      91.8 C
```

2. mult_mot.hmset uses the same motif as the previous example with a motif ID of ‘ld’ (lower case). A second motif is defined, with a motif ID of ‘C10’ (which will be truncated to C1 in the output PDB file). Here are alpha carbons for residues 213 and 203, as above, along with residue 207, which is in the second motif:

```
ATOM   1540  CA  ARG A 213      28.851  45.269  16.256  0.00 98.36
ATOM   1469  CA  PHE A 203      41.954  40.522  13.003  0.00 28.69      91.8ld
ATOM   1498  CA  ASP A 207      36.370  38.526  16.656  0.00 100.0      34.4C1
```

Note that the element field is now overwritten with the motifID, and ‘C10’ has been truncated to ‘C1’ for residue 207. The SegID and element fields are immediately adjacent, so using ‘10’ as a motifID would have given the appearance of a larger SegID with a single number (34.410, where the 10 is actually the element). Here is the structure, colored by SegID to show the motifs with chromophore in light blue:

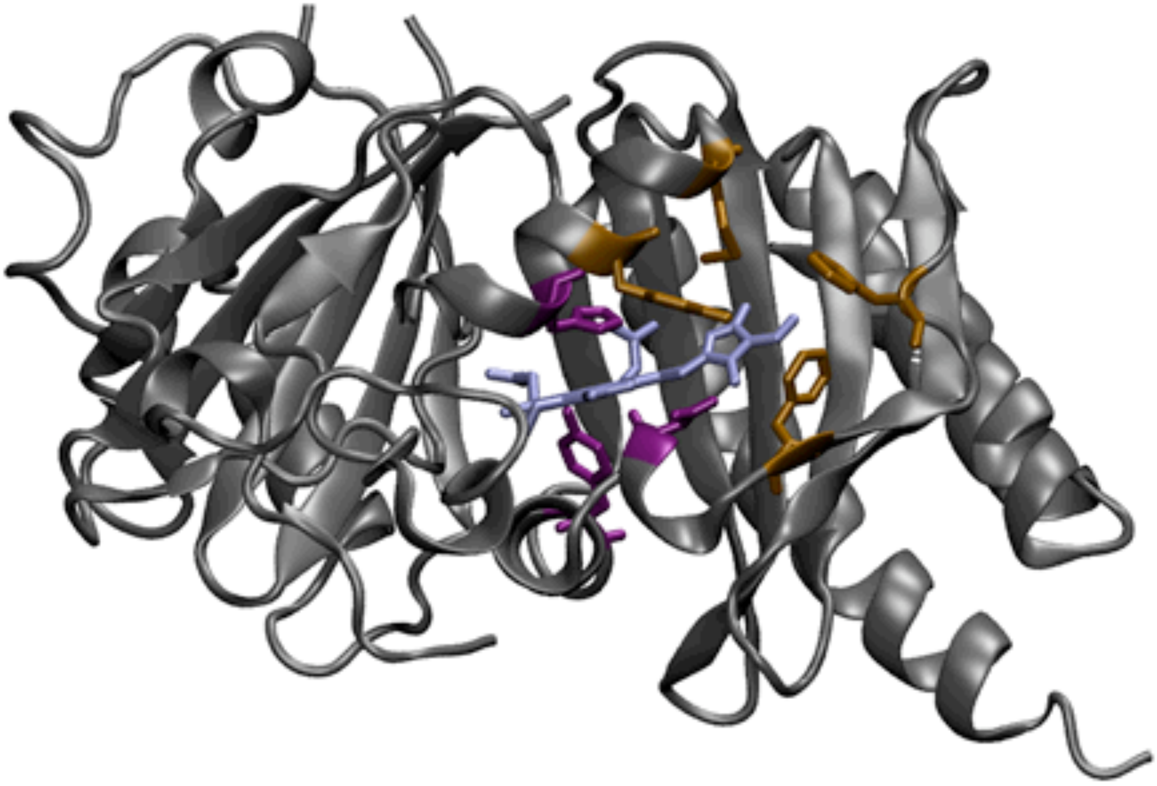

Additional information:

1. The only mutable field that is currently never used by homolmapper is charge, which is the last two characters in an ATOM or HETATM record and is infrequently used. The

charge field in the PDB file should not be confused with charge scoring in homolmapper, which is written to the occupancy and/or B-factor fields.

3.6 Using a PSSM instead of an alignment

Example directory: ubiquitin
Help flag: --alignments, --scoring

Background: The preparation of an MSA can be very time-consuming. While the structural context provided by homolmapper can be useful in evaluating an MSA, some users may well wish for a faster alternative. It is possible for homolmapper to project the PSSM (position-specific scoring matrix) generated by iterative PSI-BLAST searches onto a structure as an alternative to an MSA.

Preparing an appropriate PSSM: There are several ways to generate a PSSM. However, the most familiar PSSM today is that used by PSI-BLAST (Altschul *et al.,* NAR **25:** 3389) in iterative searching with a single query sequence. Such matrices can be obtained from web-based PSI-BLAST searches, but these matrices are encoded matrices for use in PSI-BLAST itself and are not currently supported by homolmapper. PSI-BLAST can also be run locally with a local database. Such runs can produce human-readable PSSM files with the "-Q <filename>" option. These files are properly formatted for homolmapper.

Using a PSSM: To be used within homolmapper, a PSSM file should have the extension '.pssm' and must be of the appropriate format. The filename is simply supplied on the command line as though it were an alignment file. Homolmapper will load the file and match the sequence to the structure as usual. However, there is no equivalent to a reference sequence; the matched sequence is always the query sequence and is always the reference.

Using --occ= and --B= with PSSM inputs: The PSSM inputs for --occ= and --B= are somewhat different than those for scoring an MSA. The scoring class is always set to “PSSM” to reflect this. The available scoring types are “query” (reporting the frequency of occurrence of the query residue), “charge” (calculates net charge with a ±1 scheme from the matrix of weighted observed percentages), “degen” (calculates observed percentages for a single user-defined set from the observed-% matrix), “sloppy” (assigns the query residue to a user-defined amino acid set and calculates the observed percentages for that set from the observed-% matrix), and “vector” (which considers each row of the PSSM as a 20-dimensional vector and reports various parameters about that vector and the query sequence).

Scoring with “charge” is quite similar to MSA charge or scharge scoring (Chapter 2). Similarly, degen or sloppy scoring on a PSSM is similar to the MSA equivalent and uses the same inputs (so --B=degen.+ will still mean --degen.KR, etc.). However, both “query” and “vector” scoring require unique arguments.

Query scoring: Specifying “query” as the PSSM scoring type will report the value associated with the query residue at each position to the output field. This can be applied to either the PSSM (specified by “log”) or the matrix of observed percentages (specified by “percent”),. One of these matrices should be supplied as an argument following “query” and separated by a ‘.’ character. Thus, to get the PSSM log-odds value for the query residue at each position in occupancy, one would specify --occ=query.log (which is the default setting if no --occ= or --B= values are supplied).

Vector scoring: In vector scoring, each row of the PSSM is considered to be a vector in a 20-dimensional space. Therefore, each residue in the query sequence is associated with a vector. The length of the vector at each position can be specified by supplying “length” as an argument after “vector” (separated by the usual ‘.’ character). One could view the query sequence as the one-dimensional projection of a 20-dimensional unit vector; the query vector would then be that unit vector normalized for the length of the corresponding PSSM vector. The distance between the endpoint of the query vector and

the endpoint of the PSSM vector is requested by “distance” as an argument, while the angle between these two vectors is requested by “angle” (one can also specify “polar” as a scoring type to get --occ=vector.distance --B=vector.angle, or the difference between the two vectors in polar coordinates). The angle between the two vectors is an assessment of the conservation of the query sequence (--B=vector.angle is the default).

Using --Seg= with PSSM inputs: SegID is used to report information on the PSSM itself: information per position, requested with --Seg=info, or relative weight versus pseudocounts, requested with --Seg=rel_wts; there is no default setting.

The element field in PSSM inputs: Assuming that the PSSM is successfully loaded and matched, homolmapper will report the consensus residue in the weighted observed-percentages matrix to the element field automatically. The consensus residue is chosen from this matrix rather than the log-odds matrix because this matrix is reported with higher precision.

Example:

1. The settings in pssm.hmset will project a PSSM onto 1BT0, which is the structure for a ubiquitin-like protein from *Arabidopsis.* Here are views of the log-odds for the query residue (left) and of information per position (right):

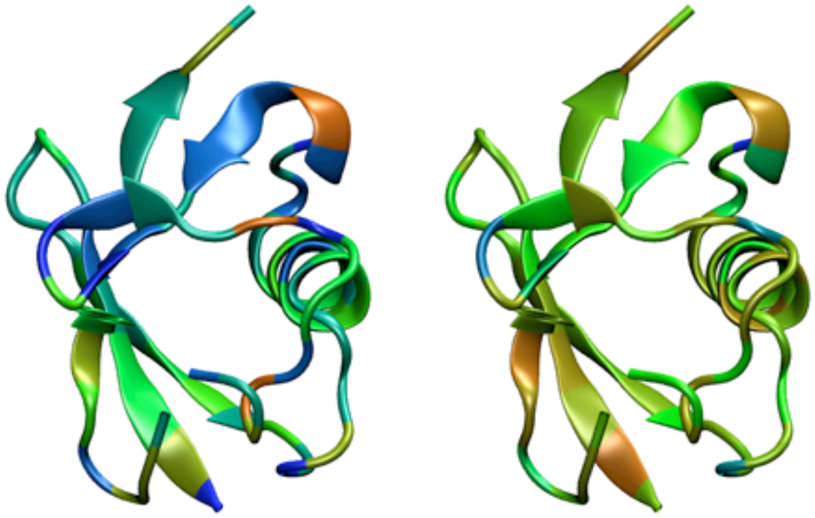

The PSSM was generated by searching the database of *Saccharomyces cerevisiae* protein sequences with the sequence of 1BT0.

Additional information:

1. User comments can be incorporated into the input PSSM file as comment lines starting with a '#' character. Such lines should not be placed within the matrix.

2. It is not possible to punch an alignment file or consensus sequence with a PSSM.

3.7 Mutual information analysis

Example directory: HO

Help flag: --scoring (also example.JE.mtx in the HO directory)

Background: Variable but coevolving residues in alignments can be detected by mutual information analysis (for an example, see Gloor *et al., Biochemistry* **44:** 7156). The mutual information for two positions can be defined as $H_i + H_j - H_{ij}$, where H_i and H_j are the Shannon entropies for the two individual positions and H_{ij} is the joint entropy. This is a measure of how much the identity of one of these two residues influences the identity of the other which is particularly applicable when conservation is low (i.e., when the single-position joint entropies are high).

To perform such analysis, it is necessary to calculate joint entropies for each pair of positions in the alignment. Mutual-information runs are therefore the most time-consuming analyses performed by homolmapper, because of the time involved in construction of the joint-entropy matrix. However, once a joint-entropy matrix for a given structure and MSA has been constructed, it can be punched for much faster reruns.

Implementation: Mutual-information runs cannot be performed with any other analysis, because results will be written to occupancy, B-factor, SegID, and element. The matching process proceeds normally and is also required for reruns, so that homolmapper can ensure that the calculated matrix will actually fit the inputs. Scoring in an MI run begins with an entropy-scoring call (Chapter 2) to calculate the single-position entropies.

After the single-position entropies are calculated, the joint-entropy matrix is calculated by summing over all possible pairs of outcomes (including gaps) for all pairs of positions in the alignment where the structure sequence has no gaps. This step is slow; for example, with the phytochrome example, constructing the joint-entropy matrix takes ~50 minutes on a single 1 GHz G4 processor, while the rest of the run takes about 15 seconds. The joint-entropy matrix then has single-position entropies summed in to give a matrix of raw mutual information values. These values can be used as-is or normalized.

Two normalization schemes are available: the raw mutual-information scores can be divided by the joint entropies at each position, or they can be divided by the sum of the two single-position entropies, which results in a quantity formally known as the redundancy in information theory. Finally, after normalization, the mean and standard deviation for the entire matrix are calculated and used to convert the normalized scores into Z-scores. The matrix of Z-scores is then used to find significant interactions to report to the output file.

Controlling mutual-information runs: Mutual information analysis is requested by supplying one of the mutual-information keywords in the --occ= or --B= lists. The relevant keywords are 'mutual' for mutual information, 'raw_MI' for unnormalized mutual information, and 'redundancy' for redundancy (e.g., --B=mutual on the command line).

The matrices used in mutual-information scoring can be punched for later use or for analysis with other software. This process is controlled by the --punch_matrix= flag, which can take 'JE' (for the joint-entropy matrix), 'MI' (for the mutual-information matrix), 'Z' (for the final matrix of Z-scores), 'all' (for all 3), or 'none' as arguments. The joint-entropy matrix is the only one that can currently be used in reruns. The default is to punch this matrix only.

Analysis of the final Z-scores uses a significance threshold. The default value is 5, meaning that the mutual information between two residues must be 5 standard deviations above the mean to be considered significant. Residues with no interactions above this value will be indicated by a value of 'nast' in SegID, meaning <u>n</u>othing <u>a</u>bove <u>s</u>ignificance <u>t</u>hreshold. The cutoff value itself can be set by the --mutual_Z= flag; reducing this value below 3 will trigger a warning message.

Results from mutual information scoring: Mutual information scoring potentially generates far more information than can be projected onto a single output PDB file. For example, if six residues are tightly linked, one would ideally like each residue to have the residue numbers of the other five, but there are only three output fields that can take

residue numbers greater than 99 within the PDB format without affecting the spatial and chemical information. The actual scores themselves thus would not be reported at all. As a partial solution to this conundrum, homolmapper always reports the highest Z-score to B-factor. For residues with significant interactions, three other parameters are reported: the residue associated with the maximum Z-score (SegID), how many residues have Z-scores above the cutoff for that residue (element), and the sum of all of those residue numbers, including the residue being described (occupancy). For residues with no Z-score above the cutoff, occupancy and element are reported as 0 and SegID is reported as ‘nast’ as described above.

Two interacting residue numbers are explicitly defined by this reporting scheme. The summed residue numbers (occupancy) and the knowledge of how many numbers are being summed (element) can be used to unambiguously identify three coevolving residues, which is the same number one could usually define with three output fields; the advantage with three residues is that this scheme uses the narrow element field to get there. An arbitrarily large set of coevolving residues will all have the same occupancy value in this scheme as long as they are all linked only to each other; therefore, identical occupancy and element fields are a sign that residues may be in the same group (although this is not guaranteed). Moreover, the use of the element field to indicate how many residues are linked to each residue makes it possible to know when the scores are precisely defined or under-defined by this reporting scheme.

How to save time: It is possible to perform a time-consuming mutual-information run and wind up with no residues interacting above the threshold. Of course, this may actually be an informative result. However, it can also be worthwhile to repeat the run with a lower value of the --mutual_Z= cutoff. Such reruns can also be performed with higher values of --mutual_Z= to deconvolute a large group of significant residues or to change the normalization technique. Fortunately, it is possible to use a previously calculated joint-entropy matrix to perform such repeats with much less time commitment.

To perform a rerun, the filename of the matrix to be used is supplied on the command line. Such files must end in '.mtx' (typically in '.JE.mtx' unless the file has been renamed). It is necessary to supply a structure and an alignment, but it is not necessary to specify the --occ= or --B= lists. A different normalization than the default must be explicitly requested if so desired. A value of --mutual_Z= other than the default value of 5 must also be supplied explicitly. Reruns will perform the usual matching process so that the loaded matrix can be verified. The matrix is loaded *in lieu* of the joint-entropy matrix calculation, and subsequent steps proceed normally.

Examples:

1. The settings in default_MI.hmset will perform mutual information scoring with default settings (MI normalization and a cutoff of 5). This process takes ~5 minutes on a 1 GHz G4 processor; while most computers will be faster than this, it will still take a while. Process reports will be shown with --verbose=2 or higher, or with --timing. Only the joint-entropy matrix is punched. At this cutoff, most residues will receive occupancy 0, element

0 (not O for oxygen), and SegID ‘nast’ to indicate there is nothing significant. However, two residues are detected: residues 136 and 140. The alpha carbons of residues 135 and 136 are shown here to illustrate the scheme used to report the results:

```
ATOM   1039  CA  THR A 135      50.593  21.096-120.017  0.00  3.43      nast 0
ATOM   1046  CA  ARG A 136      52.570  19.735-122.972 276.0  5.29      140  2
```

Thr135 is a non-interacting residue; note that the B-factor still reports the highest Z-score. For Arg136, occupancy is 276.0, B-factor reports a maximum Z-score of 5.29, and SegID indicates that this score is associated with residue 140. The element field indicates that two residues contribute to the score reported in occupancy (residue 140 and residue 136 itself, giving 136+140 = 276 for occupancy). Here is the alpha carbon for residue 140:

```
ATOM   1081  CA  ASP A 140      49.620  18.339-128.282 276.0  5.29      136  2
```

The occupancy, B-factor, and element fields are identical to those of residue 136, as expected for a mutually interacting pair like these two residues. The SegID now indicates that the highest score for residue 140 is associated with residue 136.

Here is the structure, with residues 136 and 140 in space-filling representation. They are in direct contact; in this structure, they are apparently forming a buried salt bridge. Interestingly, this Arg-Asp pair is found in only 48% of the sequences in the alignment. Arg136 can also be Val, Ile, Leu, Met, Gln, or Cys, while Asp140 can be Leu, Met, Ile, His, Ser, or Ala.

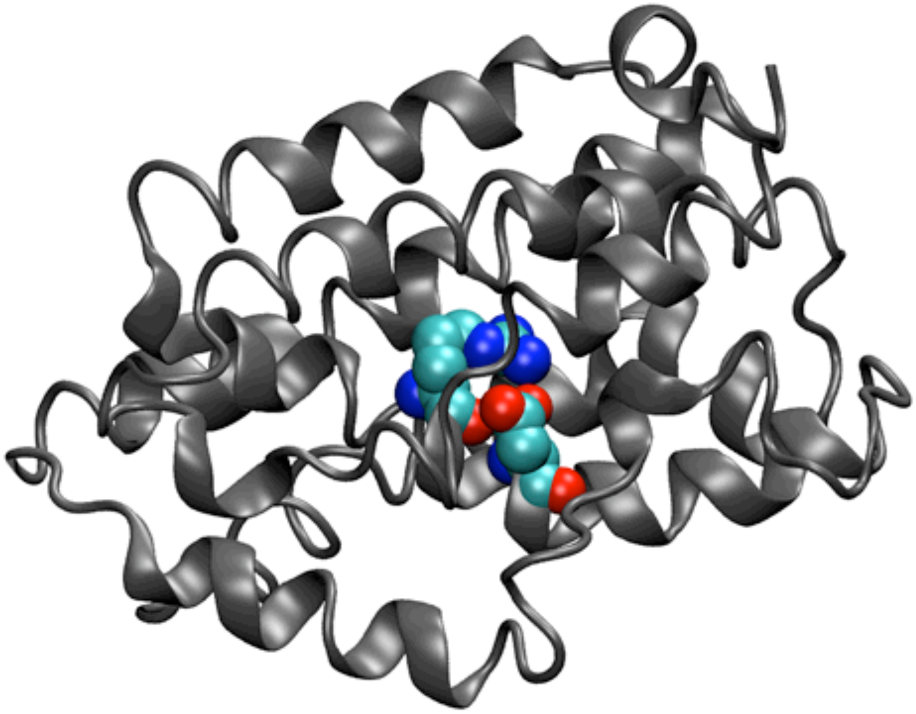

One could examine all residues with a lower maximum Z-score (i.e. lower B-factor) to see whether a lower cutoff will detect more interacting positions. Here are all residues having maximum Z-score ≥ 3.75 shown in space-filling representation:

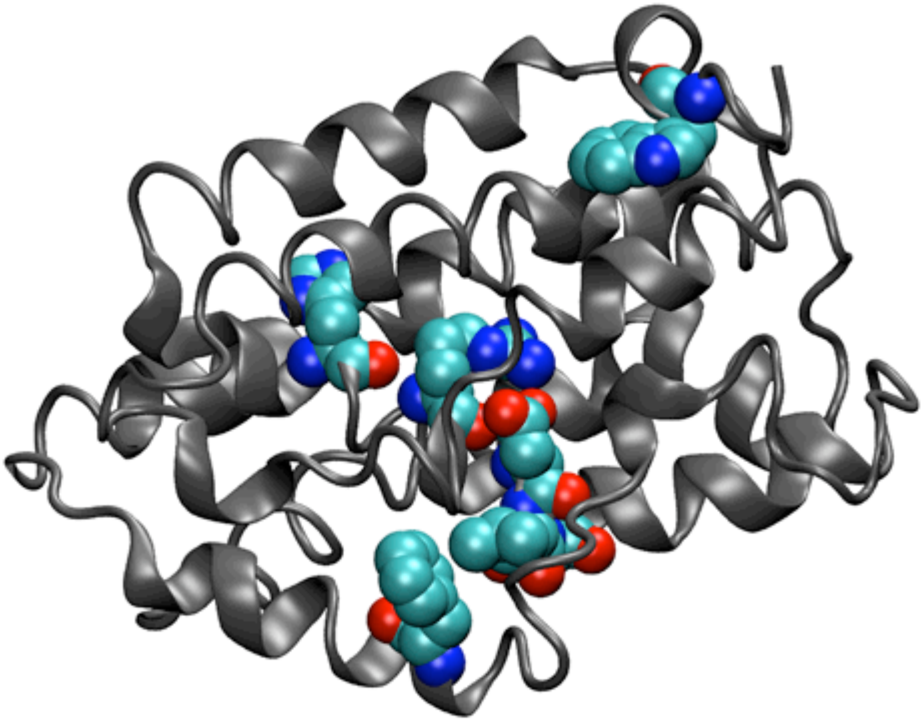

2. To map the interactions seen with the lower Z-score cutoff of 3.75, rerun375.hmset uses the sample matrix file example.JE.mtx to rerun this analysis. Here are alpha carbons for residues 135, 136, and 140:

```
ATOM   1039  CA  THR A 135      50.593  21.096-120.017  0.00  3.43      nast 0
ATOM   1046  CA  ARG A 136      52.570  19.735-122.972 586.0  5.29      140  4
ATOM   1081  CA  ASP A 140      49.620  18.339-128.282 549.0  5.29      136  4
```

Thr135 is still not interacting significantly, as expected based on its highest Z-score. The other two residues are now interacting as part of four-residue sets rather than two-residue sets (element field), but their highest interaction is still with each other (B-factor and SegID). However, their occupancy values are different, indicating that the sums of the residue numbers in the two sets are different. Therefore, the residue numbers in the two sets are different. Here are all alpha carbons for the residues detected at this level:

```
ATOM    761  CA  TRP A 101      70.995  19.645-136.839 243.0  3.95      142  2
ATOM   1012  CA  HIS A 132      54.494  19.800-116.932 408.0  4.37      136  3
ATOM   1046  CA  ARG A 136      52.570  19.735-122.972 586.0  5.29      140  4
ATOM   1081  CA  ASP A 140      49.620  18.339-128.282 549.0  5.29      136  4
ATOM   1089  CA  LEU A 141      46.520  16.133-128.565 281.0  3.96      140  2
ATOM   1097  CA  SER A 142      44.221  19.154-128.556 243.0  3.95      101  2
ATOM   1359  CA  PHE A 178      43.158   8.888-124.685 314.0  3.78      136  2
```

Examination of SegID shows that two new residues are most tightly linked to residue 136: His132 and Phe178. One new residue is most tightly linked to residue 140: Leu141. The element field shows that Phe178 and Leu141 are only linked to residues 136 and 140, respectively. His132 is linked to two other residues, with a sum of 408. 132+136 = 268, so the third residue can be easily identified by 408-268 as residue 140. Thus, the four-residue set for residue 136 is residues 132, 136, 140, and 178; that for residue 140 is residues 132, 136, 140, and 141.

The other residues detected at this threshold are residues 101 and 142. These two residues are most closely associated with each other (SegID) and have identical occupancy values, indicating they are part of identical sets. The occupancy values are both 243, indicating they are a mutually informative pair. Here are all of the detected residues, colored by occupancy on a continuous scale from red (243) to blue(586):

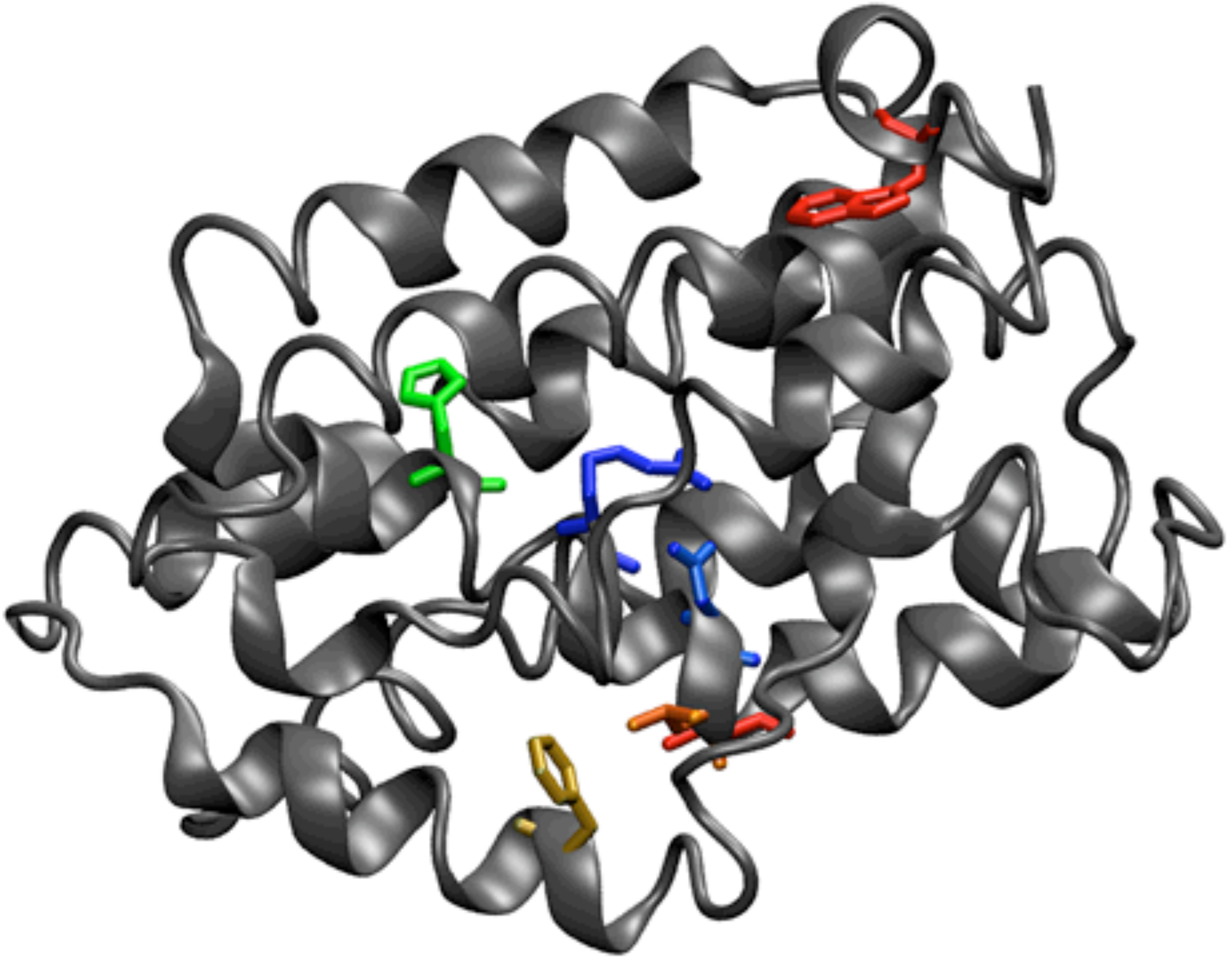

By analyzing the results of the two runs, one can deconvolute the occupancy values. For example, residues 136 and 140 (both blue) are both interacting with His132 (green). An interaction diagram of all relationships would look like this:

Interaction diagram (3.75 threshold)

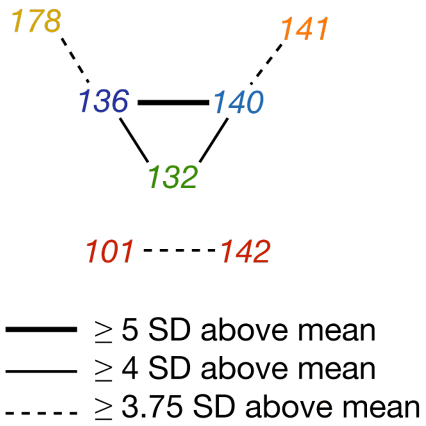

The header information generated by homolmapper in mutual-information runs provides a quick description of the various parameters. Here is the header for the rerun:

```
REMARK  This file generated by   homolmapper version 16.7.4
REMARK  using 1DVE.pdb, standard.aln      example.JE.mtx
REMARK  standard.aln: 75 sequences. Matched: ho1rat (214 aa).
REMARK  occupancy: MI   (sum of significant-residues group)
REMARK  B-factor: MI    (maximum observed Z-score)
REMARK  SegID:  (MI: residue # with highest significant Z-score)
REMARK  MI-matrix statistics: mean,  0.22; SD,  0.10
REMARK  MI normalization: mutual        Z-score cutoff: 3.75
REMARK  Thu Mar  1 21:31:37 2007        Runtime: 2.95 sec.
REMARK
REMARK
REMARK  Element: size of MI group
```

The occupancy, B-factor, and SegID descriptions indicate ‘MI’ followed by the parameter reported to each field. Instead of scoring statistics for occupancy and B-factor, statistics are presented for the normalized matrix (prior to conversion to Z-scores), along with what type of normalization was used and the significance threshold (Z-score cutoff). The last line of the header provides a description of the information reported in the element field in mutual-information scoring.

Additional information:

1. User-defined amino acid sets are not compatible with mutual information scoring.

2. Expanded amino acid sets are compatible with mutual information scoring.

3. Joint-entropy matrices cannot be punched on reruns, because --rerun= implies that such a matrix has already been punched.

4. Like --punch= settings, --punch_matrix= settings are not replicated in .hmset files. They must therefore be explicitly added to the command line.

5. Mutual information scoring implies --zero (that is, unmatched and unscorable residues have occupancy and B-factor set to 0 and SegID set to a blank string of 4 spaces). In mutual information runs, the element field will also be blanked for such residues.

6. In addition to lowering the value of --mutual_Z= in reruns, it is equally valid to raise the value. This can be useful in deconvoluting larger residue sets.

7. Interested users may wish to consult the results of Gloor and co-workers (*Biochemistry* **44:** 7156) for a general examination of mutual information as applied to coevolving positions within an MSA and for a good description of the caution required in preparing rigorous alignments for this purpose. The implementation of mutual information scoring in homolmapper owes a considerable debt to this paper, but no blame for the encoding of results in the output file may be attached to the authors.

8. The .mtx format has two comment lines at the beginning of the matrix which contain information about the matrix itself. These should not be edited or removed if the matrix is to be used in reruns. Both start with a '#' character. There is also a '#END' line at the end of the matrix. Users can add comment lines starting with '#' characters at the end of the file. The rest of the file is the actual floating-point numbers of the matrix itself.

Chapter 4: Analyzing a subfamily

Overview: Protein families usually can be divided into subfamilies on the basis of primary sequence, catalytic function, or both. Analysis of subfamilies to detect differences with functional implications is thus a frequent goal of structure/function studies. Homolmapper incorporates a number of tools for comparing a subfamily to the rest of the alignment. It also incorporates a variety of techniques for defining a subfamily either on the basis of sequence names or on the basis of the sequences themselves.

4.1 Defining a subfamily by name (explicit definition)

Example directory: phytochrome
Help flag: --subfamilies (also example.sfam)

Background: Homolmapper currently handles a single subfamily definition and compares the subfamily to the entire MSA. The structure need not belong to the subfamily, and homolmapper normally will apply a single scoring scheme to the subfamily (in occupancy) and to the entire MSA (in B). Subfamilies can be defined via three different approaches: explicit definition, implicit definition, and definition by regular expression (regex definition). These approaches permit definition of subsets of sequences based on the names of the sequences and/or the actual sequences.

The choice of definition method is largely driven by the alignment and the sequence names. If a subfamily can be defined by the nature of a few key residues, implicit

definition works well and is quite quick. If only one residue is being used, supplying an implicit definition via --sfam= is the fastest and easiest way to define a subfamily. If the names in the subfamily are well organized, regex definitions provide a means of using that order to get the desired results. The best control over membership comes with explicit definition, but this requires the most work on the part of the user.

Explicit definition of subfamilies: The conceptually easiest way to define subfamilies is simply to list all the sequence names that should be in the subfamily. This can be done by supplying a text file ending with '.sfam' that contains the requested names (case sensitive). The .sfam format simply lists names (1 or more per line, separated by whitespace). Anything after a '#' sign is considered a comment and ignored. Two lines in such a file could look like this:

```
drbphp          # structure sequence
atBphP1 atBphP3
```

One can also enter such information via the --sfam= flag. With this approach, the above lines would be represented as --sfam=drbphp.atBphP1.atBphP3 ('.' is used as a separator with the --sfam= flag). Note that the --sfam= flag will not work properly if the sequence names themselves have '.' characters in them. If a name is not found in the MSA, a warning is triggered and the spurious name is ignored.

Scoring with subfamilies: Homolmapper can use any of the scoring schemes for subfamilies except for range scoring. Currently, homolmapper will report scores for sequences that are in the subfamily to occupancy and scores for all sequences to B. There are two exceptions to this procedure. If "sfdiff" is included in the --occ= or --B=

scoring setup, the B field will instead report the default occupancy score minus the default B score. This can highlight regions that differ, depending on the scoring scheme. The second exception is when subfamily-specific residues are being evaluated.

Example:

1. explicit.hmset illustrates a default subfamily run (% identity scoring) using explicit definitions. The subfamily contains the structure sequence. 32 sequences are in the subfamily. Occupancy reports identity within the subfamily (left), while B factor reports identity for the entire MSA (right).

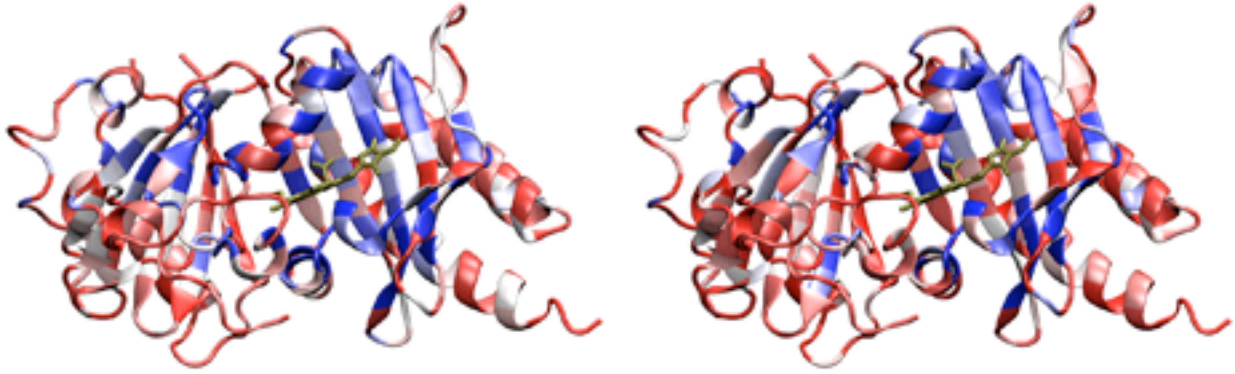

The subfamily exhibits higher conservation, especially in the central beta sheets. Among other residues, amino acids 24 and 216 in the output PDB are more conserved in the subfamily than in the entire MSA, while residues 36 and 172 are globally conserved. The alpha carbons for each of those residues are shown below:

```
ATOM    158  CA  CYS A  24      29.193  32.903  17.364 96.88 36.07           C
ATOM   1564  CA  TYR A 216      27.638  48.545  19.499 93.75 36.07           C
ATOM    251  CA  GLN A  36      28.607  41.874  36.677 100.0 95.90           C
ATOM   1222  CA  ARG A 172      46.728  49.401  26.825 96.88 99.18           C
```

Additional information:

1. The alnfilter utility (utils/scripts in the standard distribution) can extract a list of all the names in a MSA file as its default output. redirecting this list (directly or through a filter such as grep or sed) to a text file is thus a nice way to start making an explicit list, because one can then delete stuff rather than typing everything in.

2. If the last word on an explicit line in the .sfam file is 'NOT' or '!' (or if the last word in the --sfam= arguments is 'NOT' or '!' for an explicit list), the sequence names in that group will be explicitly left out of the subfamily. This is primarily useful when combined with other types of definition.

4.2 Defining a subfamily by sequence (implicit definition)

Example directory: phytochrome
Help flag: --subfamilies (also example.sfam)

Background: Meaningful subfamilies will normally be distinguishable by primary sequence elements. It is possible to define a subfamily by means of such elements; this is called implicit subfamily definition because the process of deciding which sequences are members is handled by homolmapper; the user provides one or more sequence criteria, and homolmapper does the rest. Implicit definitions can be supplied in .sfam files or via --sfam= and are identified by an '@' sign at the start of the definition:

```
@       drbphp  24      C       AND
@       cph1    216     F       NOT
```

After the identifier, the rest of the definition simply defines a single residue by sequence name, residue number, and residue name, and indicates whether this residue is required (AND, which is the default value and which can be abbreviated with '&') or forbidden (NOT, which can be abbreviated '!' or '^'). In this example, all those sequences which have a Cys residue at the position which aligns with residue 24 in drbphp and which lack a Phe at the position equivalent to 216 in cph1 will be members of the implicit subfamily, *assuming that drbphp has a Cys at residue 24 and cph1 has a Phe at 216.* If the name or residue information is inaccurate for a criterion, that criterion will be ignored. From the command line, --sfam=@.drbphp.24.C --sfam=@.cph1.216.F.NOT would supply the same definition (note that the 'AND' Boolean is optional, because it is the default setting).

Amino acid sets and implicit subfamilies: It is possible to list a degen definition instead of a single amino acid as part of an implicit subfamily definition. No extra inputs or flags are required. All wildcards are recognized. The wildcards meaning 'any residue' can therefore be used to select a subfamily on the basis of the presence or absence of amino acids, i.e. via gaps or insertions.

Examples:

1. implicit.hmset demonstrates a run with BLOSUM62 scoring. The subfamily is defined in implicit.sfam (this is the implicit example discussed above). 43 sequences are in the subfamily. The same 4 atoms are shown:

```
ATOM    158  CA  CYS A  24      29.193  32.903  17.364 100.0 33.61           C
ATOM   1564  CA  TYR A 216      27.638  48.545  19.499 100.0 61.94           C
ATOM    251  CA  GLN A  36      28.607  41.874  36.677 100.0 95.90           C
ATOM   1222  CA  ARG A 172      46.728  49.401  26.825 98.60 99.51           C
```

Notably, residue 216 is more conserved with a similarity scheme (as opposed to identity in the explicit example).

2. lacking.hmset illustrates a setup run (specified by --setup). The input files are evaluated and the subfamily definitions are applied, but no scoring takes place. A text file containing the names of the subfamily members is written (lacking.sfam, in this case). The defined subfamily contains 65 members. The subfamily definition uses a wildcard to define the subfamily as all sequences that have a residue (any residue) at a particular position in the alignment (--sfam=@.atphya.119.*). Note that adding a 'NOT' Boolean at the end would invert this criterion, thereby defining the subfamily as all sequences lacking a residue at this position (effectively defining the subfamily by a gap).

Additional information:

1. The --setup flag is a useful tool in building a complicated subfamily definition. It also can be useful in troubleshooting problems with the input files.

2. The final subfamily definition can also be written to a text file in explicit format (list of members' names as found in the MSA file) with the --punch=sfam flag. The result can be saved as documentation, used in a new run, or taken as a starting point for more editing.

4.3 Defining a subfamily by patterns in the names (regex definition)

Example directory: phytochrome

Help flag: --subfamilies (also example.sfam)

Background: The third means of defining subfamilies in homolmapper matches patterns in the sequence names via regular expression (regex) criteria. Such criteria are indicated by an initial '%' character. This is the most complicated way to define subfamilies, using a unique syntax in an attempt to provide something that will give a workable set of options for command lines on different platforms.

Homolmapper regex syntax: Each regex line is a single criterion, and all criteria are assembled to give a list of sequences that match all criteria (as is true for implicit definition). Each line looks something like these:

```
%       [Bb]ph[Pp]       AND
%       phy              NOT
%       ['4N'>           NOT     # exclude the atypical 4N sequence
```

Each line starts with a '%' character indicating a regex definition; the next word is the regex itself, and the last word is a Boolean indicating whether this criterion is required (AND, the default) or forbidden (NOT). AND and NOT can be abbreviated as for implicit definitions. Comments can be added to the ends of the lines if so desired (as usual, the '#' character indicates a comment). Regex definitions can also be supplied on the command line, though the entire flag must be enclosed in quotes to protect it from the shell. Therefore, the equivalent definition from the command line would be specified by three quoted flags as "--sfam=%.[Bb]ph[Pp].&" "--sfam=%.phy.!" "--sfam=%.['4N'>.^" (note that quotes should not be present in .sfam files).

The regex definitions in this example show the basic idea behind this syntax. Text can be within square brackets [ ] or not; <] or [> are used to indicate the left and right ends of the

name, respectively. Text that is outside such groupings is considered to be an exact, required fragment. Text that is inside such groupings can have several meanings: in the first line, [Bb] means that ‘B’ or ‘b’ are permitted in that position, and [Pp] is similar. This line would thus permit BphP, bphP, Bphp, and bphp, but not aphP or PphB. The next line simply matches the fragment ‘phy’ (and excludes all such matches, because the Boolean is NOT). The third line requires that the right-hand end of the name exactly match 4N, because the single quotes indicate an exact fragment inside the brackets (and again any matches are excluded by the Boolean). Thus, characters inside a grouping are considered to be possible valid characters in a larger context unless they are in single quotes, in which case they are an exact phrase, or unless they are certain other characters (metacharacters, for those used to regular expressions).

It is possible to indicate that a list of characters is disallowed at a position by adding a ‘!’ or ‘^’ character to the grouping. Thus, [Bb]ph permits bph or Bph, while [^Bb] permits aph, Dph, and so forth. It is thus possible to restate the third line in the example as this:

```
%      [^'4N'>
```

Here, the Boolean is reversed and the token ‘4N’ is now forbidden rather than required, so the effect is the same. Running this by itself on the 122-member MSA files in the phytochrome directory of the tutorial files yields a working subfamily of 121 sequences.

It is also possible to use the ‘@’ or ‘*’ characters as wildcards, either by themselves or with a subsequent number indicating how many characters can be skipped. However,

these wildcards are interpreted literally if enclosed in single quotes. Using the MSA files in this example, 6 sequences are chosen by either of the following definitions:

```
%       phP[*1]N
%       ph[@2]N
```

The ‘=’ sign can also be used to indicate that any single numeric character is allowed. Note however that ‘=’ will mean a literal equal sign if placed within single quotes inside a grouping (like other wildcards or metacharacters). The line below defines a working subfamily of 62 members in corrected.aln:

```
%     [=>
```

Combining definition methods: If both implicit and regex definitions are supplied, all implicit definitions will be evaluated for all sequences to generate a list of members, and all regex definitions will be evaluated for all sequences to generate another list. The names that are present in both of those lists will then be considered members of the subfamily by default (Boolean AND). However, it is possible to use the --sfOR flag to change this to a Boolean OR; that is, adding --sfOR will make any sequence that passes either the implicit tests or the regex tests a member of the subfamily.

If explicit definitions are present, those sequences will be added to the subfamily regardless of whether they match any other test, assuming the names are present in the MSA file. Addition of explicit sequences occurs after other tests have been evaluated. It is also possible to invert the subfamily definition after all evaluation has occurred (including

explicit definitions) with the --sfNOT flag, such that all sequences which formed the subfamily now are excluded and vice versa.

Examples:

1. regex.hmset demonstrates a run with --sfdiff using charge scoring. The subfamily contains 31 sequences and is defined by the first example above. The structure is within the subfamily and is used as the reference sequence. Residue 25 has a subfamily score of -0.78, which differs from the total score by 0.19 (in this case, the total score is less negative, indicating less conservation of the acidic Glu25 in charge scoring in the entire alignment relative to the subfamily). Arg172 has a subfamily score of +1, indicating high conservation of charge, and no difference is seen in the total score. The alpha carbons for these two residues are shown below:

```
ATOM    164  CA  GLU A  25      27.030  35.538  15.699 -0.79 -0.19           C
ATOM   1222  CA  ARG A 172      46.728  49.401  26.825  1.00  0.00           C
```

2. combined.hmset demonstrates a combination of all 3 definition methods to produce a subfamily with 36 members. In this case, the subfamily definition is supplied in the combo.sfam file, which has three significant lines:

```
@       drbphp  24      C             # 'AND' is assumed if no Boolean given.
%       FPH                    NOT
rpBphP4N
```

The first line (implicit type with starting ‘@’ character) selects all residues with a Cys aligned to Cys24 of drbphp. The second excludes all sequences with “FPH” in the name. No --sfOR is specified in the .hmset file, so only sequences that meet both criteria will pass through these two lines. The third line adds a single name explicitly. The resulting subfamily is written to combined.sfam. This is a --setup run, so no PDB file is generated.

Additional information:

1. More information about subfamily definition, and in particular about the regex syntax, can be found in the example.sfam file in the standard distribution (located in utils/examples/example.sfam).

2. The output .sfam files from the example --setup runs are included in the demo results.

4.4 Reference sequences and subfamilies

Example directory: phytochrome
Help flag: --subfamilies; --scoring

Background: As discussed in section 6, some scoring schemes are considered relative to a variable reference sequence. For subfamilies, this raises the question of what should be used as the reference, especially if the structure sequence is not in the subfamily or if a consensus sequence is being used as the reference. Gaps and insertions are always scored relative to the matched sequence (i.e. the sequence associated with the structure), because that is how they are defined.

Structure sequence as a reference: If the structure sequence is not in the subfamily but is the requested reference, the member of the subfamily with the fewest total gaps will be chosen as the reference sequence for the subfamily because it is assumed to have the best overlap with the matched sequence, so the least amount of information will be lost. The matched sequence will be used for scoring the entire alignment to B factor.

Specifying a reference sequence: Supplying a reference sequence name with use.NAME will cause NAME to be used as reference for both occupancy and B factor. This can be used to force use of the matched sequence as reference even if it is not in the subfamily if NAME matches the matched sequence.

Consensus reference sequences and subfamilies: The use of consensus reference sequences with subfamilies is ambiguous: should the subfamily be scored relative to the consensus sequence for the subfamily or the whole MSA? In homolmapper, the default behavior is to score the subfamily relative to its own consensus and the entire MSA relative to the global consensus sequence, with both consensus sequences written to SegID for inspection. However, this behavior can be controlled with the --global_cons and --inner_cons flags. The --global_cons flag will force both scores to be calculated relative to the consensus for the entire alignment, while the --inner_cons flag will force both scores to be calculated relative to the subfamily consensus.

Examples:

1. def_phy.hmset defines a subfamily that does not contain the structure sequence. The reference sequence is not set, so the structure sequence is chosen. However, it is not in the subfamily, so the subfamily member with the fewest gaps is chosen instead (acvphy3, here). Alpha carbons are shown for 4 residues:

```
ATOM    158  CA  CYS A  24      29.193  32.903  17.364 81.54 37.61           C
ATOM    164  CA  GLU A  25      27.030  35.538  15.699 100.0 16.24           C
ATOM    200  CA  ILE A  29      24.574  38.812  24.322 32.81 76.72           C
ATOM    208  CA  HIS A  30      21.893  41.401  25.110 100.0 30.17           C
```

Residues 24, 25, and 30 are conserved within the subfamily relative to the subfamily reference sequence, while residue 29 is not.

2. use_phy.hmset defines the same subfamily as above but explicitly sets the reference sequence to drbphp (the structure sequence) regardless of subfamily membership. Here are the same 4 atoms:

```
ATOM    158  CA  CYS A  24      29.193  32.903  17.364  0.00 37.61           C
ATOM    164  CA  GLU A  25      27.030  35.538  15.699  0.00 16.24           C
ATOM    200  CA  ILE A  29      24.574  38.812  24.322 64.06 76.72           C
ATOM    208  CA  HIS A  30      21.893  41.401  25.110  0.00 30.17           C
```

Here, residues 24, 25, and 30 in the subfamily never match those of the reference sequence (i.e., the structure), as shown by the occupancy scores. Residue 29 is somewhat conserved relative to the structure. The reference sequence chosen automatically is a good reference for residues 24, 25, and 30, but not for residue 29.

3. cons_phy.hmset defines the same subfamily and uses consensus sequences as references. Here are the same 4 atoms:

```
ATOM    158  CA  CYS A  24      29.193  32.903  17.364 81.54 45.30      A  A C
ATOM    164  CA  GLU A  25      27.030  35.538  15.699 100.0 55.56      Y  Y C
ATOM    200  CA  ILE A  29      24.574  38.812  24.322 64.06 76.72      I  I C
ATOM    208  CA  HIS A  30      21.893  41.401  25.110 100.0 61.21      Q  Q C
```

Here, all four residues are at least somewhat conserved relative to the subfamily consensus sequence (occupancy). All except Ile29 are less conserved relative to the consensus sequence for the entire MSA (B factor). The SegID has two residues listed here to show the subfamily consensus (left) and the global consensus (right).

For the above four atoms, the two consensus sequences are actually the same. In this example, the working subfamily contains slightly over half of the total sequences in the alignment, so conserved regions within this subfamily will tend to dominate the consensus sequence. However, there are regions where the two consensus sequences are different. Here are three additional alpha carbons from cons_phy.pdb:

```
ATOM     99  CA  GLY A  16      19.599  27.362  23.902 100.0 21.43      -  A C
ATOM    103  CA  PRO A  17      22.737  25.938  22.226 43.86 24.27      V  V C
ATOM    110  CA  GLU A  18      26.188  26.153  23.745 44.62 25.64      S  P C
```

Here, residue 16 in the structure is aligned with a gap in the subfamily, so the consensus sequence has a gap for this position. The occupancy score indicates that all members of the subfamily have a gap at this position. Residue 18 corresponds to a consensus Ser within the subfamily and a consensus Pro overall; the structure contains neither.

Additional information:

1. The double consensus sequence is only written to SegID when consensus references are used with subfamilies. --Seg=consensus works as usual.

4.5 Analyzing subfamily-specific residues

Example directory: phytochrome
Help flag: --subfamilies; --scoring

Background: Any physically meaningful subfamily is likely to be distinguished from the rest of the alignment at the level of primary sequence. That is, certain residues will be specifically conserved within the subfamily relative to the rest of the alignment. Finding

such residues can be a goal of homology analysis: for instance, one can define a subfamily on the basis of a few key residues and then look for others that might also be interesting. HomoImapper provides support for such searches.

Subfamily-specific residues: Given a subfamily definition, homolmapper can look for specified subfamily-specific residues. This is requested by adding “sfspec” to the scoring string, followed by a ‘.’ and a target residue or set of residues. For example, one might look for Cys residues specific to the subfamily defined in implicit.sfam in with this command line:

```
homolmapper 1ZTU.pdb corrected.aln implicit.sfam --occ=sfspec.C --out=fred
```

The output file (fred.pdb) will write scores of 0 or 1 to occupancy, with a score of 1 indicating a subfamily-specific residue.

Searching for subfamily-specific residues takes only a single output, so the other can be scored with any available scheme (or left alone). Whether the subfamily or the whole MSA is scored depends on whether the other request is reported in occupancy or B factor. It is not currently possible to combine sfspec with sfdiff or range scoring.

Setting tolerances for sfspec scoring: Detection of subfamily-specific residues boils down to finding positions where the subfamily has only a right residue and the other sequences have only a wrong residue. Of course, actual alignments may contain a few cases where things that should be in the subfamily nevertheless lack a certain residue, or where things that should not be in the subfamily nevertheless have a certain residue. To

allow detection in the presence of such noise, homolmapper provides two flags to set the tolerances for detection: --in_tol= and --out_tol= (for tolerance inside and outside the subfamily respectively). These both take integer arguments which are the number of sequences that can miss for either example. The default values are 1 and 2 respectively, meaning that 1 incorrect residue is tolerated in the subfamily and 2 correct residues are tolerated outside the subfamily. Here is the same example as above:

```
homolmapper 1ZTU.pdb corrected.aln implicit.sfam --occ=sfspec.C --out=fred
```

This implies --in_tol=1 and --out_tol=2, and the run locates a single position. No positions are located with --out_tol=0, because one sequence excluded by the subfamily definition nonetheless has a Cys at this position. Using explicit.sfam instead of implicit.sfam will locate no positions, because one group of sequences with a Cys at position 24 is absent from this definition and one sequence in the definition lacks this residue. Using this feature well requires thoughtful evaluation of the alignment and subfamily definition.

Scoring with --Seg=autospec: Using sfspec scoring requires the user to guess which amino acids might be of interest. A more objective approach would be to look for any such residues (or sets of residues). The --Seg=autospec option will report detected residues to SegID by the name of the residue (or up to the first four residues in a set). The occupancy and B-factor are left free for other outputs.

Examples:

1. spec_C.hmset searches for subfamily-specific Cys residues. The matched sequence is in the subfamily, and alpha carbons for all of its Cys residues are shown below.

```
ATOM    158  CA  CYS A  24      29.193  32.903  17.364  1.00  1.00           C
ATOM    665  CA  CYS A  93      11.946  57.714  24.744  1.00  0.00           C
ATOM   2102  CA  CYS A 289      41.141  49.505  26.447  1.00  0.00           C
```

2. spec_Y.hmset adds evaluation of subfamily-specific Tyr residues to the previous example. The Cys results are identical, and Tyr216 is the only Tyr located. Here is the structure, with all subfamily-specific hits detected shown in space-filling representation:

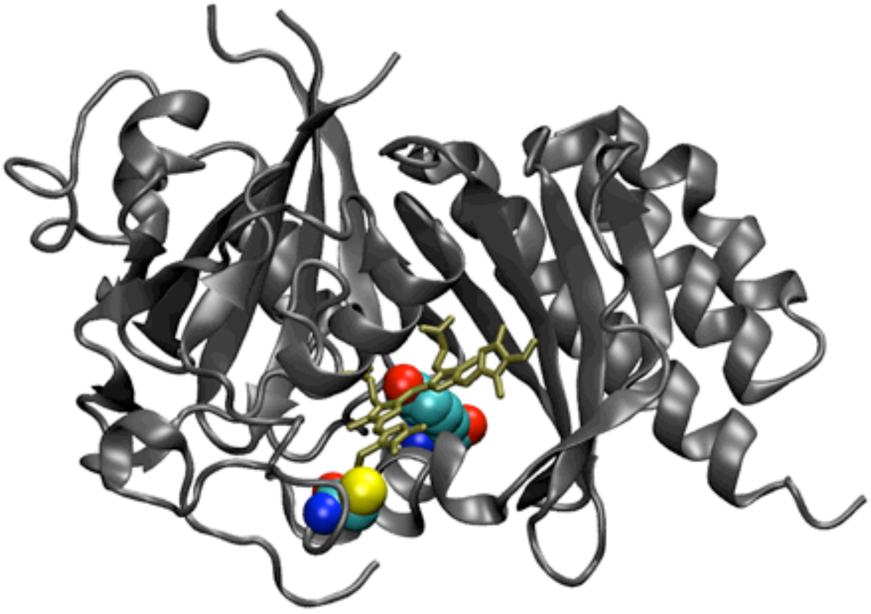

3. fastauto.hmset uses --Seg=autospec to look for all possible subfamily-specific residues with the standard 20 amino acids. This example uses default values for --occ= and --B=. The tolerances are each set to 4, which is rather permissive. One could perform a more rigorous analysis with lower values of --in_tol= and --out_tol=. This is especially important when working with small alignments or small subfamilies, because the total number of sequences is too low to be statistically valid. However, this approach permits candidate

residues for further study to be chosen with very small alignments, for example when the experimental behavior under study has only been characterized for a few proteins. Here is the result, with all detected residues shown in space-filling representation:

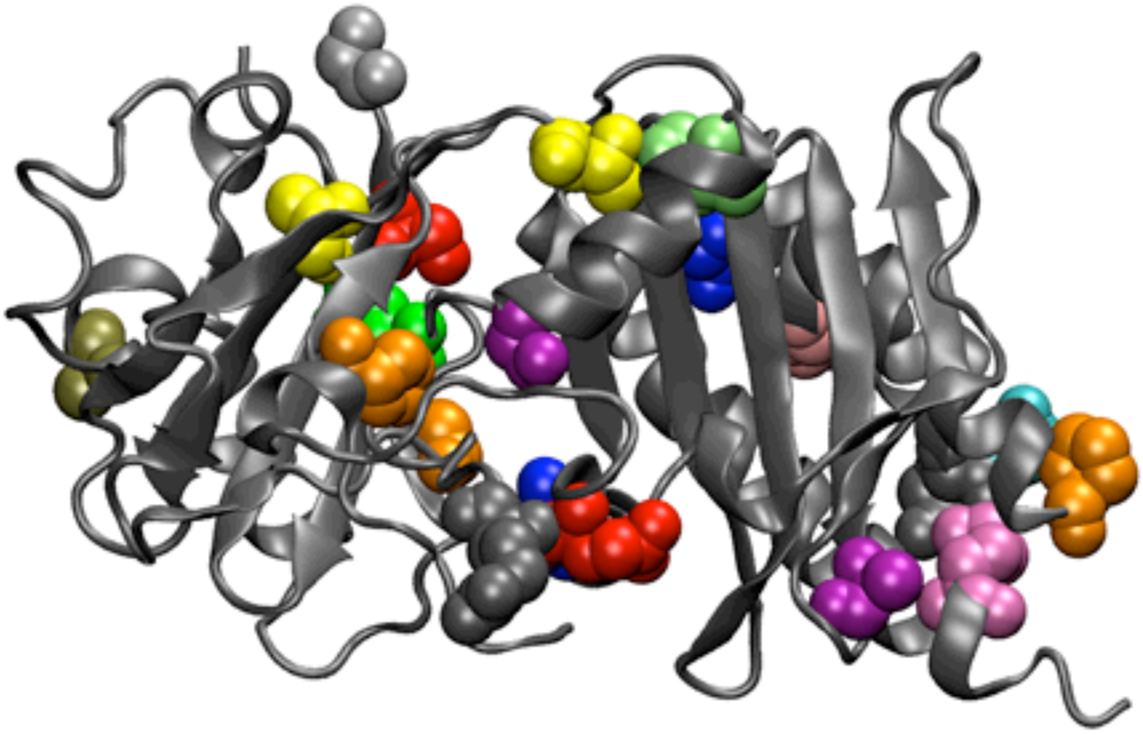

Additional information:

1. No reference sequence is used in sfspec scoring, and the structure sequence need not be in the subfamily for this scheme.

2. The structure will only display subfamily-specific residues that are aligned with a residue in the structure. However, a list of all hits not aligned with gaps in the matched sequence is included in the output header.

3. Residue sets can be used (including shorthand definitions). This can be useful in searching for subfamily-specific charges, for example.

Chapter 5: Scoring the unscored

Overview: HomoImapper scores the alignment and then matches the results to the structure. Most structures will contain atoms that cannot be scored because they are not represented in the alignment, such as crystallographic waters or ligands. HomoImapper normally ignores such atoms, but they can be minimally processed at need.

Additionally, either the structure or the alignment may contain residues that are not part of the canonical 20-residue set. An extreme example would be the selenocysteine residue, which can be considered a true 21st amino acid. HomoImapper provides the means for dealing with such cases accurately.

5.1 Handling unscored residues

Example directory: phytochrome
Help flag: --flags

Background: For many cases, there will only be a partial overlap between the structure and the alignment. 1ZTU is the 2.5Å crystal structure of two domains from the photosensory core of BphP from *Deinococcus radiodurans.* The sequence alignments contain the full sequences for 122 members of the phytochrome family, including bacterial, fungal, and plant examples. The structure is only a subset of the complete sequence in the MSA and has several missing residues that were not resolved during structure determination. Moreover, several residues in the C-terminal His tag were

resolved and are part of the final structure. Parts of the structure that cannot be scored are by default passed through to the final PDB file without any changes. This can make it hard to tell which parts have been scored: for example, in the default percent identity scoring in B factor, values will range between 0 and 100, comparable to the experimental values for this structure. Of course, actual B factors are per-atom properties that vary for different atoms within a residue, while homolmapper scores are per-residue properties that are constant for all atoms within a residue. However, homolmapper provides tools for handling the unscored portions of the structure.

Default handling of unscored portions of the structure: In reading a .pdb file, homolmapper divides the file line-by-line into two portions that will be included in the final file. These are scorable residues (i.e., those atoms whose ResName fields can be recognized as scorable amino acids) and non-scorable residues (ligands, crystallographic waters, etc.). Homolmapper will attempt to score all of the scorable residues; if one cannot be scored because it does not match the sequence in the alignment, it will be given a score of 0.0 (this is the exact score; a scored residue whose score is 0 will be given a score of 0.00, so that they can be distinguished by significant digits if need be).

Assigning zero scores to unscorable residues: Unscorable residues (e.g., crystallographic waters) will by default pass through unchanged. This behavior can be altered with the --zero flag, which will cause such atoms to get occupancy and B factor scores of 0.0, as though they were scorable residues that did not get scored. This can make it easier to find scored atoms and to get good ranges in visualization programs.

Default translation of nonstandard residues in the structure: In this example, the protein was solved with selenomethionine in place of methionine to permit easier phase determination. As a consequence, every Met in the MSA is matched to Mse in the .pdb. Mse is not a naturally occurring amino acid, so homolmapper must convert Mse to Met to match the residues and permit scoring. There are five such amino acid derivatives that are equated with their genetically encoded equivalents by default: Mse (selenomethionine, converted to Met), Sep (phosphoserine, converted to Ser), Ptr (phosphotyrosine, converted to Tyr), Tpo (phosphothreonine, converted to Thr), and Hyp (hydroxyproline, converted to Pro). This translation is suppressed by the --20aa flag, which will force homolmapper to only match with the canonical 20 amino acids.

Examples:

1. The typo.hmset file uses typo.aln, which contains a 1-residue mismatch at position 240 of DrBphP (drbphp in the MSA), here taken as a typo in the MSA. In corrected.hmset, the same settings are applied to corrected.aln, which fixes that mismatch. 11 lines are different between corrected.pdb and typo.pdb. Four of these are in the header, while the other seven are in Thr240, the mismatched residue (individual lines will be shown by diff or by linecomp --verbose). The alpha carbon of Thr240 looks like this in the PDB files:

```
ATOM   1752  CA  THR A 240       7.425  39.467  34.714  1.00 47.86           C
ATOM   1752  CA  THR A 240       7.425  39.467  34.714  0.0   0.0            C
ATOM   1752  CA  THR A 240       7.425  39.467  34.714 10.00  4.35           C
```

The first line is for the input file, 1ZTU.pdb. Occupancy is 1.00 and B factor is 47.86. The second line is with the incorrect alignment. Because this residue was scorable but not

matched, its occupancy and B factor have been set to 0.0. In the bottom line, the typo is corrected and the residue is given homolmapper scores.

The same comparison is shown for one carbon from the BLA residue of 1ZTU.pdb (biliverdin chromophore). In this case, all three lines are identical because the residue was unscorable.

```
HETATM 2422  CHB BLA A 328      38.019  41.686  20.160  1.00 33.06           C
HETATM 2422  CHB BLA A 328      38.019  41.686  20.160  1.00 33.06           C
HETATM 2422  CHB BLA A 328      38.019  41.686  20.160  1.00 33.06           C
```

Finally, the alpha carbon from Mse267 is shown for all three files. The two output files are identical in this case, because Mse was equated with Met. Note however that MSE is officially considered a HETATM; whether a given residue is ATOM or HETATM has no bearing on whether homolmapper will attempt to score a residue, because it uses the residue name.

```
HETATM 1955  CA  MSE A 267      45.400  37.952  26.614  1.00 37.66           C
HETATM 1955  CA  MSE A 267      45.400  37.952  26.614  0.00 97.54           C
HETATM 1955  CA  MSE A 267      45.400  37.952  26.614  0.00 97.54           C
```

2. The zero.hmset file uses corrected.aln with the --zero flag. This produces 78 mismatched lines in the output PDB file relative to corrected.hmset (assessed with linecomp).Here are the resulting lines for the three atoms shown above:

```
ATOM   1752  CA  THR A 240       7.425  39.467  34.714 10.00  4.35           C
HETATM 2422  CHB BLA A 328      38.019  41.686  20.160  0.0   0.0            C
HETATM 1955  CA  MSE A 267      45.400  37.952  26.614  0.00 97.54           C
```

The first and third lines have been scored. The second line differs from its equivalent in the first example because occupancy and B field have been given values of 0.0.

3. The strict.hmset file uses the corrected alignment with the --20aa flag. The three atoms described above are shown here for this case:

```
ATOM   1752  CA  THR A 240       7.425  39.467  34.714 10.00  4.35           C
HETATM 2422  CHB BLA A 328      38.019  41.686  20.160  1.00 33.06           C
HETATM 1955  CA  MSE A 267      45.400  37.952  26.614  1.00 37.66           C
```

Here, the first line has been scored as normal, but both the second and third lines are passed through, because MSE is no longer considered a valid three-letter code for a scorable amino acid and all Mse residues are therefore sent to the unscorable group. Examination of the various output PDB files demonstrates an unintended consequence of the --20aa flag: other scores change because the Mse residues are no longer matched. For example, His260 is no longer scored, because it is sandwiched between two Mse residues and is therefore in too small a fragment to be considered significant.

Additional information:

1. If only one of occupancy or B factor are scored, the other is by default unchanged. It can instead be set to 0.0 by using the --cross_zero flag.

2. The --zero flag does not alter the value of SegID of an unscorable atom by itself. Combining it with one of two flags (--Seg=numbering or --clobber_Seg) will set the SegID to ‘UNSC’ (unscored).

5.2 Handling nonstandard residues in the structure

Example directory: cutinase

Help flag: --noncanonical (also sample.aalib)

Background: Many proteins are post-translationally modified, covalently bound to ligands or inhibitors, or otherwise incorporate nonstandard residues as part of the polypeptide chain. By default, homolmapper is able to recognize 5 such residues and convert them to their genetically encoded equivalents (see above). However, there are many more such cases. Homolmapper thus incorporates a general solution to this problem: the user can supply an accessory file on the command line to provide translation information. The example uses this to correctly handle 2 diiodotyrosine residues found in a structure of acetylxylanesterase (PDB code 2AXE).

Working with .aalib files: The files used to import PDB translation information have the extension .aalib (for amino acid library). Any number of amino acids can be found in 1 file, and none of them need be present in the structure (it can therefore be convenient to simply use one such file and keep adding to it, unless the same 3-letter code needs to be equated with different 1-letter codes in different structures). Within a given .aalib, there are amino acid definitions and optional comments (the latter indicated by '#' characters as usual). Thus, one line of a .aalib file might look like this:

```
TYI   Y     # diiodoTyr (3,5 or di-ortho, found in 2AXE)
```

The first word is the actual 3-letter code used in a structure; the next is the 1-letter code to which it should be considered equivalent. Anything else on the line should be a comment; here, the amino acid corresponding to this 3-letter code is defined and an example is given (this line is taken from the sample .aalib file in the lib/ directory). When the .pdb file is read by homolmapper with this .aalib file imported, any TYI residue will be considered a Tyr for matching to the MSA.

The first word on the line formally need not be in 3-letter code, but it must match the residue name *as it appears in the .pdb file.* It will not be translated or converted in any way prior to assignment to a one-letter code for MSA analysis.

The --interpret= flag: As an alternative to the .aalib file, one can also supply handling information for a PDB residue with the --interpret= flag. This flag takes two arguments, separated by a '.' character: the PDB code to translate, and the one-letter code to which it should be equated. Thus, the example line above could also be entered on the command line as --interpret=TYI.Y.

Examples:

1. no_lib.hmset uses default residue definitions to match 205 residues of PpAXE to the structure. Neither TYI residue will be scored by default, because TYI is unrecognized. Writing the matched residue numbers to SegID with --Seg=numbering will show a gap in the matched sequence at each TYI residue in the output .pdb file; combining it with --zero will assign a SegID of UNSC to the TYI residues. Here are the TYI alpha carbons:

```
HETATM  222  CA  TYI    33      14.463  18.037  30.315  0.0   0.0       UNSC C
HETATM 1225  CA  TYI   177       5.250   6.390   1.454  0.0   0.0       UNSC C
```

2. yes_lib.hmset uses the library file 2AXE.aalib and gives a new file that scores both TYI residues as Tyr (giving 75% identity at TYI 33 and 100% at TYI 177). These are the same atoms with the library file enabled:

```
HETATM  222  CA  TYI    33      14.463  18.037  30.315  0.00 75.00      60   C
HETATM 1225  CA  TYI   177       5.250   6.390   1.454  0.00 100.0      204  C
```

Additional information:

1. A standard dictionary of 3-letter residue abbreviations can be found at the PDB web site; this should be followed when possible as good practice.

2. This approach will generally map a 3-letter code in the .pdb to a 1-letter code that may be found in the MSA file. While this could conceivably be used to redefine standard 3-letter codes, that is usually a bad idea, so attempting to do so will trigger an error. If this is actually intended, the error can be suppressed with the --rEdEF flag.

3. The --lib= flag can also be used to import multiple .aalib files from a single directory.

5.3 Handling nonstandard residues in the alignment

Example directory: phytochrome
Help flags: --noncanonical; --flags

Background: Protein sequence alignments can also contain nonstandard residues, for example in the case of sequencing ambiguity. For some scoring schemes, this is not necessarily a problem. However, the substitution matrices in homolmapper usually fail with such characters. It is therefore possible for homolmapper to convert nonstandard 1-letter codes in the MSA into one of the canonical 20 for matching and scoring.

Scoring with noncanonical 1-letter codes. The presence of a noncanonical residue in the alignment does not cause problems for gap or insertion scoring, which only consider the presence or absence of an amino acid. Identity scoring will simply consider a noncanonical residue as being nonidentical, and sloppy and degen scoring will consider

any undefined one-letter code as not belonging to any amino acid set. However, to score such residues with similarity matrices (either substitution matrices such as the BLOSUM or PAM series or physical ones such as charge), the --translate= flag is used to translate them into one of the standard 20 residues.

Translating noncanonical 1-letter codes. The presence of a nonstandard amino acid in a MSA could mean a number of things. For example, ‘X’ typically is used to indicate any amino acid, but the underlying data may actually permit an assignment. Alternately, subsequent data could resolve an ambiguity, or at least suggest a translation. When it is appropriate to convert a nonstandard residue in the MSA into one of the standard 20 for matching and scoring, the --translate=U.Z flag is used, where U represents the nonstandard code and Z represents the standard code to which to translate.

Example:

1. translate.hmset uses translate.aln, which contains an ‘X’ at position 223 of the ‘zmphya’ sequence. In this case, another isolate has a Cys at this position, which is also quite common in the close homologs in the sequence. The ‘X’ is therefore translated into a ‘C’ with --translate=X.C (last line in the .hmset file). This residue is aligned to residue 157 in the structure; its alpha carbon is shown here before scoring (top) and after scoring with translation (BLOSUM62 to B factor, middle).

```
ATOM   1108  CA  ALA A 157      44.373  58.196  16.258  1.00 49.11           C
ATOM   1108  CA  ALA A 157      44.373  58.196  16.258  1.00 -4.10           C
```

Additional information:

1. Translation of MSA characters is less generalizable than translation of nonstandard 3-letter codes in PDB files. Therefore, there is no equivalent of the .aalib file. A frequently needed translation can be stored in a .hmset file if so desired.

2. The --translate= syntax will translate *all* occurrences of a nonstandard 1-letter code in the alignment. Care must be taken to ensure that this is intended.

3. The --translate= flag should not be confused with the --interpret= flag, which is for handling residues in a PDB file rather than an alignment file. The names are chosen because the sequences in an alignment that contain noncanonical codes are more likely to reflect poor DNA sequence data. Thus, they may be experimentally ill-defined, and translating those codes into amino acids may reflect uncertainty at the DNA or RNA level, akin to the biological process of translation. The use of --interpret= for the PDB file reflects the fact that such residues are often of known chemical character, so it is a question of deciding how to interpret them in terms of the sequence as synthesized, which is more likely to be equivalent to the one in the alignment. Residues that are noncanonical in both structure and alignment are handled differently (see below).

5.4 Handling a 21st amino acid

Example directory: FDH
Help flag: --noncanonical

Background: It is sometimes necessary to translate non-standard characters in both the MSA and the .pdb. As an example, consider selenocysteine (3-letter code Cse). Cse

might be found in a structure because the protein was expressed in low-sulfur, high-selenium medium for MAD phasing, but certain proteins actually incorporate Cse naturally as a “21st amino acid.” This then poses a problem, because equating Cse to Cys for such a case would be incorrect. The example for this section is formate dehydrogenase H, which has a Cse residue in the active site (residue 140 in chain X). Simply pulling protein sequences out of Genbank (FDH.aln) gives nothing unusual; these residues are simply indicated as ‘C’ or sometimes ‘X’. However, a little research would indicate the presence of the nonstandard residue, as would reading the starting PDB header information. The residue in question is then replaced with a nonstandard 1-letter code in the MSA (here, U has been arbitrarily used in selFDH.aln) to give an alignment that indicates that the special residue is not one of the standard 20.

One could then run selFDH.aln with translation as above (e.g., --translate=U.V), but this will not score the Cse residue because the .pdb file is not being translated. Therefore, in this example, adding PDB translation via .aalib file is also necessary. val_trans.hmset uses the alternate.aalib file to equate Cse with Val. The resulting val_trans.pdb file scores Cse as Val, which happens to be the only other residue aligned to the troublesome Cse in the MSA file:

```
EcoFDH          NETNYVMQKFARAVIGTNNVDCCARVUHGPSVAGLHQSVGNGAMSNAINEIDNTDLVFVF
YpseudFDH       NEANYVMQKFARAAIGTNNIDCCARLUHGPSVAGLQQTLGNGAMSNSIGEIENTDCILVF
CdiffFDH        NEANYIMQKFMRATIGTNNVDHCAR-VHAPSVAGLAYSLGSGAMSNSIPEIENADVLFIF
MthermFDH       NEENYLLQKFARAVIGTQNVDHCARLUHGPSVAGLAKTFGSGAMTNSISDIEESSCIFII
pyroFDH         NEENYLIQKIAR-LLGTNNVDNCARLUHESSVHALKLTLGDGVQTNPYSDLERFGAIMIW
Clos.OhILAs     GVVKSIDNAFFNSYGGATLPK--GSLUWSAGIAAQKLDFG-KPLSHNPDDYLNAKTILIW
                .  : : : : .   *:   .  .     ..: .    .*    ::   :  .   :::
```

Thus, a percent identity score of 100 is given, but this procedure has translated both the nonstandard 3-letter code CSE (.pdb file) and the nonstandard 1-letter code U (.aln file) as Val. The correct solution is actually to consider Cse as a true 21st amino acid, which means that the set of allowed amino acids needs to be expanded.

Expanding the amino acid set: To expand the amino acid set, one must provide a .aalib file or --interpret= flag matching the special 3-letter code to a *nonstandard* 1-letter code (seleno.aalib in this example). This by itself will trigger an error, because the 1-letter code is nonexistent by default. One therefore uses the --expanded= flag to add the code to the amino acid list homolmapper uses to match 3-letter and 1-letter codes. In this example, the appropriate setting is --expanded=U because U is the only nonstandard code in the .aalib file (expanded.hmset has these settings and gives 83.3% identity for Cse, as expected). The --expanded= list must contain all nonstandard residues found in a given .aalib file. Translation of the same character with expansion (--translate=U.C --expanded=U) will have no effect: translation is applied after any expansion is performed, and is only performed on amino acids that are not present in the runtime amino acid set. This also means that --translate= cannot be used to redefine standard codes by accident.

Scoring with expanded residue sets: Expanded amino acid sets will work for scoring gaps, insertions, and identity without any extra work. However, the built-in similarity matrices in homolmapper use the canonical 20 amino acids to allow the user more

flexibility in handling exceptional residues. To score expanded cases using similarity, expanded matrices need to be prepared (Chapter 6).

Examples:

1. default.hmset will not score the Cse residue (because no translation is applied). Here is the resulting alpha carbon of the Cse residue (occupancy and B field of unscored atoms are set to 0.0 by --zero):

```
HETATM 1071  CA  CSE X 140      93.012  39.204  31.773  0.0   0.0           C
```

2. aln_trans.hmset also will not score the Cse residue, because only the MSA is translated. Here is the alpha carbon of the Cse residue:

```
HETATM 1071  CA  CSE X 140      93.012  39.204  31.773  0.0   0.0           C
```

3. val_trans.hmset will score the CSE residue at 100% identity, because both the MSA (U) and the .pdb (Cse) are converted to Val for evaluation. Again, the Cse alpha carbon:

```
HETATM 1071  CA  CSE X 140      93.012  39.204  31.773  0.00 100.0          C
```

4. expanded.hmset uses the seleno.aalib file to equate CSE with U and then expands the amino acid set with --expanded=U. These settings result in correct scoring of the Cse residue at 83.3% identity (5 out of 6 sequences in the above alignment). Here is the Cse alpha carbon:

```
HETATM 1071  CA  CSE X 140      93.012  39.204  31.773  0.00 83.33          C
```

Here are pictures of the Cse residue and surrounding amino acids in the starting structure (left, colored by atom type), valine translation (center, colored by identity), and

proper handling (right, colored by B factor again). Protein residues ≤ 3.5 Å from the Cse residue are shown.

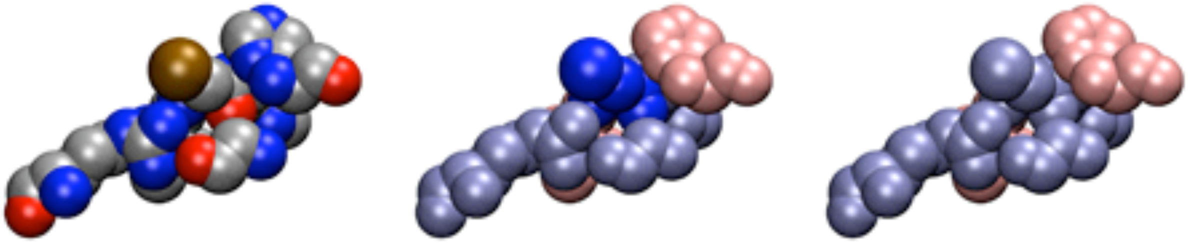

Additional information:

1. The PDB maintains a list of standard three-letter codes which should be followed when possible as good practice.

2. A similar approach is required for any amino acid derived from selenocysteine (such as seleninic acid) or for any other structure with an expanded amino acid set, such as a protein incorporating an unnatural amino acid via modified tRNA techniques or incorporating pyrrolysine (sometimes called the "22nd amino acid").

Chapter 6: Pushing the boundaries

Overview: The built-in scoring schemes and supplied .dict files give a good number of choices. However, it is fairly easy to create new scoring schemes as .dict files using dictbuilder, one of the utilities supplied with homolmapper (utils/scripts/dictbuilder). Homolmapper also allows the user to define shorthands or mnemonics for various flags or scoring options and to generate smaller working alignments on the fly. As a command-line application, homolmapper is also well suited to automated operation.

6.1 Creating a new scoring scheme

Example directory: FDH
Help flag: --noncanonical (also dictbuilder --help)

Background: Homolmapper is distributed with more than 30 similarity matrices. Three are built-in (BLOSUM62, PAM250, and charge), five are supplied in the lib/ directory (asa, chiS, hphob, scharge, and idmat), and 30 more are supplied as a compressed archive in the lib/ directory. While these provide a wide array of choices, the interested user may wish to define new schemes.

For example, none of the supplied schemes are designed to handle phosphorylation sites. This is because the question is ambiguous; an alignment with mixed bacterial and eukaryotic sequences might need to be evaluated considering all of His, Asp, Ser, Thr, and Tyr as potential phosphorylation sites.

Creating a .list file for dictbuilder: The first step in making a new scoring scheme is to decide what the scores should be. While this step is apparently trivial, it is actually worthwhile to take some time to consider the answer. Using phosphorylation sites as an example, should bacterial phosphorylation sites be considered? Are Ser/Thr kinases or Tyr kinases involved? If there is a specific, multi-residue motif involved, might it be preferable to analyze the alignment using motif scoring?

Once these questions have been answered, the user must also consider how the scheme is to be scored. Something like charge is scored as the value associated with *each amino acid* and would be scored by summing the pairs, so that the -1 of Asp would offset the +1 of Arg. It would also have to be normalized if the user did not want a conserved Arg to come out with a score of +2. On the other hand, a scheme like nonpolar accessible surface area (asa.dict) actually considers the *change* in the properties of each amino acid, so that a value of 0 indicates conservation and each pair of amino acids should be subtracted. To further complicate matters, something like phosphorylation is essentially a binary property: a residue can be phosphorylated or it can't.

Given that the answers to the above questions have been figured out, the user should make a text file with the name of each amino acid and its score as a pair. One such pair is on each line, and the filename should be NAME.list, where NAME will wind up as the name for the scoring scheme. For example, to make a scheme for phosphorylation, a

reasonable name might be psite.list, and the first five lines might look like this were phosphorylated Asp significant for the analysis:

```
Ala     0
Arg     0
Asn     0
Asp     1
Cys     0
```

Lines *beginning with* a '#' character in this .list file will be included in the final .dict file, which provides a good way to document the file at time of creation (a highly recommended procedure). Other comments will not be passed through, but can help to describe individual values.

The choice of what values to put in the .list file is entirely up to the user; homolmapper and dictbuilder will not test them for sanity. If the scoring scheme is physically based, like charge or asa, just listing the values for each amino acid is fine. If it is a binary scheme like phosphorylation, it is best to give it binary values (1 for phosphorylated residue and 0 for all others). Substitution matrices (BLOSUM or PAM matrices, for example) actually have values associated with *pairs* of amino acids rather than *individual* ones; they should not be entered with .list files.

Generating a .dict file with dictbuilder: Given a .list file, generating a .dict file uses the dictbuilder utility (in utils/scripts/ in the standard distribution). One can copy dictbuilder into the present working directory, or put the .list file in with dictbuilder (one could also install dictbuilder in the path, but typical usage is infrequent enough to make this a bit of a waste of time). In any event, dictbuilder requires a .list file and can take several flags to

control how that file is handled. For a scheme like charge, running with the --normalize flag will automatically divide by 2 so that conserved acidic and basic residues get scores of ±1. For a scheme like asa, running with --difference will generate final scores based on the difference between residue values, as desired. A binary scheme like phosphorylation is run with the --binary flag. Assuming a phosphorylation scheme is in psite.list, the command line could thus look like:

```
python dictbuilder psite.list --binary
```

Dictbuilder will check to make sure the final .dict file is the right length. For the standard amino acids, the .list file can use 1-letter or 3-letter code.

Expanded similarity matrices: Dictbuilder can also generate .dict files with expanded amino acid sets . *Non-standard residues can only be handled by dictbuilder as 1-letter codes.* To generate a working matrix with an expanded set, dictbuilder should be run with the --expanded= flag (exactly as for homolmapper itself; thus, the complete flag would be --expanded=U if Cse is ‘U’). The resulting .dict file is ready for use.

Substitution matrices: Dictbuilder can also be used to create substitution matrices (e.g., PAM matrices), but it requires a specialized format derived from specific C header files. This is can handle expanded amino acid sets, but the actual values used in such sets are a complex topic that should be approached with caution. For example, the genetic encoding of Cse residues is not at the level of a single codon, but PAM and BLOSUM matrices incorporate information about codon distribution in the original derivation. It is therefore beyond the scope of this manual to suggest values.

Examples:

1. psite.hmset uses psite.dict (generated from the included psite.list) on the Cse-containing FDH example without expanding the amino acid set. Here are alpha carbons for Tyr10, Asp101, and Cse140 (the latter retains experimental occupancy and B factor):

```
ATOM     69  CA  TYR X  10      76.455  27.201  28.788  1.00  0.83           C
ATOM    784  CA  ASP X 101     102.018  34.735  10.624  0.00  0.83           C
HETATM 1071  CA  CSE X 140      93.012  39.204  31.773  1.00 48.87           C
```

2. selpsite.hmset uses selpsite.dict (generated from the included .list file) and handles CSE with the --interpret= flag rather than a .aalib file. Cse is now scored. Here are the same three alpha carbons as above:

```
ATOM     69  CA  TYR X  10      76.455  27.201  28.788  1.00  0.83           C
ATOM    784  CA  ASP X 101     102.018  34.735  10.624  0.00  0.83           C
HETATM 1071  CA  CSE X 140      93.012  39.204  31.773  0.00  0.00           C
```

Of course, conservation of Ser/Thr/His/Asp/Tyr could occur for reasons other than phosphorylation, such as hydrogen bonding. Here is the structure from this example:

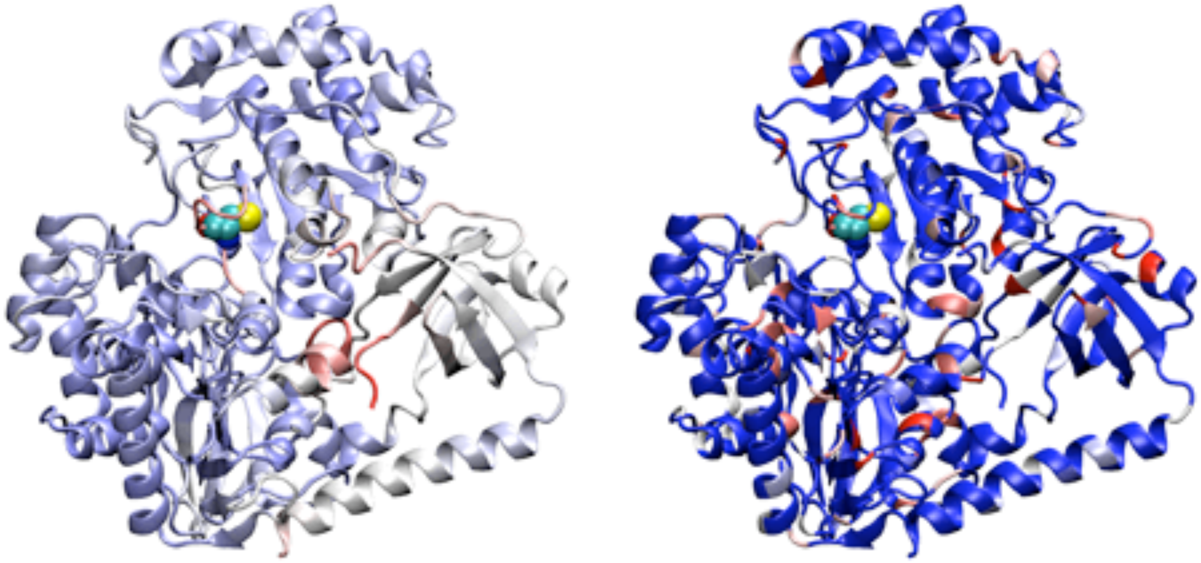

The original B factors are colored from blue to red (left, increasing disorder), while the phosphorylation scheme is colored blue to red (right, increasing conservation). The maximum score with this binary scheme is 1.00.

Additional information:

1. Substitution matrices sometimes assign values to pairs involving nonstandard 1-letter codes. As a general rule, these tend to be used for any standard amino acid, or for a defined subset of the standard 20 (‘B’ for Asp/Asn, or ‘Z’ for Glu/Gln, for example). This makes them dubious as values for nonstandard amino acids. The literature should be consulted to determine whether these codes are appropriate on a case-by-case basis.

2. BLOSUM62 and PAM250 matrices with such “standard” expanded codes are available in the archived additional scoring matrices.

6.2 Customizing command-line parameters

Example directory: cutinase
Help flag: --mnemonics, --scoring (also utils/examples/example.mnemonic)

Background: HomolMapper can present a daunting user interface to those who are not used to command-line applications, with around 75 different flags. It is possible for the user to supply their own names for flags and scoring options. These user-defined synonyms and mnemonics can then be incorporated into .hmset files for easy use.

Supplying synonyms for scoring options: Synonyms can be assigned for options used in the --occ= and --B= scoring lists. This is done from the command line by using the --synonym= flag. This flag takes two arguments: the user name and the option it is replacing. Thus, to use ‘q’ as a shorthand for charge, one could add --synonym=q.charge to the command line and then request --occ=q or --B=q for charge scoring. There are several ‘built-in’ synonyms, listed here:

```
Keyword:            Synonym(s):

gap                 gaps
ins                 insert, inserts, insertion, insertions
len                 length
freq                frequency
sim                 similarity, homology
ID                  identity
entropy             Shannon
mutual              mutual_information
length              len (PSSM only)
vect                vector (PSSM only)
dist                distance (PSSM only)
%                   percent (PSSM only)
```

Customization of flag names: It is also possible to supply custom names for various flags by supplying a file with the extension ‘.mnemonic’ which can contain comments (indicated by ‘#’) and definitions. Each definition is on one line of text and has the user-supplied name followed by white space and then by the default text. Homolmapper will replace all occurrences of the user definition with the default text. For example, using ‘-o’ as a shorthand for ‘--out’ would have convert ‘--overwrite’ to ‘---outverwrite’ (which is so much gibberish to homolmapper, and probably also to the user). A better way to do this would be to set ‘-o=’ as a replacement for ‘--out=’ (shown in the example.mnemonic file). There are a number of ‘built-in’ mnemonics, which are as follows:

```
--occupancy=      --occ=
--temperature=    --B=
--B_field=        --B=
--B_factor=       --B=
--only_report     --nopdb
--no_pdb          --nopdb
--output=         --out=
--extension=      --ext=
--sftol=          --in_tol=
--sfxtol=         --out_tol=
--canonical       --20aa
--strict          --20aa
--match_to=       --use=
--match_with=     --use=
--try=            --use=
--SegID=          --Seg=
--segid=          --Seg=
--seg=            --Seg=
--define_sfam=    --sfam=
--subfamily=      --sfam=
--silent          --verbose=0
--segment         --Seg
--check           --setup
--check_run       --setup
--name            --out
--match_size=     --match=
--union           --sfOR
--mutations=      --mut=
--highlight=      --mut=
--mutation=       --mut=
--motif=          --mut=
--no_statistics   --no_stats
--just_extract    --extract_PDB
--matrix_punch    --punch_matrix
```

Use of .hmset files: One can list favorite customizations in a .hmset file and simply run that file every time. Multiple .hmset files can be used on a command line, and they can be combined with additional flags. Internally, all the arguments from the command line and all .hmset files are built into a single list. Arguments from each .hmset file are inserted at the front of the list, and conflicting settings are resolved by using the last one encountered.

This means that command-line settings will always trump .hmset parameters and that the first .hmset file on the command line will trump subsequent ones in the event of conflicts.

Examples:

1. syn.hmset illustrates the use of the --synonym= flag to allow use of BLOSUM62 and PAM250 for blosum62 and pam250. BLOSUM62 (left) and PAM250 (right) give comparable results in this case (blue, conserved; red, variable).

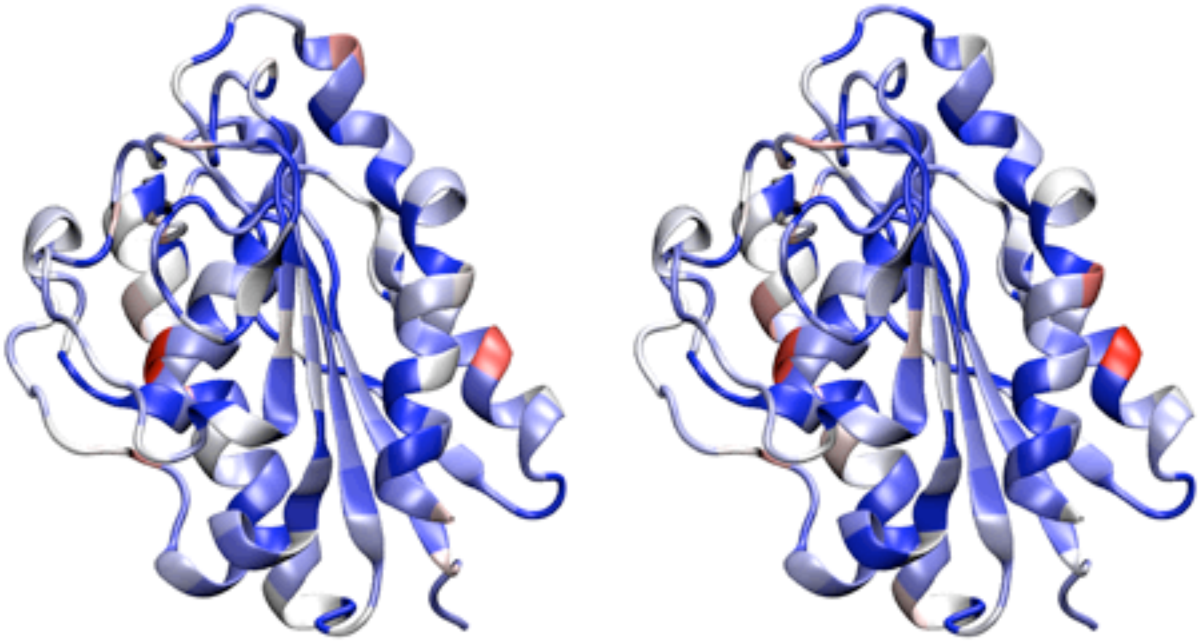

2. mnemonic.hmset uses mnemonic.mnemonic to set --temperature_factor= as an alternative to --B= and uses the built-in alternative --occupancy= for --occ=.

Additional information:

1. A sample .mnemonic file is included in the standard distribution.

2. HomolMapper will also support C-style flags for --help ('-h') and --version ('-v' or '-V').

6.3 Pruning the alignment

Example directory: PcyA
Help flags: --files, --flags (also chapter 4)

Background: Not all alignments are perfect for all applications. The analyses performed by homolmapper typically benefit from larger sample sizes, as with other bioinformatics techniques. Therefore, larger alignments are generally better. However, for some cases the large alignment may be too big, or may contain a subfamily that is confusing the analysis. Of course, one can edit the alignment by hand to remove the extra sequences. As a speedy alternative, homolmapper can discard a subset of sequences from the alignment on the fly. The final alignment can be written out in Clustal format and will include any consensus sequences used as references.

Removing sequences from the alignment: A subset of sequences can be designated for removal with the --discard= flag, which uses the same syntax as the --sfam= flag. Explicit, implicit, and regex definitions are accepted. Sequences that match the definitions are removed before the matching and scoring processes take place, and the number of sequences listed in the header will not include them. By analogy to the .sfam file, one can also use a .disc file for --discard= definitions. The default conventions for combining discard definitions are different than those for combining subfamily definitions, as discussed below.

Combining --discard= definitions: In defining a subfamily (Chapter 4), homolmapper usually includes only those sequences that meet *all* implicit and regex criteria (Boolean AND), but can instead include sequences meeting *either* the implicit or the regex criteria (Boolean OR) with the --sfOR flag. In contrast, when *discarding* sequences, homolmapper will by default remove sequences that match *either* the implicit or the regex criteria (Boolean OR). This difference permits the user to remove multiple spurious groups by default. The --discAND flag will cause homolmapper to instead remove only those sequences that match *all* criteria (Boolean AND). It is also possible to invert the final definition with --discNOT (after explicit sequences have been added to the definition) in the same way that the subfamily definition can be inverted by --sfNOT.

Examining the final alignment: Homolmapper can write out a CLUSTAL-format alignment file from the input MSA file. Discarded sequences will be absent. If a consensus sequence is used as a reference sequence in scoring, it will be interleaved into the alignment. There will be a one-line, CLUSTAL-compatible header indicating that the file was generated by homolmapper. There will be no ".:*" lines. If consensus sequences are used as references with --setup, the alignment will be punched automatically.

Examples:

1. simple.hmset uses a single regex definition to exclude the members of a distant subfamily. 10 sequences are discarded, yielding 60 in the final alignment. Here are alpha carbons for Asp105 with the starting (top) and pruned alignments (bottom):

```
ATOM   1679  CA  ASP A 105       5.198 -21.726  43.417  0.00 80.00           C
ATOM   1679  CA  ASP A 105       5.198 -21.726  43.417  0.00 93.33           C
```

2. complex.hmset uses three definitions to remove all but three subfamilies (56 total sequences). The above regex definition is used in combination with a single implicit definition that removes all members of a different subfamily except for a single one which is removed explicitly. Here is the Asp105 alpha carbon:

```
ATOM   1679  CA  ASP A 105       5.198 -21.726  43.417  0.00 100.0           C
```

The same final alignment could have been generated in other ways. For example, alternate.hmset achieves the same result by using two regex definitions with Booleans set to NOT in combination with --discAND (this example also uses a .disc file). The resulting PDB file differs only in the timestamp line of the header. Here is the structure, colored by percent identity for the entire alignment (left) and the alternate pruned alignment (alternate.pdb, right). Several scores are slightly higher with the pruned alignment.

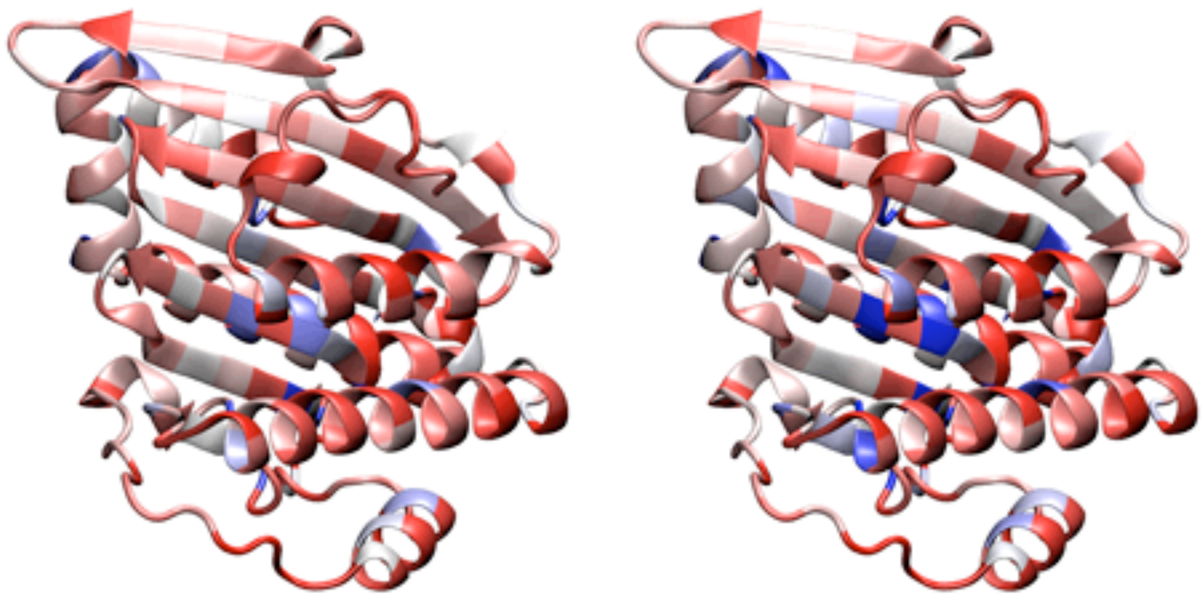

Additional information:

1. Subfamily definitions and discards can be combined in the same run.

2. Errors will be triggered if the discard definition will not remove anything and if there are no sequences left after application of the --discard= definitions.

6.4 Automation and scripting

Example directory: phytochrome
Help flags: --files, --flags

Background: One of the advantages of a command-line tool such as homolmapper is the relative ease of running it repetitively without any need for the user to click dialog boxes and so forth. Homolmapper itself has several features to facilitate such automation and to permit the results to be analyzed by other programs.

Working with .hmset files: The examples in this tutorial have made extensive use of .hmset files. Such files can be combined with additional flags and scripts to set up a series of runs. The settings used for any particular run can also be saved at runtime by running with the --punch=run flag, which will cause homolmapper to write a .hmset file with the current settings. Filenames will be commented out so that the same settings can then be used on other files if so desired, and the standard homolmapper header will be written. One can edit the result to use it as a template to run a single structure with many alignments, a single alignment with many structures, or other cases.

.report files: In addition to the output PDB file, homolmapper can also generate a tabular file reporting the scored residues and their scores as tab-delimited text. This can then be opened by spreadsheet or graphing programs or can be fed into other scripts for post-processing. The standard homolmapper header is added, as is a title line, and the residues are sorted by chain and residue number. Any SegID scores are reported, and the correlation between MSA and .pdb numbering is also reported (equivalent to match numbering with --Seg=numbering). The --nopdb flag generates only the .report file.

Scaling the output scores: It is possible to force the output score values to range between values chosen by the user, for instance to keep scores in a good range for "sausage" representations of protein structure (varying spline radius). This can be done with --autoscale (which will turn on autoscaling for both occupancy and B factor) or by adding "autoscale" to the --occ= and/or --B= lists (e.g., --B=pam250.autoscale), which will only turn it on for the scheme for which it is listed. The default scaling will cause all scores to fall within 0.2 and 1.0, inclusive, but this range can be set with --low= and/or --high= flags. A single scaling range can be set per run. By default, charge scores are scaled as the absolute value; this can be disabled with --nOaBS. Autoscaling will automatically turn on the --zero flag (setting scores for unscored atoms to 0.0).

Examples:

1. The settings in BvCE.hmset will generate a .report file using entropy scoring in combination with a .slop file containing set definitions proposed by Mirny & Shakhnovich (*JMB* **291:** 177). The resulting .report file is well suited to graphing the scores against each

other (here, occupancy is insertion frequency and B-factor is entropy). Such a plot is shown here. Strikingly, positions with gap frequency > 20% are also higher than the mean entropy (as one might expect if gaps tend to appear in variable surface loops).

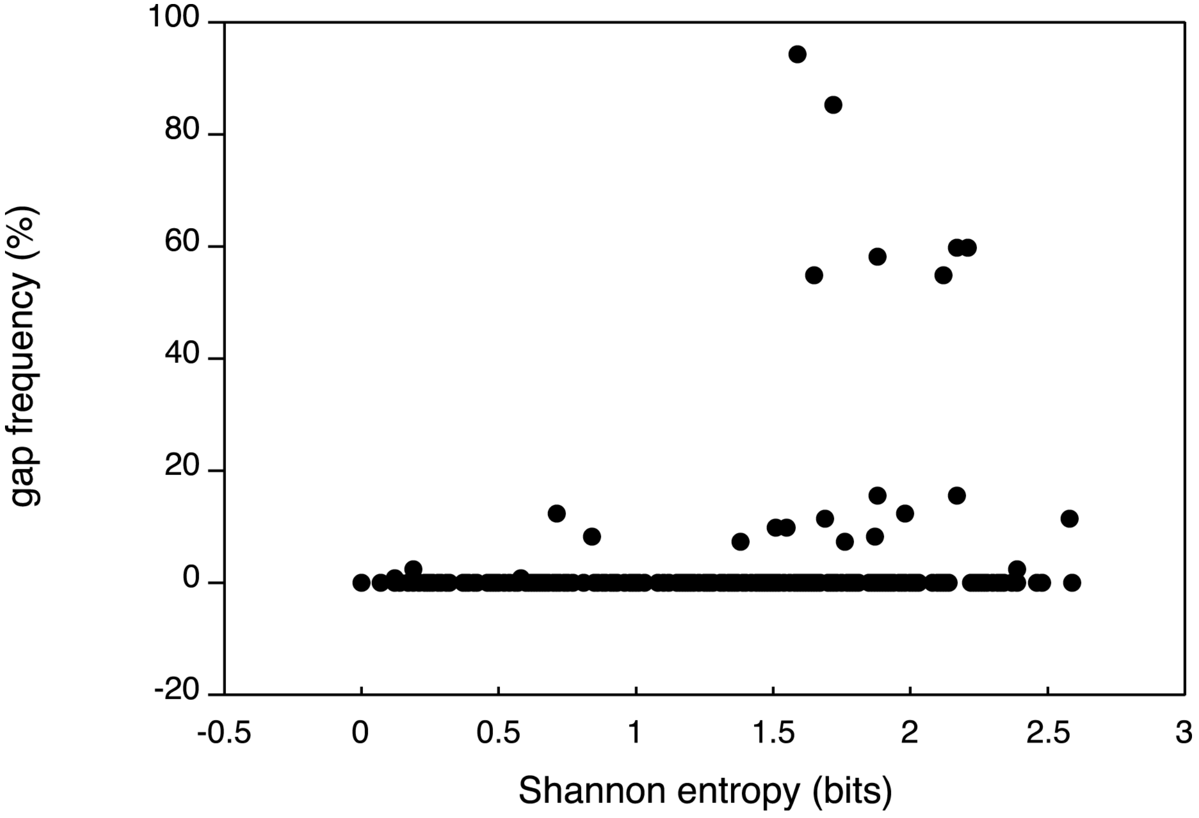

Additional information:

1. Autoscale in combination with sausage representations can present homolmapper results without reliance on color.

3. Settings in .hmset files do not need quotes (and won't work right with them). Quotes on the command line protect against reinterpretation by the shell, and .hmset files are handled within homolmapper itself.

Chapter 7: Under the hood

Overview: Homolmapper presents a wide range of options. It also uses several corrections in the course of normal operations. This chapter discusses the header written to homolmapper output files, which is used to keep track of key settings, and then examines calculation of identity and various normalization schemes. Finally, a brief troubleshooting section is presented.

7.1 Homolmapper headers

Example directory: cutinase
Help flag: --flags

Background: To help keep track of which settings are used to prepare which output file, homolmapper writes a brief header to each output file. This header consists of twelve REMARK records that describe the run. Here is a sample header:

```
REMARK  This file generated by   homolmapper version 16.7.4
REMARK  using 1UBQ.pdb, ubiqs.aln
REMARK  ubiqs.aln: 3 sequences. Matched: 1UBQ (76 aa).
REMARK  occupancy: gap  len            structure
REMARK  B-factor: sim   ID             structure
REMARK  SegID:  (none)
REMARK  occupancy statistics: mean,  0.01; SD,  0.11; score diversity,  1.62
REMARK  B-factor statistics: mean, 57.46; SD, 20.66; score diversity, 21.25
REMARK  Thu Mar  1 13:56:18 2007       Runtime: 0.04 sec.
REMARK
REMARK
REMARK
```

The first line lists the version of homolmapper used in the run. The second lists the input filenames (and can list accessory files as well). The third line describes the MSA file and

indicates which sequence in the MSA was matched to the .pdb sequence and how many residues were matched. If sequences were discarded, the name of the .disc file (or --disc if no .disc file was used) is shown on this line as well. The next 2 lines describe the scoring settings that were used for occupancy and B factor. The next line describes any settings used for SegID. The occupancy and B-factor score statistics are listed on the next two lines, and the next line has a timestamp indicating when the run took place and the runtime from the start of the run to the creation of the header (typically ≤ 0.1 second less than the total run time). The next line can either be blank (as here) or can contain a complete list of detected subfamily-specific residues with sfspec scoring. The next-to-last line can either be blank or can contain a user-specified comment. The last line is usually left blank; if the element field was used, a description of the use to which it was put will appear here.

Adding a comment to the output file: Homolmapper can incorporate a user comment into the output header. The comment is added with the --comment= flag, which may need to be placed within quotes if the comment includes whitespace or other characters that can cause shell problems. For example, adding '--comment=This is my run.' (enclosed in single or double quotes) to the above example results in the following header:

```
REMARK  This file generated by   homolmapper version 16.7.4
REMARK  using 1UBQ.pdb, ubiqs.aln
REMARK  ubiqs.aln: 3 sequences. Matched: 1UBQ (76 aa).
REMARK  occupancy: gap  len            structure
REMARK  B-factor: sim   ID             structure
REMARK  SegID:  (none)
REMARK  occupancy statistics: mean,  0.01; SD,  0.11; score diversity,  1.62
REMARK  B-factor statistics: mean, 57.46; SD, 20.66; score diversity, 21.25
REMARK  Thu Mar  1 13:56:18 2007       Runtime: 0.04 sec.
REMARK
REMARK   This is my run.
REMARK
```

Viewing all subfamily-specific residues: The algorithm for detecting subfamily-specific residues will not evaluate positions that are aligned with gaps in the matched sequence. However, all other positions are evaluated, regardless of whether that part of the matched sequence is actually in the structure. The complete list of detected residues is placed in the header in the line below the timestamp.

Example:

1. header.hmset illustrates a range of optional material in headers. Here is the header:

```
REMARK  This file generated by   homolmapper version 16.7.4
REMARK  using 2AXE.pdb, cutinase.aln    --sfam(2)  1 .aalib used.
REMARK  cutinase.aln: 3 sequences. Matched: PpAXE (207 aa). --disc
REMARK  occupancy: sim  sfspec  DE
REMARK  B-factor: sim   degen   ACDEFHIKLMNQRSTVWY      structure
REMARK  SegID: --mut (motif)
REMARK  occupancy statistics: mean,  0.06; SD,  0.23; score diversity,  3.93
REMARK  B-factor statistics: mean, 83.19; SD, 32.50; score diversity, 15.07
REMARK  Thu Mar  1 22:13:48 2007        Runtime: 0.16 sec.
REMARK  DE: 21, 25, 39, 66, 121, 124, 132, 160, 170, 180, 199, 202, 220, ;
REMARK   This is a full header.
REMARK  Element: motif ID
```

In this example, a subfamily is specified, as is a .aalib file for PDB translation (both indicated on line 2). A .sfam filename would appear instead of --sfam were one supplied. The size of the subfamily is indicated in parentheses (line 2). A discard was supplied on the command line (line 3); a .disc filename would appear instead had one been supplied. The scoring options are an sfspec call (which lists the amino acid set used in searching, line 4) and a degen call (which again lists the set used, line 5). A motif request is made (line 6); the --mut indicates command-line input, and would be replaced by a filename had a .mut file been supplied. Scoring statistics are on line 7 for occupancy and line 8 for B-

factor. The timestamp is on line 9, and the hits from sfspec are on line 10. These are listed in the amino acid numbering of the matched sequence, not that of the structure itself. This is an important distinction in this case, because the sequences are not the same, so the numbering is different. A user comment is present (line 11). Line 12 notes that the element field reports motif ID in this case (more than one motif was defined).

Additional information:

1. If both a .sfam file and --sfam= are supplied, only the .sfam filename and the total subfamily size will be reported.

2. If both a .mut file and --mut= are supplied, only the filename will be reported. The scoring option used for SegID will always be reported.

3. The --comment= flag must currently appear on its own line in any .hmset file.

4. Mutual-information runs use different approaches to scoring, so they have different headers as described in Chapter 3.

5. Similar header files are added to other outputs (.report, .hmset, and .sfam). The header lines in .hmset and .sfam files are commented out (a '#' is added as the first character on each line), so that the resulting files can be reused by homolmapper. The header lines are not currently commented out in .report files, so scripts which use them should handle all lines beginning with 'REMARK' differently. The .report format also writes a title line after the REMARK lines and before the actual values.

7.2 Identity identified

Example directory: cutinase

Help flag: --scoring

Background: Identity scoring is a frequently used but somewhat ambiguous term. If one is interested in finding conserved insertions in one subfamily relative to a global alignment, one would want to only consider the identity within those sequences that possess residues in the insertions of interest. However, in a more general case, one would want to assess conservation while penalizing for sequences that lack amino acids. Homolmapper by default penalizes for gaps when scoring by identity, matrix-lookup, and user-defined-set scores (degen or sloppy). There is no need to do so while evaluating subfamily-specific residues, and gap penalties are irrelevant for scoring by gaps or insertions.

Controlling gap penalties: It is possible to turn off the gap penalty in identity scoring with the --no_gap_penalty flag. This flag will disable the gap penalty for all similarity calculations using the percent identity algorithm (e.g., --B=ID), any of the matrix-lookup schemes (e.g., --B=pam250), and either of the algorithms available for user-defined amino acid sets (degen and sloppy scoring).

Example:

1. Here is part of the cutinase alignment, with a region of interest in bold:

```
PpAXE       SQGGEIMDVALCGGGDPNQGYTNTAVQLSSSAVNMVKAAIFMGDPMFRAGLSYEVGTCAA
AfAXE       SQGGQIMDNALCGGGDPNQGYTNTAVPLSSSAVNMIKAAIFMGDPRYVAGLPYNVGTCKA
NcCUT       SQGGQIMDNAVCGGPDSGAGITTSTPGINASALNQVKAVIMMGNPRYRAGLSYNVGTCTA
mycAXE2     SQGAQVATDTICGGAG---DPFTSDKGMSDDVMDDVVAVAIFGDPTHVANMTYDRGTSIH
            ***.::   ::*** .   .  .:   :. ..:: : *. ::*:* . *.:.*: **.
```

Here are the alpha carbons for the underlined region after running with percent identity scoring (B factor, def_ID.hmset):

```
ATOM    707  CA  ASP   105       8.216  -6.936   8.195 75.00 75.00           C
ATOM    715  CA  PRO   106       7.233  -9.517   5.627 50.00 50.00           C
ATOM    722  CA  ASN   107       7.700  -7.243   2.665 50.00 50.00           C
ATOM    730  CA  GLN   108      11.314  -6.690   3.606 50.00 50.00           C
ATOM    739  CA  GLY   109      12.046 -10.330   4.397 75.00 75.00           C
```

The scores of the first and last residues are 75% (3 out of 4), while the scores of the middle 3 are 50% (2 out of 4) because the gap sequence is counted. However, using no_gap_ID.hmset gives a different set of scores. Here are the same five alpha carbons:

```
ATOM    707  CA  ASP   105       8.216  -6.936   8.195 75.00 75.00           C
ATOM    715  CA  PRO   106       7.233  -9.517   5.627 66.67 66.67           C
ATOM    722  CA  ASN   107       7.700  -7.243   2.665 66.67 66.67           C
ATOM    730  CA  GLN   108      11.314  -6.690   3.606 66.67 66.67           C
ATOM    739  CA  GLY   109      12.046 -10.330   4.397 75.00 75.00           C
```

The first and last are still scored as 75%, but the other residues are scored at 67% because the sequence with a gap is ignored. Here are views of these two outputs, with the gap penalty off (left) or on (right). The small surface loop at the lower right hand corner is rare in the alignment but is highly conserved when present, so it appears much more conserved without a gap penalty.

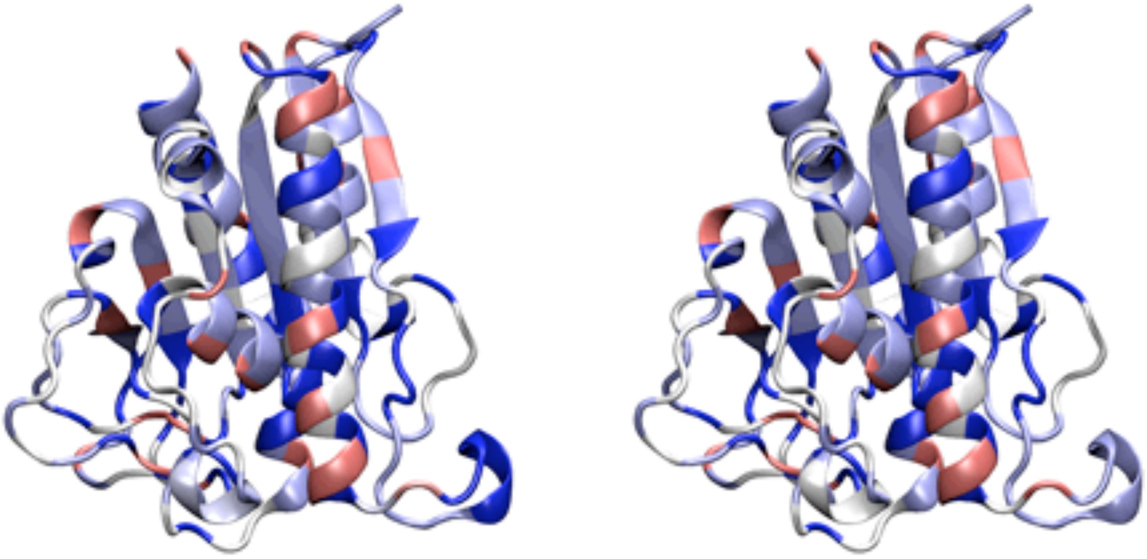

Additional information:

1. Alternate reference sequences (e.g. a consensus sequence) will not change the score at positions where the matched sequence matches the reference sequence.

2. The occupancy field in these examples is scored with the identity matrix, which gives the same results. Using sloppy scoring without a .slop file will also give the same results. There are thus four ways to score by identity: default identity scoring, identity matrices loaded as .dict and .mat files, and sloppy scoring with no .slop file. This provides a useful way to validate any changes a user might want to make to homolmapper itself.

7.3 Normalization and correction

Example directory: none
Help flag: --flags

Background: There are several corrections built into homolmapper. Scores are normally normalized for the size of the alignment; similarity (matrix) schemes are also normalized so that perfectly conserved residues all get the same score. There are flags that can disable these normalizations at need. *They are not necessary or recommended for normal use.* Because of this, they are deliberately given names that are hard to type.

Normalization for number of sequences: Homolmapper matrix-based scoring schemes are implemented as a series of pairwise comparisons to the reference sequence at each position in the alignment where the matched sequence does not have a gap. The pairwise comparisons are summed and then divided by the number of sequences in the group

being scored (the alignment or the subfamily). If the --rAWsIM flag is specified, the sum of the pairwise comparisons is instead divided by 1. Scores generated in this way are extremely sensitive to the size of the alignment.

Normalization of similarity matrices: Substitution matrices such as the BLOSUM and PAM series assign different values to different residues for perfect conservation. For example, the BLOSUM62 matrix assigns a score of 4 to a perfectly conserved Ala but a score of 8 to a perfectly conserved His. While this is a deliberate feature of many such matrices, it is by no means necessary. It is also confusing for structural examination; for example, a score of 4 could mean an informative, absolutely conserved Ala or a less interesting, variable His, and inspection of a PDB file output by homolmapper would not discriminate between the two without the use of range scoring.

Homolmapper therefore normalizes such matrices such that absolutely conserved residues are given the same score (100.0). Such normalization is only applied to schemes that return a nonzero value for absolute conservation of all residues, because it is implemented by dividing by the score for an absolutely conserved residue. It is therefore irrelevant for many physical schemes. It is disabled by the --nOnORM flag.

Scoring by number of residues: It is also possible to score simply by counting the residues that are present at each position. This scheme is extremely sensitive to the size of the alignment. It is invoked by combining identity in --occ= or --B= with the --nrES flag.

Additional information:

1. Homolmapper does not currently score positions that are gaps in the matched sequence. This reflects its bias towards generating files for structural display, because such positions by definition cannot be mapped onto the structure for display.

2. One of the schemes in the more_scores group uses frequency of occurrence of amino acids in proteins. This matrix actually produces more interpretable results when run using --nOnORM, as is noted in the comments in the aa_freq.dict file.

7.4 Troubleshooting

Example directory: proteasome
Help flags: --files, --flags

Background: There are cases that will trigger errors within homolmapper. For instance, running homolmapper on the proteasome example with the peptide-matching algorithm (chosen with --skip=2) will trigger an error. In this example, of course, the error is avoided with the default settings, because the chain-matching algorithm will find a match before the peptide-matching algorithm is called. However, not all cases will end so happily. Most errors trigger specific error messages giving information about the nature of the problem.

Error messages: There are two kinds of errors that can occur during homolmapper runs: those that trigger custom messages, and those that trigger standard Python error messages. The latter can occur after a warning message. Custom error messages have a standard traceback (see below) but end with a line starting with “ERROR:” followed by

information about the nature of the problem. Both types are written to stderr and hence will still be reported with --verbose=0.

Warning messages: Homolmapper can also trigger warnings. Warnings are not errors and are usually triggered when something unusual happens of which the user should be aware. For example, if an imported .dict file is not the correct size, a warning will be triggered. Warnings are indicated by “WARNING:” and are written to stdout, so they do not appear with --verbose=0. Warnings are converted into errors by the --sensitive flag.

Stop messages: Homolmapper terminates early if either --setup or --nopdb are specified on the command line. With these flags, homolmapper prints a stop line to stderr starting with “STOP: “ and indicating which flag triggered the premature stop.

Verbosity: Homolmapper can print varying amounts of information to the screen during the run. In the event of errors or unexpected results, it can frequently be worthwhile to simply repeat the run at a higher level of output to watch what’s going wrong (perhaps with --overwrite if output is being generated).

--setup runs: The --setup flag is especially useful for cases arising from problems with the MSA file and for testing complicated subfamily or discard definitions.

Examining generated subsets with the --punch= flag: The --punch= flag can take several arguments. One can generate a new .hmset file with --punch=run, see a defined

subfamily with --punch=sfam, or examine the final working alignment with --punch=aln. One can also see a consensus sequence by itself with --punch=cons. The sequence of the PDB file can be punched in FASTA format with --punch=pdbaa. Finally, --punch=all will cause all of these files to be written. Multiple choices can also be chosen by specifying a list of choices separated by '.' characters (e.g., running with --punch=aln.sfam generates the same files that can be generated with --setup).

--Seg=numbering: This option provides the most detail about the result of matching residues in the structure to positions in the alignment. Each residue that is scored has the matched amino acid number written to SegID (the amino acid in the matched sequence that was scored to that residue, starting from the number 1 and not counting gaps). Discontinuities or unexpected numbering in the SegID column are informative.

Examples:

1. Any Python error message includes a traceback of the sequence of events leading to the error. The actual error message is at the bottom of this stuff. The following is a trivial example of a custom homolmapper error: the input .pdb file does not exist.

Command line:

```
homolmapper foo.pdb bar.aln
```

Stdout information:

none

Stderr information:

```
Traceback (most recent call last):
  File "/usr/local/bin/homolmapper", line 4751, in ?
```

```
    [pdb, aln, res, occ_str, B_str] = parse_n_setup(args, res)
  File "/usr/local/bin/homolmapper", line 1685, in parse_n_setup
    filename_check(file_tuple)
  File "/usr/local/bin/homolmapper", line 1708, in filename_check
    raise 'ERROR: input .pdb file not found.'
ERROR: input .pdb file not found.
```

The key here is the last line: a message indicates that the .pdb file could not be found.

2. By definition, gaps and insertions are only scored relative to the structure sequence. Therefore, if a subfamily does not contain the structure sequence and gap or insertion scoring is requested, an error is triggered. gap_err.hmset is such a case.

Command line:

```
homolmapper gap_err.hmset
```

Stdout information:

```
Chosen sequence (amino acid overlap)
pre5 ( 233 )


Working subfamily:      2
```

Stderr information:

```
Traceback (most recent call last):
  File "/usr/local/bin/homolmapper", line 4763, in ?
    occlist, blist, seglist = driver(scoring_tuple, aln_tuple, goodind)
  File "/usr/local/bin/homolmapper", line 2966, in driver
    raise e_s, c_s
ERROR: structure not within subfamily: required for gaps or insertions.
```

In this example, a match is made and the subfamily definition proceeds normally. Both produce messages to stdout. However, the error is then triggered, resulting in messages to stderr (note that both go to the screen unless steps are taken to separate the two streams; they are separated here for clarity). Again, the last line explains the problem.

3. An example without an error is gap_warn.hmset, which requests gap scoring relative to a consensus sequence. Gaps and insertions are only scored relative to the matched sequence (i.e. the structure sequence). However, this is not a fatal error; the program simply reverts to the default case and reports a warning.

Command line:

```
homolmapper gap_warn.hmset
```

Stdout information:

```

        Chosen sequence (amino acid overlap)
        pre5 ( 233 )

        Calculating consensus using single-aa defs.
gap len consensus
WARNING: Gaps/insertions scored relative to structure.
        Calculating gap scores by length.

        Complete.
```

Stderr information:

none.

The WARNING line indicates that gaps and insertions are scored relative to the structure (the requested scoring scheme is indicated above). Note that adding --sensitive to the command line will change the warning into an error:

Command line:

```
homolmapper gap_warn.hmset --sensitive
```

Stdout information:

```

        Chosen sequence (amino acid overlap)
        pre5 ( 233 )
```

```
        Calculating consensus using single-aa defs.
gap len consensus
```

Stderr information:

```
Traceback (most recent call last):
  File "homolmapper", line 4765, in ?
    occlist, blist, seglist = driver(scoring_tuple, aln_tuple, goodind)
  File "homolmapper", line 3063, in driver
    therapy(ckstr)
  File "homolmapper", line 1895, in therapy
    raise out_string
ERROR: Gaps/insertions scored relative to structure.
```

Here, the same problem is reported as an error rather than a warning. This can be useful if many runs are being done automatically with --verbose=0 suppressing warnings and can be helpful in catching problems that are otherwise hard to diagnose.

Additional information:

1. By default, homolmapper will trigger an error rather than overwriting a file. This behavior can be suppressed by the --overwrite flag and is also turned off by --setup.

2. When a nonstandard amino acid is not being handled in parsing the alignment with a matrix-lookup scoring scheme, a KeyError is triggered.

3. The --extract_PDB flag will punch the PDB and shut down. No MSA or PSSM is required. Any PDB sequence punched out in FASTA format will by default have the usual header; this is suppressed by the --clean_pdbaa flag.

4. There is a contact email at the homolmapper home page and in the standard homolmapper README. It can be used, but please try a few things first….
